# Supplementary material for: Functional connectivity–based classification and subtyping of major depression for precision mental health: An ensemble graph neural network approach
Source: PLOS Digit Health. 2026 Mar 4;5(3):e0001261. doi: 10.1371/journal.pdig.0001261 (PMC12959711; doi:10.1371/journal.pdig.0001261)
Supplement: S1 Text — (DOCX) [file pdig.0001261.s001.docx]

**Supplementary Tables**

**Table A.** Demographic and clinical characteristics of patients with MDD and HC subjects for each site in REST-meta-MDD.

| **Site** | **MDD** | | | |  | **HC** | | | |  | **All** | | | |
| --- | --- | --- | --- | --- | --- | --- | --- | --- | --- | --- | --- | --- | --- | --- |
|  | **Number** | **Male/**  **Female** | **Age(y)** | **Edu(y)** |  | **Number** | **Male/**  **Female** | **Age(y)** | **Edu(y)** |  | **Number** | **Male/**  **Female** | **Age(y)** | **Edu(y)** |
| Site1 | 73 | 30/43 | 31.9 ± 8.1 | 13.8 ± 3.0 |  | 73 | 32/41 | 31.7 ± 9.0 | 15.2 ± 2.2 |  | 146 | 62/84 | 31.8 ± 8.5 | 14.5 ± 2.7 |
| Site2 | 16 | 1/15 | 41.8 ± 11.5 | 11.6 ± 4.5 |  | 14 | 4/10 | 45.6 ± 12.1 | 10.0 ± 4.8 |  | 30 | 5/25 | 43.5 ± 11.7 | 10.8 ± 4.6 |
| Site4 | 18 | 8/10 | 31.7 ± 7.6 | 14.2 ± 3.5 |  | 23 | 11/12 | 28.8 ± 4.7 | 14.9 ± 2.7 |  | 41 | 19/22 | 30.0 ± 6.2 | 14.6 ± 3.1 |
| Site7 | 35 | 13/22 | 42.0 ± 11.7 | 15.0 ± 4.1 |  | 37 | 14/23 | 38.2 ± 11.8 | 14.9 ± 3.5 |  | 72 | 27/45 | 40.0 ± 11.8 | 13.1 ± 4.5 |
| Site8 | 39 | 9/30 | 31.9 ± 9.8 | 11.1 ± 3.6 |  | 48 | 21/27 | 31.9 ± 10.7 | 13.0 ± 2.5 |  | 87 | 30/57 | 31.9 ± 10.3 | 12.1 ± 3.2 |
| Site9 | 48 | 22/26 | 28.6 ± 8.7 | 13.4 ± 2.9 |  | 48 | 30/18 | 28.6 ± 8.0 | 15.9 ± 2.8 |  | 96 | 52/44 | 28.6 ± 8.3 | 14.7 ± 3.1 |
| Site10 | 45 | 21/24 | 32.7 ± 10.8 | 11.3 ± 3.1 |  | 26 | 17/9 | 32.7 ± 10.8 | 12.8 ± 2.0 |  | 71 | 38/33 | 32.7 ± 9.8 | 11.9 ± 2.8 |
| Site11 | 20 | 9/11 | 30.2 ± 9.3 | 11.2 ± 3.0 |  | 17 | 8/9 | 31.4 ± 9.6 | 15.6 ± 2.5 |  | 37 | 17/20 | 30.8 ± 9.3 | 13.2 ± 3.6 |
| Site13 | 20 | 8/12 | 32.7 ± 8.6 | 13.7 ± 2.2 |  | 16 | 5/11 | 34.4 ± 10.7 | 13.3 ± 2.3 |  | 36 | 13/23 | 33.4 ± 9.5 | 13.5 ± 2.2 |
| Site14 | 61 | 19/42 | 30.1 ± 7.0 | 13.7 ± 3.3 |  | 32 | 15/17 | 29.6 ± 5.0 | 14.6 ± 2.8 |  | 93 | 34/59 | 29.9 ± 6.3 | 14.0 ± 3.2 |
| Site15 | 30 | 9/21 | 46.5 ± 12.6 | 11.1 ± 3.8 |  | 37 | 17/20 | 39.8 ± 14.7 | 13.1 ± 3.8 |  | 67 | 26/41 | 42.8 ± 14.1 | 12.2 ± 3.9 |
| Site17 | 41 | 14/27 | 21.7 ± 3.0 | 13.1 ± 1.5 |  | 41 | 13/28 | 20.6 ± 1.8 | 13.8 ± 1.6 |  | 82 | 27/55 | 21.2­­ ± 14.1 | 12.2 ± 3.9 |
| Site19 | 18 | 5/13 | 35.0 ± 11.4 | 9.7 ± 3.1 |  | 31 | 14/17 | 35.2 ± 10.2 | 9.9 ± 3.9 |  | 49 | 19/30 | 35.1­­ ± 10.6 | 9.8 ± 3.6 |
| Site20 | 245 | 80/165 | 38.4 ± 11.7 | 11.0 ± 3.4 |  | 225 | 69/156 | 39.5 ± 15.8 | 13.1 ± 3.8 |  | 470 | 149/321 | 38.9­­ ± 13.8 | 12.0 ± 3.7 |
| Site21 | 79 | 34/45 | 34.1 ± 12.1 | 11.8 ± 2.7 |  | 65 | 28/37 | 36.5 ± 12.5 | 13.0 ± 2.1 |  | 144 | 62/82 | 35.2­­ ± 12.3 | 12.3 ± 2.5 |
| Site22 | 18 | 9/9 | 33.8 ± 9.8 | 12.0 ± 3.0 |  | 20 | 12/8 | 24.4 ± 7.1 | 13.3 ± 2.1 |  | 38 | 21/17 | 28.8 ± 9.6 | 12.7 ± 2.6 |
| Site23 | 22 | 10/12 | 26.2 ± 7.4 | 13.9 ± 3.2 |  | 23 | 8/15 | 33.0 ± 12.0 | 14.3 ± 4.1 |  | 45 | 18/27 | 29.7 ± 10.5 | 14.1 ± 3.6 |

**Table B.** Demographic and clinical characteristics of patients with MDD and HC subjects for each site in SRPBS.

| **Site** | **MDD** | | |  | **HC** | | |  | **All** | | |
| --- | --- | --- | --- | --- | --- | --- | --- | --- | --- | --- | --- |
|  | **Number** | **Male/**  **Female** | **Age(y)** |  | **Number** | **Male/**  **Female** | **Age(y)** |  | **Number** | **Male/**  **Female** | **Age(y)** |
| HUH | 57 | 32/25 | 43.3 ± 12.1 |  | 67 | 29/38 | 34.8 ± 12.9 |  | 124 | 61/63 | 38.7 ± 13.2 |
| HRC | 16 | 6/10 | 40.5 ± 11.1 |  | 49 | 13/36 | 41.7 ± 11.5 |  | 65 | 19/46 | 41.4 ± 11.4 |
| HKH | 33 | 20/13 | 44.8 ± 11.3 |  | 29 | 12/17 | 45.4 ± 9.4 |  | 62 | 32/30 | 45.1 ± 10.4 |
| COI | 71 | 31/40 | 45.2 ± 12.4 |  | 124 | 46/78 | 51.9 ± 13.4 |  | 195 | 77/118 | 49.4 ± 13.4 |

**Table C. Data acquisition parameters of the REST-meta-MDD dataset.**

| **Sites** | **Scanner** | **Receive (coil)** | **TR (ms)** | **TE (ms)** | **Flip Angle (∘)** | **FOV** |
| --- | --- | --- | --- | --- | --- | --- |
| **1** | Siemens Tim Trio 3T | 32 channel | 2000 | 30 | 90 | 210 × 210 |
| **2** | Philips Achieva 3T | 8-channel | 2000 | 30 | 90 | 240 × 240 |
| **3** | Siemens Magnetom Symphony scanner 1.5 T | 16 channel | 2000 | 40 | 90 | 240 × 240 |
| **4** | GE discovery MR750 | 8 channel | 2000 | 30 | 90 | 220 × 220 |
| **5** | GE Signa 3T | 8 channel | 2000 | 30 | 90 | 240 × 240 |
| **6** | GE Discovery MR750 3.0T | 8-channel | 2000 | 25 | 90 | 240 × 240 |
| **7** | Siemens Tim Trio 3T | 32 channel | 2000 | 30 | 90 | 240 × 240 |
| **8** | GE Signa 3T | 8 channel | 2000 | 30 | 90 | 240 × 240 |
| **9** | GE Excite 1.5T | 16 channel | 2500 | 35 | 90 | 256 × 256 |
| **10** | Siemens Tim Trio 3T | 32 channel | 2500 | 25 | 90 | 240 × 240 |
| **11** | Siemens Verio 3.0T MRI | 12 channel | 2000 | 25 | 90 | 240 × 240 |
| **12** | GE Signa 3T | 8 channel | 2000 | 40 | 90 | 240 × 240 |
| **13** | GE Signa 3T | 8 channel | 2000 | 22.5 | 30 | 220 × 220 |
| **14** | Siemens Tim Trio 3T | 12 channel | 2000 | 30 | 90 | 220 × 220 |
| **15** | Siemens Tim Trio 3T | 32 channel | 2000 | 30ms | 90 | 200 × 200 |
| **16** | Philips Gyroscan Achieva 3.0T | 32 channel | 2000 | 30 | 90 | 240 × 240 |
| **17** | Philips Achieva 3.0T TX | 8 channal | 2000 | 30 | 90 | 240 × 240 |

**Table D. Consistent subtype-specific brain connections on both datasets.**

|  | **Principal Dataset** | | **Independent validation dataset** | |
| --- | --- | --- | --- | --- |
|  | *Connection between* | | *Connection between* | |
| Subtype1 | Precuneus_L | Precuneus _R | Precuneus _L | Precuneus _R |
|  | Paracentral_lobule_L | Paracentral_lobule _R | Paracentral_lobule_L | Paracentral_lobule _R |
|  | Thalamus_L | Thalamus _R | Thalamus _L | Thalamus_R |
|  | Cerebelum_6_L | Cerebelum _6_R | Cerebelum _6_L | Cerebelum _6_R |
|  | Cerebelum _9_L | Cerebelum _9_R | Cerebelum _9_L | Cerebelum _9_R |
| Subtype2 | Insula_L | Insula_R | Insula_L | Insula_R |
|  | Cingulum_Ant_L | Cingulum_Ant _R | Cingulum_Ant _L | Cingulum_Ant _R |
|  | Cingulum_Post_L | Cingulum_Post_R | Cingulum_Post_L | Cingulum_Post_R |
|  | Thalamus_L | Thalamus _R | Thalamus _L | Thalamus_R |
|  | Temporal_Sup_L | Temporal_Sup_R | Temporal_Sup_L | Temporal_Sup_R |
|  | Cuneus_L | Cuneus_R | Cuneus_L | Cuneus_R |
|  | Cerebelum_6_L | Cerebelum _6_R | Cerebelum _6_L | Cerebelum _6_R |
| Subtype3 | Frontal_Sup_L | Frontal_Sup_Medial_L | Frontal_Sup_L | Frontal_Sup_Medial_L |
|  | Cuneus_L | Cuneus_R | Cuneus_L | Cuneus_R |
|  | Paracentral_lobule_L | Paracentral_lobule _R | Paracentral_lobule_L | Paracentral_lobule _R |
|  | Caudate_L | Caudate_R | Caudate_L | Caudate_R |
|  | Putamen_L | Putamen_R | Putamen_L | Putamen_R |
|  | Thalamus_L | Thalamus _R | Thalamus _L | Thalamus_R |

**Table E The impact of graph construction threshold on leave-one-site cross-validation.**

| **Threshold** | **10%** | **15%** | **20%** | **25%** | **30%** |
| --- | --- | --- | --- | --- | --- |
| **Site1** | 0.56 | 0.55 | **0.63** | **0.63** | **0.63** |
| **Site2** | 0.73 | 0.70 | **0.80** | 0.67 | 0.67 |
| **Site3** | 0.80 | 0.73 | **0.88** | 0.71 | 0.71 |
| **Site4** | 0.72 | 0.71 | **0.75** | 0.69 | 0.68 |
| **Site5** | 0.67 | 0.68 | **0.70** | 0.64 | 0.67 |
| **Site6** | 0.64 | 0.63 | **0.69** | 0.68 | 0.63 |
| **Site7** | 0.72 | 0.66 | **0.75** | 0.65 | 0.68 |
| **Site8** | **0.73** | **0.73** | 0.70 | 0.65 | 0.62 |
| **Site9** | **0.78** | 0.75 | **0.78** | **0.78** | **0.78** |
| **Site10** | 0.68 | 0.69 | **0.72** | 0.71 | 0.67 |
| **Site11** | 0.82 | 0.81 | 0.84 | **0.85** | 0.84 |
| **Site12** | 0.62 | 0.61 | **0.67** | 0.61 | 0.65 |
| **Site13** | 0.65 | 0.69 | **0.73** | 0.69 | 0.65 |
| **Site14** | 0.60 | 0.59 | **0.63** | 0.59 | 0.60 |
| **Site15** | 0.55 | 0.59 | **0.68** | 0.59 | 0.61 |
| **Site16** | 0.61 | 0.53 | **0.76** | 0.59 | 0.63 |
| **Site17** | 0.64 | 0.64 | 0.64 | **0.73** | **0.73** |
| **Mean** | 0.68 | 0.66 | **0.73** | 0.67 | 0.67 |

**Table F The impact of graph construction threshold on generalization performance in the independent validation dataset using the principal dataset.**

| **Threshold** | **10%** | **15%** | **20%** | **25%** | **30%** |
| --- | --- | --- | --- | --- | --- |
| **HUH** | 0.65 | 0.65 | **0.73** | 0.66 | 0.63 |
| **HRC** | 0.72 | 0.74 | 0.74 | **0.75** | **0.75** |
| **HKH** | 0.63 | 0.60 | **0.66** | 0.63 | **0.66** |
| **COI** | 0.64 | 0.62 | **0.66** | 0.65 | 0.60 |
| **Mean** | 0.66 | 0.65 | **0.70** | 0.67 | 0.66 |

**Table G Ablation study of loss components (cross-national validation).**

| $L_{mask}$ | $L_{sps}$ | $L_{ent}$ | Accuracy |
| --- | --- | --- | --- |
| 🗴 | 🗴 | 🗴 | 0.61 |
| 🗸 | 🗴 | 🗴 | 0.67 |
| 🗴 | 🗸 | 🗴 | 0.61 |
| 🗴 | 🗴 | 🗸 | 0.64 |
| 🗸 | 🗸 | 🗴 | 0.63 |
| 🗴 | 🗸 | 🗸 | 0.61 |
| 🗸 | 🗴 | 🗸 | 0.66 |
| 🗸 | 🗸 | 🗸 | 0.73 |
