# Supplementary figures and images for: Functional connectivity–based classification and subtyping of major depression for precision mental health: An ensemble graph neural network approach
Source: PLOS Digit Health. 2026 Mar 4;5(3):e0001261. doi: 10.1371/journal.pdig.0001261 (PMC12959711; doi:10.1371/journal.pdig.0001261)

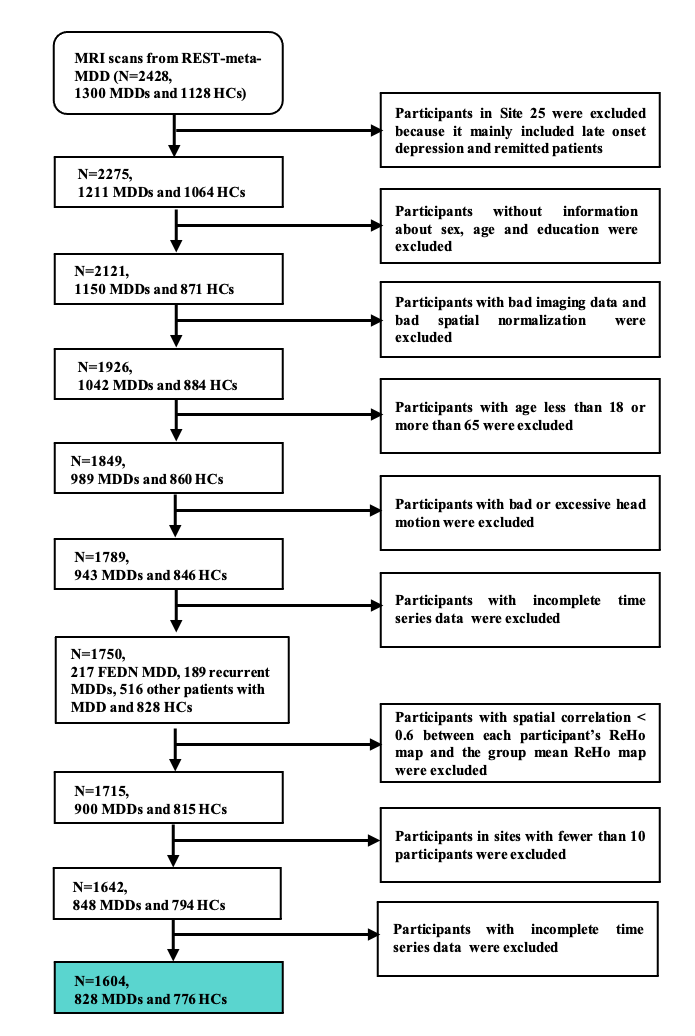

Supplement: S1 Fig — From 2428 subjects, 1604 subjects were selected through above criteria. (TIF) [file pdig.0001261.s002.tif]

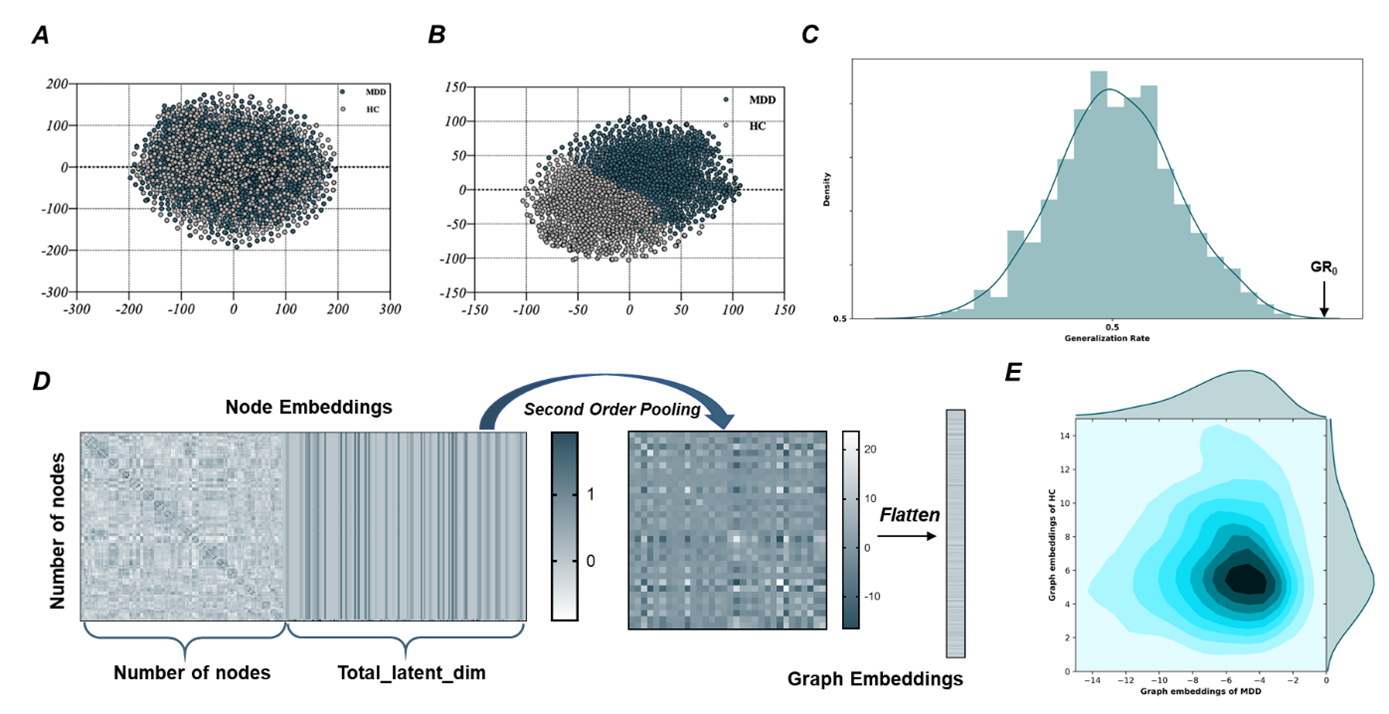

Supplement: S2 Fig — (A) FC networks from REST-meta-MDD cohort of 1604 participants were used as inputs and a two-dimensional plot was generated using the t-SNE, where the dark green represented the patients with MDD and the gray represented healthy controls. (B) Procedure of obtaining the graph embeddings from node embeddings in a single participant is depicted. Total_latent_dim = the number of nodes + the number of hidden units × (the number of GNN layers -1). (C) Permutation distribution of the estimate using the trained EH-BrainGNN model (repetition times: 1000) are shown, where x- and y-labels represent the generalization rate and probability density. GR0 denotes the generation rate gained by the EH-BrainGNN model trained on the real class labels. (D) Graph embeddings that served as inputs were embedded in a two-dimensional plot generated using the t-SNE for the two classes (MDD and HC). (E) The kernel distributions of graph embeddings of the MDD and HC populations are depicted. (TIF) [file pdig.0001261.s003.tif]

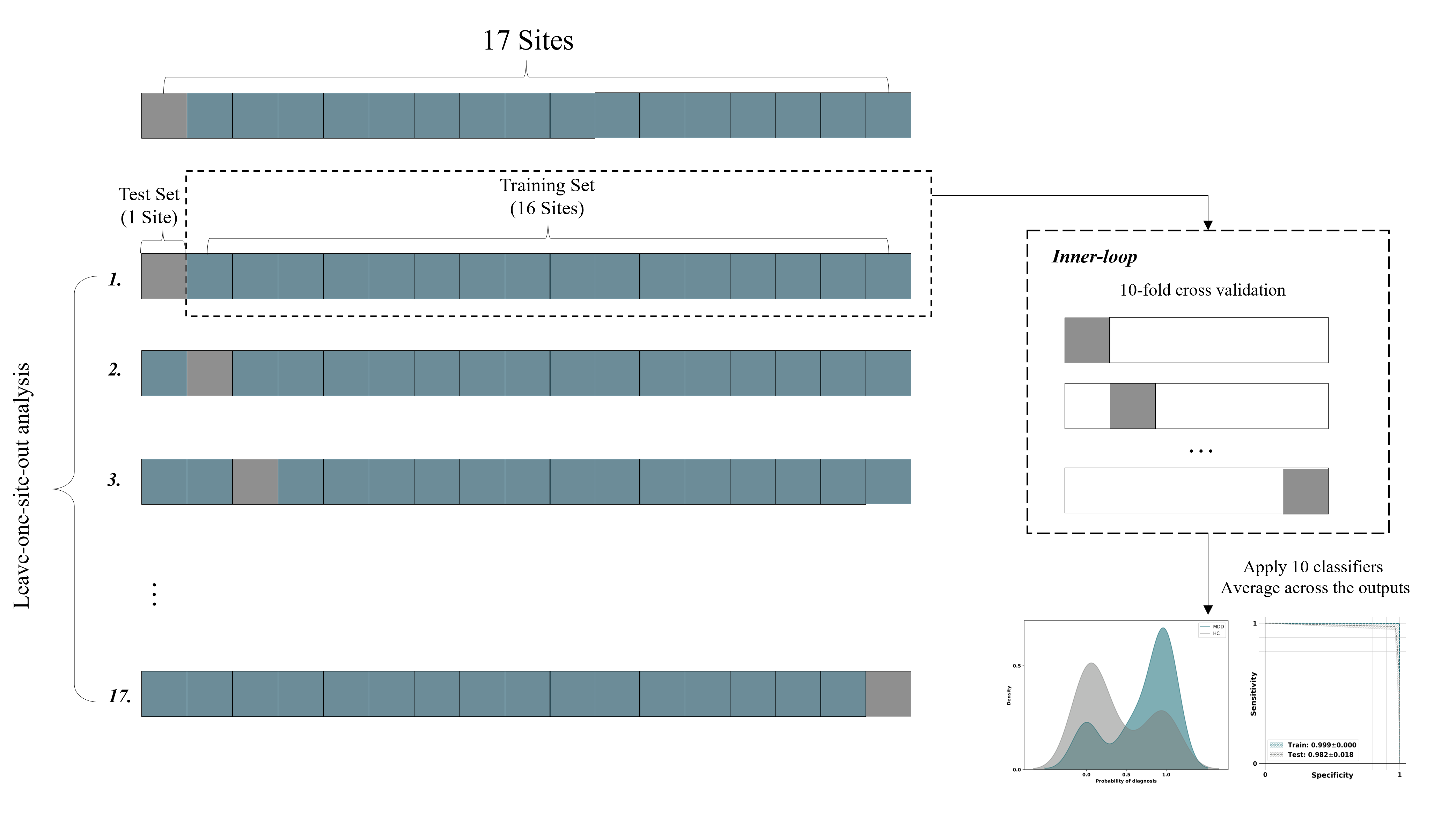

Supplement: S3 Fig — (TIF) [file pdig.0001261.s004.tif]

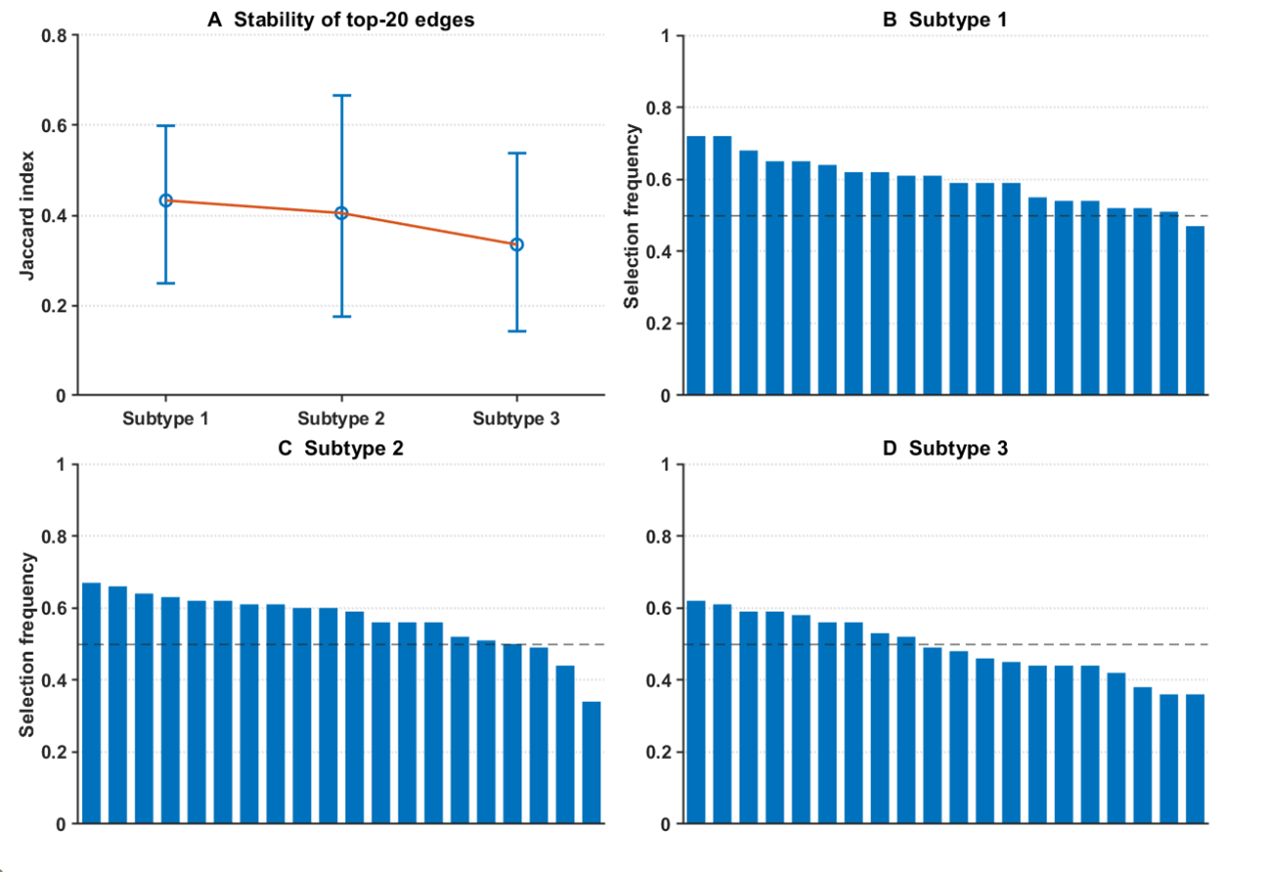

Supplement: S4 Fig — (A) the stability of the subtype-specific top-20 edges across bootstrap resamples quantified by the Jaccard index. (B) the bootstrap selection frequency of the reference top-20 edges for each subtype. (TIF) [file pdig.0001261.s005.tif]

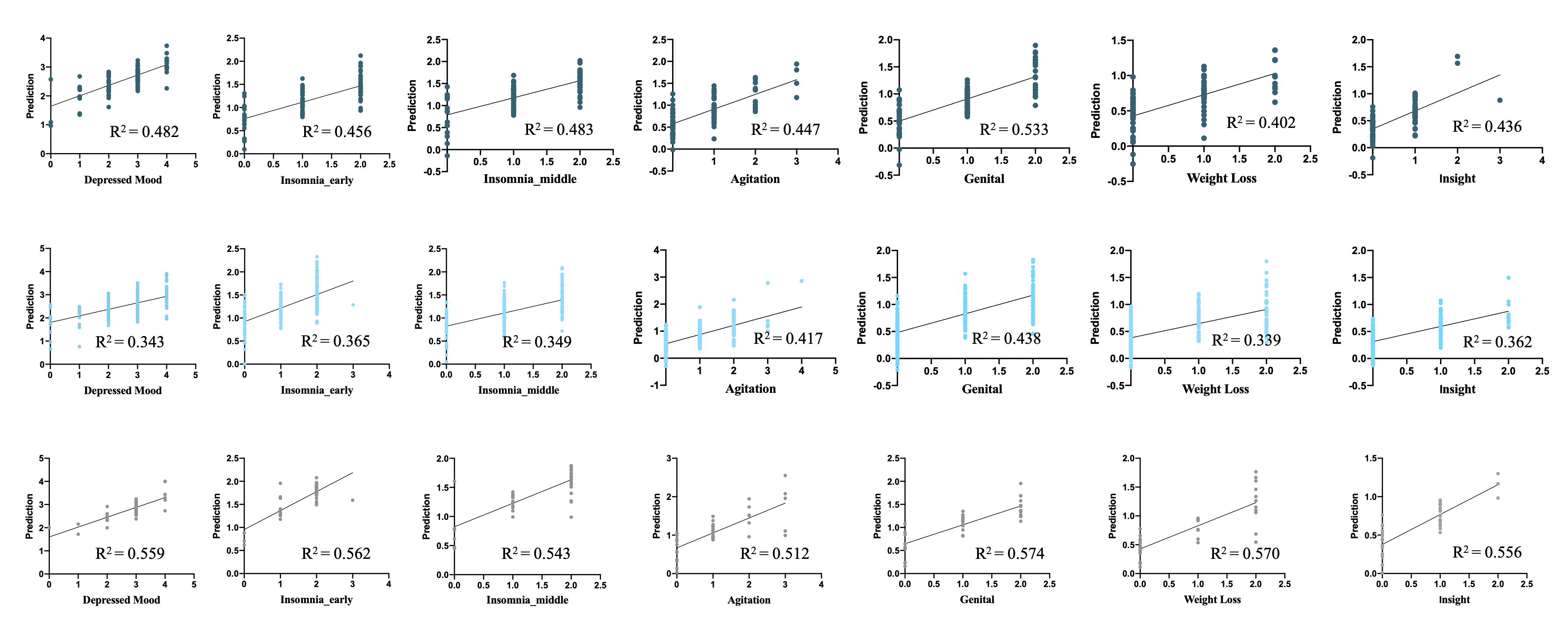

Supplement: S5 Fig — (TIF) [file pdig.0001261.s006.tif]

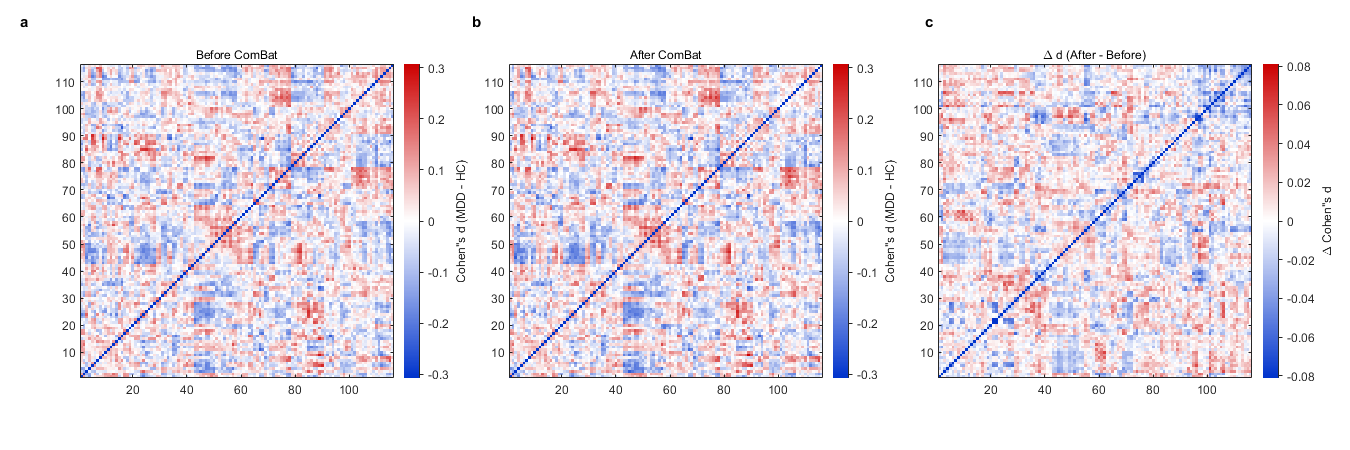

Supplement: S6 Fig — (TIF) [file pdig.0001261.s007.tif]

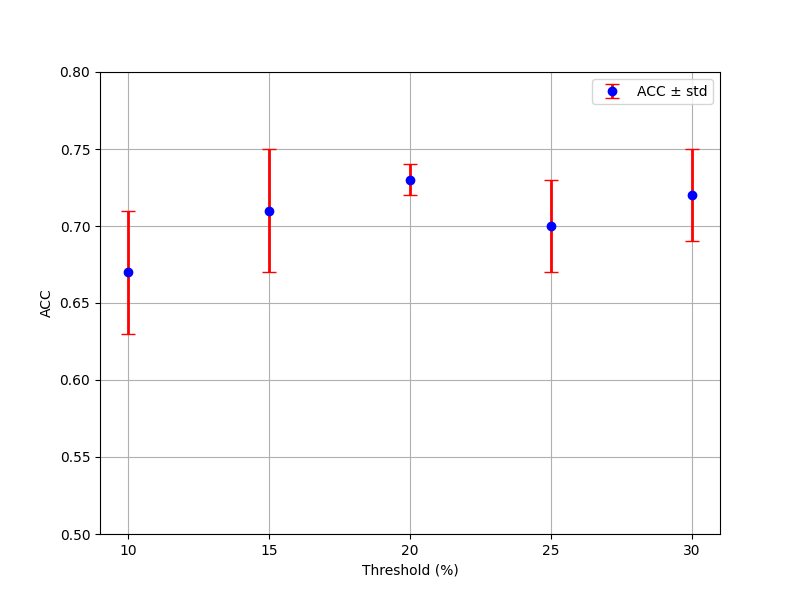

Supplement: S7 Fig — (TIF) [file pdig.0001261.s008.tif]

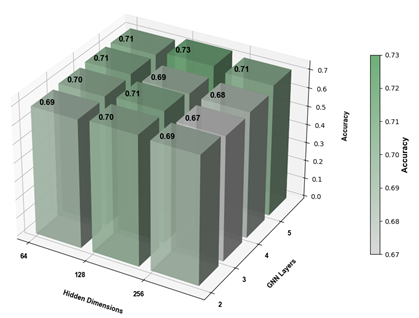

Supplement: S8 Fig — (TIF) [file pdig.0001261.s009.tif]

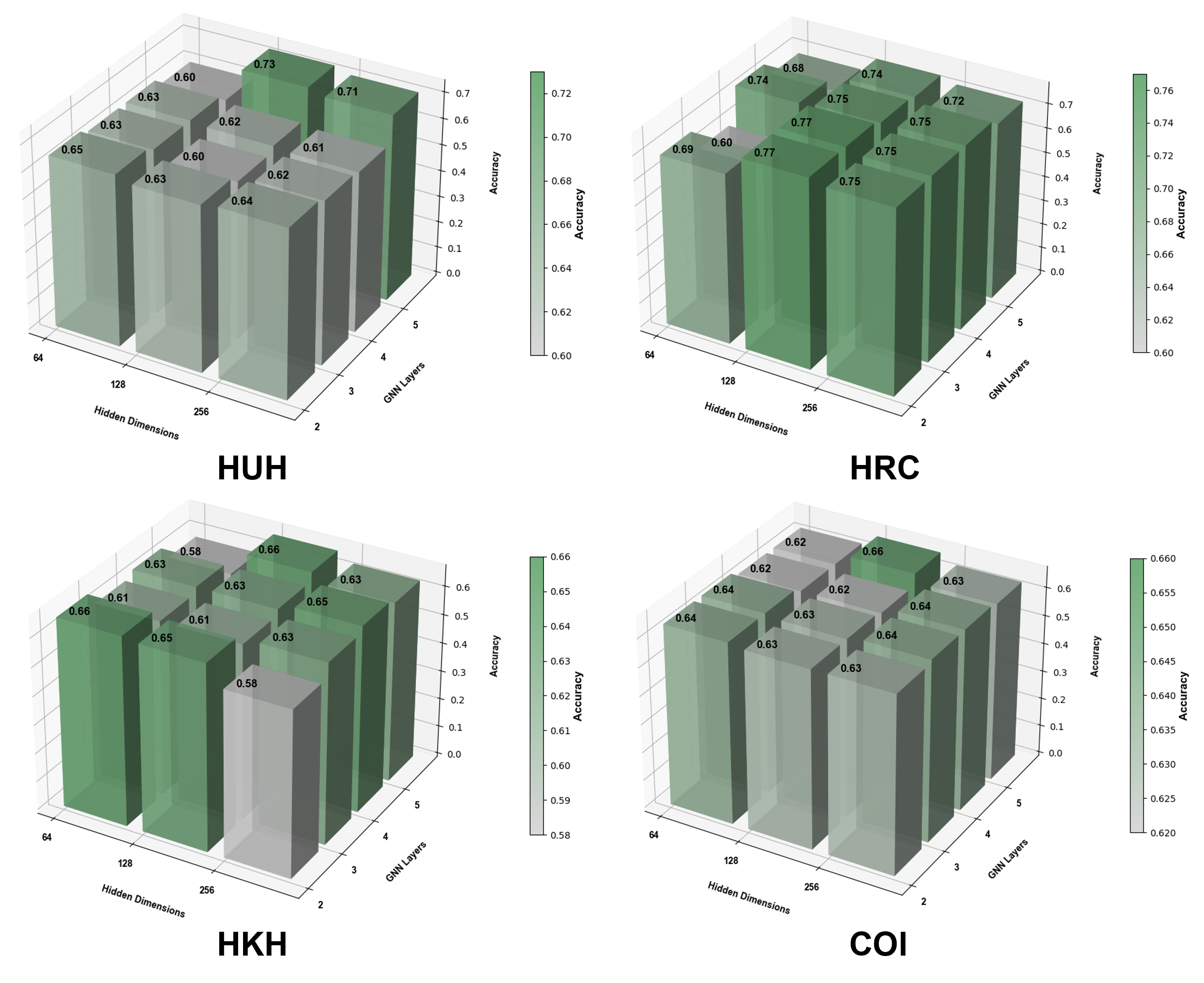

Supplement: S9 Fig — (TIF) [file pdig.0001261.s010.tif]

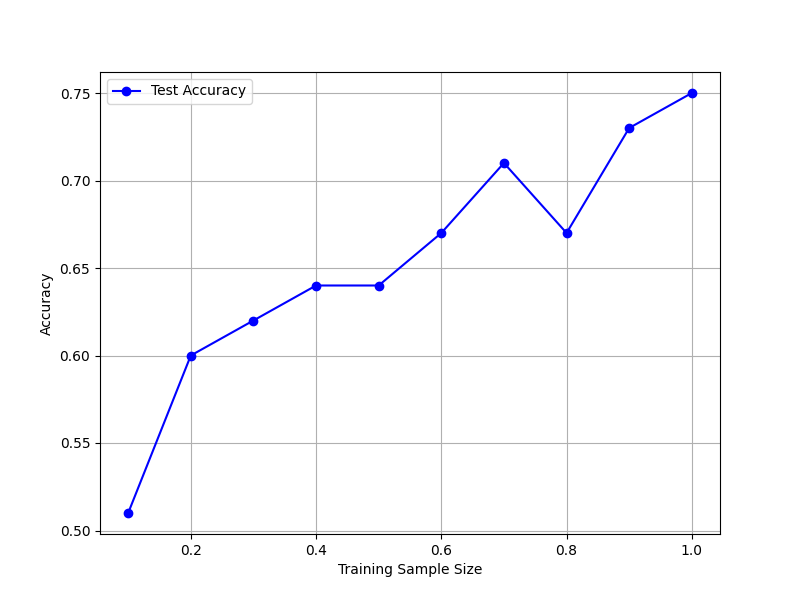

Supplement: S10 Fig — (TIF) [file pdig.0001261.s011.tif]

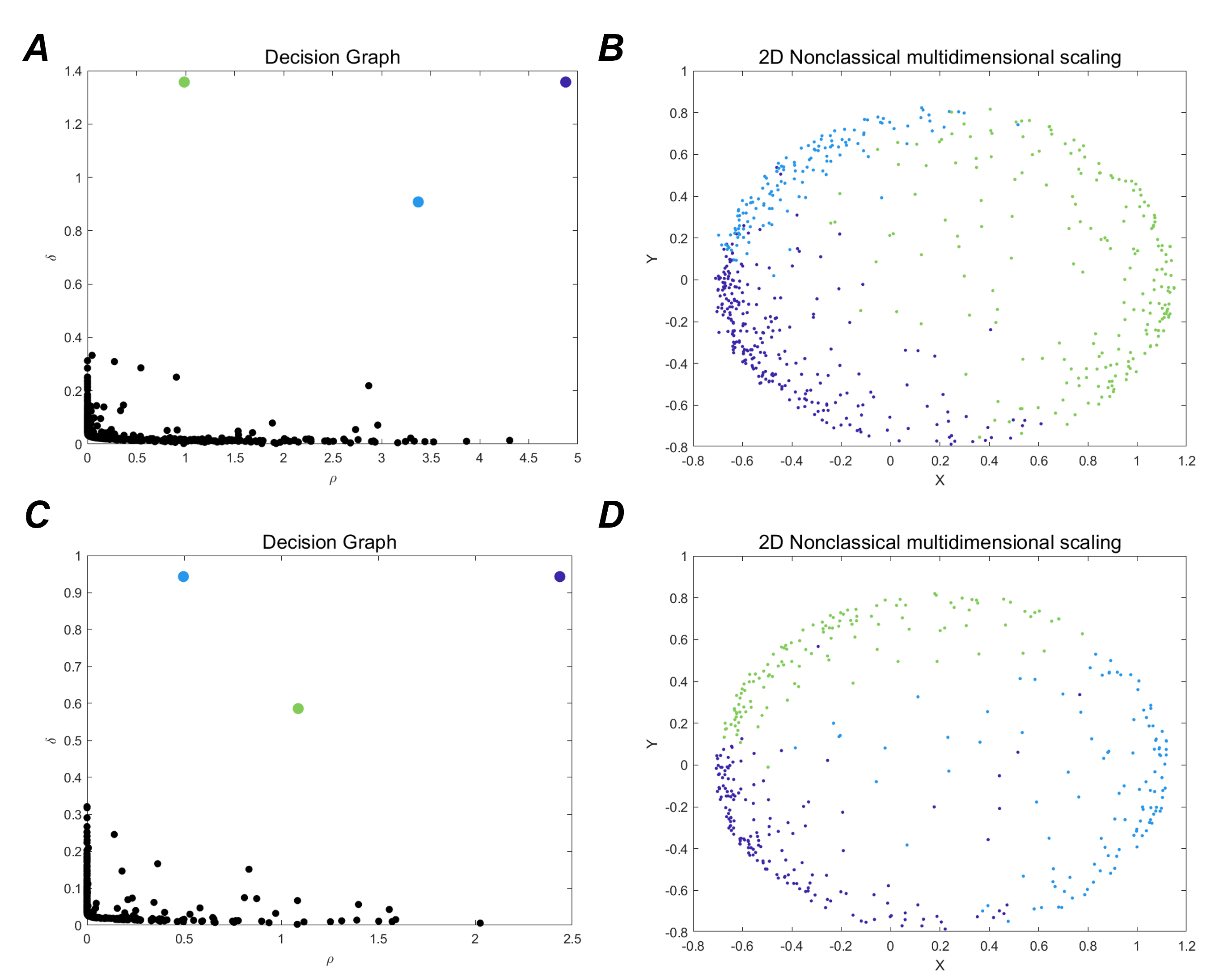

Supplement: S11 Fig — (A-B) CFDP decision graph and spatial distribution of subtypes with medication included as a covariate. (C-D) CFDP decision graph and spatial distribution of subtypes with medication-naïve participants. (TIF) [file pdig.0001261.s012.tif]
