# Supplementary material for: Functional connectivity–based classification and subtyping of major depression for precision mental health: An ensemble graph neural network approach
Source: PLOS Digit Health. 2026 Mar 4;5(3):e0001261. doi: 10.1371/journal.pdig.0001261 (PMC12959711; doi:10.1371/journal.pdig.0001261)
Supplement: Attachment — Submitted filename: reviwer comments sept 2025.pdf [file pdig.0001261.s014.pdf]

1. Clarity and Grammar

The manuscript has many grammatical errors, awkward phrases and typos that impede readability. Examples include:

- The duplicate "the" in the Methods: "Standard preprocessing … were done using the the Data Processing Assistant for Resting-State fMRI (DPARSF)".
- An ungrammatical phrase "In regarding to the unsupervised subtyping model"; this should read "Regarding the unsupervised subtyping model…".
- Sentences with dangling modifiers or missing articles, e.g., "To mitigate distinct sites, artifact or confounding effect, we adopted ComBat harmonization method" (should be "To mitigate site differences and confounding effects…").
- Typographical errors such as "reedifying" instead of "redefining" in the Discussion, and "response" instead of "respond".
- In the Discussion, "different biological mechanisms underline different subtypes" (should be "underlie") and "the importance of identifying diagnostic biomarker for MDD subtypes have long been recognized, by the solution is still unclear" (missing conjunction).
- Several run-on sentences, such as the claim that "EH-BrainGNN achieved the highest average generalization performance of 73% in most sites compared with other baselines and yielded significant and consistent improvements, suggesting an advantage of our study that the EH-BrainGNN was able to generalize well on the completely different multi-sites even if site-specific difference".
- Use of the verb "access" instead of "assess" when stating future plans: "apply rTMS to different subtypes and access the treatment efficacy".
- The abstract and title contain minor spacing errors ("majordepression" and "networkapproach").

Recommendation: Engage a professional editor or a native English speaker to proofread and revise the manuscript. Ensure subject–verb agreement, correct tense usage, and proper word choice throughout.

2. Methodological Details and Justification

The study uses an ensemble hybrid framework (EH-BrainGNN) combining a supervised graph neural network with unsupervised clustering, but several methodological choices lack justification or clarity.

1. Data description and cohort differences:
    - The paper states the principal dataset contains 1604 participants (848 MDD, 794 controls) from 17 sites and the independent validation dataset contains 446 participants (177 MDD, 269 controls) from 4 sites. However, there is little discussion of differences in demographics, scanning parameters, or cultural factors between Chinese and Japanese sites that could influence functional connectivity. How were these variables controlled beyond ComBat harmonization?

2. ComBat harmonization:
    - ComBat is used to control site effects, but the description is copied from the original formula and lacks clarity (equations (1)–(2) are presented without explaining all variables). There is also a grammatical error "using uses Empirical Bayes". The authors need to explain why ComBat is appropriate, whether other harmonization methods were considered, and provide evidence that it does not remove biological variation relevant to MDD.
3. Graph construction:
    - The FC matrix is binarized by setting the top 20-percentile absolute correlation values to one. This threshold appears arbitrary. How robust are the results to different thresholds (e.g., 10%, 15%, weighted networks)? A sensitivity analysis is needed.
4. Feature representation:
    - Node features are defined as the correlation of each ROI with all other ROIs. This leads to high dimensionality; were there any feature reduction steps? Also, the authors refer to "subgraph generator" with an edge mask learned via the Concrete distribution but do not state how this ensures interpretability or whether the learned mask is consistent across folds.
5. Mutual information estimator:
    - The mutual information loss functions (equations (9)–(12)) are presented but not well explained. Key hyperparameters ($\lambda 1$, $\lambda 2$, $\lambda 3$) controlling sparsity and compactness are not specified in the main text.
6. Training and hyperparameter selection:
    - The paper mentions using 10-fold cross-validation with an Adam optimizer, dropout and weight decay. However, the grid search space for hyperparameters is only referred to in Table 2 (not provided in the manuscript excerpt). Without detailed hyperparameter ranges and final values, reproducibility is limited.
7. Baseline comparisons:
    - The authors compare EH-BrainGNN with SVMs, LASSO, Random Forest, and several GNN variants. Yet, the paper does not describe how these models were tuned or whether the training/test splits were identical. Statistical tests comparing performance (e.g., McNemar test, paired t-test on fold results) are not reported.
8. Class imbalance:
    - With 848 MDD and 794 controls in the training set, class balance is acceptable, but the validation set has 177 MDD and 269 controls. Did the authors use balanced accuracy or F1-scores to account for imbalance? They report only accuracy and AUC.
9. Cross-site validation:
    - The leave-one-site-out analysis across 17 sites is appropriate, but the authors should provide standard errors or confidence intervals for the 73% average

accuracy. They also claim the highest performance is 88% at one site without identifying the site; such details are necessary.

10. Unsupervised clustering:
    - The CFDP algorithm automatically selects the number of clusters. The paper reports three subtypes representing 25.9%, 62.1% and 12.1% of patients. However, cluster stability is not assessed (e.g., using bootstrapping or consensus clustering). It is unclear whether the same patients fall into the same subtype across folds. The authors also do not report silhouette scores or other metrics to support three clusters.
11. Statistical analysis of subtypes:
    - Wilcoxon rank-sum tests with FDR correction are used to compare HAMD symptoms across subtypes. The manuscript does not specify the number of tests performed (nine HAMD items?) and the correction method (Benjamini–Hochberg?). Additionally, p=0.066 is reported as a "reduced pattern" for BDI scores, which is not statistically significant.
12. Interpretability claims:
    - The authors describe EH-BrainGNN as "self-explaining" and claim that the learned subgraphs provide interpretability. Yet there is no quantitative assessment of interpretability (e.g., overlap with known depression networks, comparison with expert-defined ROIs) or demonstration of how clinicians might use these explanations.
13. Reproducibility and code availability:
    - The paper states that data from the REST-meta-MDD and SRPBS datasets are publicly available, but there is no statement about code availability. For a deep-learning study, sharing code and trained models is essential for reproducibility.

Recommendation: Provide comprehensive methodological details, justify key choices (e.g., thresholding, hyperparameters), add robustness analyses, report statistical tests for baseline comparisons, and make code publicly available.

3. Results Presentation

- Many results are described without corresponding figures or tables in the main text (e.g., Table 3, Figures 3–5, Table 5). For a reader to judge the claims, these visuals must be accessible.
- The text repeatedly refers to accuracy alone; sensitivity, specificity, precision, recall and F1-score should be reported. The authors also need to clarify whether 74%/73% accuracy is sufficiently high for clinical translation.
- The t-SNE plots used to demonstrate graph embedding separation are sensitive to random initialization. Provide multiple runs or report more robust dimensionality reduction methods (e.g., UMAP).

- When reporting p-values, specify whether tests are one-sided or two-sided and include effect sizes.

4. Discussion and Interpretation
   - The Discussion repeats results but provides limited clinical context. For example, how might the three subtypes inform personalized treatment or prognosis? Are there known symptom clusters or biological markers that align with these subtypes? How do the findings relate to previous work by Drysdale et al. or Wang et al.? A more comprehensive comparison with existing subtyping frameworks is needed.
   - The authors acknowledge that treatment response could not be assessed because only baseline data were available. This limitation should be emphasized earlier in the abstract and conclusions.
   - The potential impact of cultural differences between Chinese and Japanese cohorts is not discussed. Differences in diet, lifestyle, or clinical practice may influence brain connectivity and should be considered when generalizing results.
   - Some statements are overly strong, e.g., claiming that the classifier provides a "clear diagnosis boundary". Given the modest accuracy (73%), such claims should be tempered.

5. Ethical and Reporting Considerations
   - The paper mentions that all participants provided informed consent and lists funding sources. However, there is no description of ethics approval numbers or which institutional review boards approved the study.
   - Competing interests are declared but need to follow the standard BMC format.
   - Figures and tables are referenced but not included in the draft; ensure all supplementary materials are properly linked.
6. Specific Questions
   1. Why was the binarization threshold set at the top 20% of absolute FC values? Did you test other thresholds or weighted adjacency matrices?
   2. How did you handle head motion beyond Friston's 24-parameter model; were motion outliers or scrubbing applied?
   3. Were there any site differences in MRI acquisition parameters (e.g., scanner type, TR/TE)? How might these affect functional connectivity and generalization?
   4. The ComBat model includes covariates such as gender, age, and head motion. How did you verify that important biological variability was not removed by harmonization?
   5. How did you prevent data leakage when harmonizing the training and test sets in leave-one-site-out validation?
   6. Did you examine whether demographic variables (age, sex, education) differed across the identified subtypes?

7. What was the rationale for choosing a Graph Isomorphism Network (GIN) encoder over other GNN architectures? Did you try graph convolutional networks (GCN) or graph attention networks (GAT)?
8. The Concrete distribution requires a temperature parameter τ. How was this chosen, and how sensitive are results to τ?
9. How many parameters does EH-BrainGNN have compared to simpler models? Could overfitting contribute to the reported accuracy?
10. Accuracy differences between EH-BrainGNN and baselines are claimed to be significant. Were paired statistical tests performed across folds?
11. Did you use early stopping or any regularization techniques to prevent overfitting?
12. Ten-fold cross-validation within each site still mixes data from multiple sites. Did you test site-balanced folds?
13. How stable are the three subtypes across different initialization seeds of CFDP? Provide cluster assignment stability metrics.
14. Did you explore other clustering algorithms (e.g., k-means, spectral clustering) and compare results?
15. The largest subtype contains 62.1% of patients. Could this reflect residual site effects rather than distinct biology?
16. The p-values reported for HAMD item differences should be corrected for multiple comparisons. How many tests were performed and which correction method was used?
17. How was the FDR threshold chosen? Provide the q-values for each significant comparison.
18. Were symptom measures (HAMD, BDI) collected in both Chinese and Japanese cohorts? If not, how were differences handled?
19. Why did you choose Wilcoxon rank-sum tests instead of ANOVA or mixed models for symptom comparisons?
20. The GE-HAMD models use ridge regression with leave-one-out cross-validation. How do these models perform compared to linear regression or other machine-learning regressors?
21. Did you test whether the graph embeddings correlate with specific brain networks (e.g., default mode, salience)?
22. How does your work extend previous MDD subtyping frameworks beyond simply using GNN embeddings?
23. Are the learned subgraphs biologically plausible? For example, do they align with known depression circuitry?
24. How might these methods be applied to other psychiatric disorders or multimodal data (e.g., structural MRI)?
25. Did you evaluate whether medication status or comorbidities influenced classification or subtyping results?

26. Are there any ethical concerns in using AI-based diagnostic tools for psychiatric disorders? How would you address potential misclassification consequences?
27. What are the computational requirements for EH-BrainGNN? Could it be implemented in clinical settings?
28. Will you release the code and trained models? If not, how can other researchers reproduce your results?
29. How would you integrate EH-BrainGNN with existing clinical workflows (e.g., DSM-5 assessments)?
30. Given the moderate accuracy, what threshold would you consider acceptable for clinical deployment?

7. Recommendation

Given the significant issues in language, methodological transparency, statistical analysis, and interpretation, the manuscript is not yet suitable for publication in a journal . I recommend major revisions. The authors should address the grammatical errors, provide complete methodological details, justify their design choices, report comprehensive performance metrics with statistical tests, assess subtype stability, temper clinical claims, and make code and data readily available. Only after these substantial improvements should the work be reconsidered for peer review.
