# Supplementary material for: Functional connectivity–based classification and subtyping of major depression for precision mental health: An ensemble graph neural network approach
Source: PLOS Digit Health. 2026 Mar 4;5(3):e0001261. doi: 10.1371/journal.pdig.0001261 (PMC12959711; doi:10.1371/journal.pdig.0001261)
Supplement: Attachment — Submitted filename: Review Report and comments.pdf [file pdig.0001261.s016.pdf]

Review Report and comments

Manuscript ID: PDIG-D-25-00628

Title: Functional connectivity–based classification and subtyping of major depression for precision mental health: An ensemble graph neural network approach

Overall Assessment of the Revision

The authors have made a substantial and technically careful revision in response to prior reviewer and editor comments. Key concerns regarding data leakage, harmonization (ComBat), hyperparameter optimization, robustness analyses, and overly strong clinical claims have largely been addressed through additional analyses, clearer methodological descriptions, and more cautious framing.

The revision demonstrates serious engagement with reviewer feedback, including the addition of ablation studies, learning curves, clustering validation metrics, expanded Discussion, and public code availability. The manuscript is significantly improved compared with the original submission. However, several issues remain that prevent acceptance in its current form, primarily related to (i) abstract-level positioning, (ii) interpretability claims, (iii) clinical framing of subtypes, and (iv) limitations of subtyping validation.

Recommendation

Minor Revision

The remaining issues are conceptual and framing-related rather than technical, and they can be resolved without new experiments.

Major Issues Requiring Attention

1. Abstract still lacks adequate benchmarking and specificity

Abstract, page ~53, lines 33–41

The abstract reports a classification accuracy of approximately 73% and claims "reproducible subtypes," but still does not sufficiently:

- contextualize performance relative to existing FC-based or GNN-based approaches, or
- specify what "generalization" means in quantitative terms.

Although the authors have improved caution around clinical utility, the abstract remains too technical and insufficiently comparative for readers to immediately assess contribution.

Required action:

Add one concise sentence comparing performance to prior approaches and replace vague terms (e.g., “strong generalization”) with explicit metrics.

2. Interpretability claims remain largely qualitative

Discussion, page ~83–84, lines 15–40

The revised Discussion provides biologically plausible interpretations of subtype-specific circuits (DMN–cerebellum, insula–cingulum–temporal, frontostriatal) and reports stability metrics (bootstrap, Jaccard index). However:

- Interpretability is still presented narratively, without a quantitative comparison to known depression-related networks or atlases.
- Claims such as “self-explaining” remain insufficiently operationalized.

Required action:

Either (i) add a simple quantitative interpretability check (e.g., overlap with canonical resting-state networks), or

(ii) explicitly state that interpretability is exploratory and hypothesis-generating, not validated for clinical use.

3. Clinical implications are still borderline optimistic

Discussion, page ~84–85, lines 6–16

Despite improved caution, statements suggesting that subtype signatures may “inform personalized target selection for neuromodulation” still risk being interpreted as clinically actionable, particularly given the modest accuracy and lack of outcome data.

Required action:

Insert a clear boundary statement indicating that these implications should not guide clinical decision-making and require prospective interventional validation.

4. Subtyping validation remains internally constrained

Results (clustering), page ~52, lines 1–9

The authors have strengthened clustering justification using CFDP decision graphs, silhouette coefficients, and Dunn indices. However:

- Subtypes are validated only internally and across two datasets.
- No external replication beyond these cohorts is available.
-

Required action:
Explicitly emphasize that the number and structure of subtypes are method- and cohort-dependent, and that subtype solutions may vary with different features or clustering strategies.

Minor Issues

1. Statistical reporting emphasis
   Page ~83, lines 22–29
   Effect sizes should be emphasized more clearly relative to p-values, with explicit clarification that statistical significance does not imply clinical relevance.
2. Cultural and population differences
   Page ~87, lines 31–41
   The discussion of cultural and lifestyle differences between Chinese and Japanese cohorts is appropriate but brief. A short dedicated paragraph would strengthen transparency regarding generalizability.
3. Code availability details
   Page ~84, lines 26–29
   While the GitHub repository is provided, the manuscript should clarify whether trained model weights and fixed random seeds are included to ensure reproducibility.
4. Conclusion tone
   Page ~87, lines 26–30
   The conclusion remains slightly aspirational. Editors may prefer a limitation-focused closing sentence that reiterates what evidence is still missing.

Strengths of the Revision

- Clear correction of potential data leakage in ComBat harmonization
- Well-documented nested cross-validation and hyperparameter tuning
- Robustness analyses (architecture, loss ablation, learning curves)
- Improved statistical rigor and reporting transparency
- Public release of code and configuration files
- More cautious and responsible clinical framing than in the original version

Conclusion

This revised manuscript represents a careful, serious, and high-quality revision. The remaining concerns relate primarily to framing, interpretability claims, and clarity, not to methodological validity. With minor revisions addressing the points above, the manuscript would be suitable for publication.
