# Supplementary material for: Functional connectivity–based classification and subtyping of major depression for precision mental health: An ensemble graph neural network approach
Source: PLOS Digit Health. 2026 Mar 4;5(3):e0001261. doi: 10.1371/journal.pdig.0001261 (PMC12959711; doi:10.1371/journal.pdig.0001261)
Supplement: Attachment — Submitted filename: Manuscript Review.docx [file pdig.0001261.s013.docx]

**REVIEW OF FUNCTIONAL CONNECTIVITY–BASED CLASSIFICATION AND SUBTYPING OF MAJOR DEPRESSION FOR**

**PRECISION MENTAL HEALTH: AN ENSEMBLE GRAPH NEURAL NETWORK APPROACH**

**Abstract**

The study proposes a framework that applies graph neural networks and clustering to classify and subtype major depressive disorder using resting-state fMRI data. The approach was tested on two large datasets from China and Japan, achieving 73 percent accuracy and identifying three reproducible subtypes. The effort to move diagnosis toward biological markers is important, and the use of multi-site data with cross-country validation is a strength.

However, the abstract does not provide enough information to fully assess the contribution. The reported accuracy lacks comparison with existing methods, and the clinical relevance of the identified subtypes is not established. It is also unclear how scanner and site differences were addressed, and what “strong” generalization means in terms of performance metrics. The abstract is heavy on technical terms, which may limit accessibility. A relationship to clinical outcomes would improve the paper.

**Introduction**

The introduction highlights the limitations of current approaches to diagnosing and treating major depressive disorder. The authors point out that DSM-based definitions rely on subjective symptoms, which are heterogeneous and overlap with other disorders, limiting progress in treatment. They frame their work within the Research Domain Criteria initiative, emphasizing the need to redefine psychiatric disorders based on neural circuits and biological markers. Prior studies using functional connectivity and machine learning are discussed, and the challenges of poor generalization across datasets are noted. This sets up the rationale for the ensemble framework that combines supervised graph neural networks with unsupervised clustering, which the authors aim to validate across two large datasets.

While the introduction provides useful background, it is lengthy and somewhat repetitive. Several points about heterogeneity and generalization are made multiple times, and the description of the proposed framework is more detailed than what is typically expected in this section. Readers would benefit from a clearer focus on three elements: the problem of symptom-based diagnosis, the shortcomings of earlier neuroimaging classifiers, and the need for a more generalizable framework. Simplifying the language, and moving methodological details to later sections would imprve this section, make it aligned with public health readership.

**Methods**

The methods make strong use of large datasets and advanced graph-based models, but several design choices need deeper validation to ensure robustness. First, the binarization of functional connectivity by keeping only the top 20 percent of correlations is a strong modeling assumption that can heavily influence graph topology. The authors should test multiple thresholds or alternative weighting schemes (e.g., weighted adjacency matrices or sparsity-inducing regularization) to show that the results are not an artifact of this choice. Similarly, the reliance on Pearson correlation as the similarity metric could be complemented by partial correlations or other measures that better capture direct connectivity. Sensitivity analyses here would make the graph construction pipeline more defensible.

Second, while ComBat harmonization is appropriate for reducing site-related variability, the methods do not demonstrate that biological signal was preserved after adjustment. The authors should present diagnostics such as visualization of variance explained by site before and after harmonization, or checks that known case-control differences remain detectable. Without this, it is unclear whether harmonization might have inadvertently removed meaningful signal. The framework also integrates multiple loss components to balance sparsity, compactness, and predictive performance, but there is no discussion of how stable the results are under different weightings of these terms. A systematic ablation study of loss components and hyperparameter weighting would strengthen claims of robustness.

Finally, although the authors benchmark against a wide range of baselines, the evaluation strategy could be expanded. Reporting only average accuracy or AUC does not capture variability across folds, sites, or subgroups. Confidence intervals, variance across cross-validation folds, calibration measures, or subgroup-specific performance would provide a fuller picture of model reliability. The hyperparameter tuning strategy is also described only in broad terms; a more detailed account of the search space, convergence behavior, and variance across runs would increase confidence in reproducibility.

**Results**

The results show that EH-BrainGNN outperformed standard classifiers and baseline GNNs in both the principal and validation datasets, with accuracies around 74 percent. The leave-one-site-out analysis adds credibility by testing generalization, and the cross-national validation from China to Japan is an important strength. However, the interpretation of these results would be stronger if the authors provided confidence intervals or statistical tests directly comparing EH-BrainGNN to the baselines. Right now, the claim of “significant improvements” rests on visual inspection of figures and tables rather than formal tests. It is also unclear whether the improvements are consistent across all subgroups or driven by a few high-performing sites, as performance varied substantially (for example, 88 percent accuracy at site 3 vs 64 percent at site 17). More detail on why such site variability occurs, and whether it relates to sample size, demographics, or data quality, would increase confidence that the model is robust rather than opportunistically fitting specific datasets.

The t-SNE plots are used to argue that the learned graph embeddings capture diagnosis-specific structure, but this visualization alone is insufficient as evidence. The authors should complement t-SNE with quantitative measures such as cluster separability indices or supervised classification accuracy using embeddings alone. Similarly, while the subtyping analysis identifies three reproducible subgroups, the validity of these subtypes hinges on both statistical and clinical relevance. The paper shows some differences in HAMD and BDI scores, but effect sizes are small and not consistent across all comparisons. More importantly, the clustering method (CFDP) is not benchmarked against alternatives, leaving it unclear whether three is the optimal or most stable number of clusters. Without replication using a different clustering approach or statistical validation of cluster stability, the subtyping claims are less convincing.

The subtype-specific connectivity findings are interesting, particularly the consistent thalamic connections across subtypes. Still, these results need stronger statistical support. Many of the connections shown in Figure 6 could emerge by chance given the large number of comparisons, and it is not clear whether corrections for multiple testing were applied at the level of subgraph analysis. The link between connectivity patterns and specific symptoms, while suggestive, would also benefit from more rigorous modeling. For example, the ridge regression analysis reports r² values above 0.3, but it is unclear whether these results hold after correcting for potential confounders like age, sex, and motion. Reporting performance on held-out subgroups or applying permutation testing would make the symptom-association findings more convincing.

The validation analyses are a good addition, but they mostly confirm that choices like head motion regression, ROI atlas, and ComBat harmonization matter. What is missing is a sensitivity analysis quantifying how much each of these factors impacts performance. For instance, the switch from AAL to Power atlas improves results, but this is described only qualitatively. A systematic comparison, or at least reporting of effect sizes, would make the robustness checks more rigorous. Similarly, while harmonization improved some sites, the extent of improvement and its statistical significance are not fully shown. This makes it hard to assess whether the method is generally reliable or whether it still suffers from site-specific overfitting.

Overall, the result section is great, but can be better. To increase rigor, the authors should: (1) report confidence intervals and formal significance tests for performance comparisons, (2) provide quantitative validation of embedding discriminability and subtype stability, (3) apply multiple testing correction to connectivity results, and (4) quantify the effects of preprocessing choices in sensitivity analyses.

**Discussion**

The discussion highlights why the study is important, but some claims are stronger than the data allow. The cross-site and cross-country validation is a real strength, yet an accuracy of about 73 percent should be framed as encouraging but not sufficient for clinical application at this stage. A more cautious tone would make the contribution clearer without overstating its readiness.

The subtyping results are a valuable addition, especially given that three subtypes were consistently identified. Still, earlier studies have reported different numbers of subtypes depending on the method, which shows how unsettled this area remains. The authors should acknowledge that their findings may be influenced by the clustering approach and emphasize replication. Linking the subtypes more directly to treatment response or prognosis would also strengthen the case for clinical usefulness.

The limitations deserve more depth. The absence of treatment outcome data is important, but so is the possibility that results are shaped by preprocessing steps, site variability, or model hyperparameters. Expanding on these points would make the discussion more balanced and credible.

Thank you.
