# Supplementary material for: Functional connectivity–based classification and subtyping of major depression for precision mental health: An ensemble graph neural network approach
Source: PLOS Digit Health. 2026 Mar 4;5(3):e0001261. doi: 10.1371/journal.pdig.0001261 (PMC12959711; doi:10.1371/journal.pdig.0001261)
Supplement: Attachment — Submitted filename: Response Letters.docx [file pdig.0001261.s015.docx]

Summary of Changes -- manuscript PDIG-D-25-00628

Dear Chief Editor and Associate Editor,

The authors appreciate the insightful comments from the editors and reviewers for improving the quality of the manuscript greatly. In this submission, the manuscript has been revised in accordance with the referees’ comments and suggestions, with modified sentences or paragraphs highlighted with red in the revised manuscript. We sincerely hope that the revision would be satisfactory this time.

The point-to-point responses to editor’s and reviewers’ comments are listed below, where the referees’ comments and suggestions are shown in blue (italic font), followed by our responses in black.

Thanks again for your great efforts in reviewing the manuscript.

With kind regards,

Kai-Zhong Zheng

**Point-by-point responses to Editor:**

**Editor's comments:**

*Thank you for your submission. The study is promising and of broad interest, but as echoed across all reviews key aspects of methodological transparency, leakage control, interpretability, and robustness require clarification or additional analyses before the work can be considered further.

Please provide a point-by-point response to all reviewer comments and these editor items, and revise the manuscript accordingly.

1. ComBat usage and leakage control

* Explicitly state, for every evaluation setting (10-fold CV, leave-one-site-out, and independent validation), that ComBat parameters were estimated only on the training data within each outer fold/site and then applied to the held-out fold/site without re-fitting. For the cross-national test, fit on the Chinese training sites only and apply those parameters to the Japanese sites; confirm no parameters were estimated using any Japanese labels or distributions. Add a brief schematic of the preprocessing/CV pipeline that shows where ComBat is fit/applied. See Methods “Control of site differences and covariates” and Results sections describing CV/LOSO (e.g., pages 10–11 and 16–18), and make these procedures explicit.*
**Response:** We appreciate this important clarification request. In the revised manuscript, we have made the use of ComBat explicit for each evaluation setting and have added a schematic of the full preprocessing and cross validation pipeline.

First, we now clearly state in Methods (Control of site differences and covariates) and in the corresponding Results sections that, for all 10-fold cross validation experiments, ComBat parameters were estimated only on the training data within each outer fold. The learned ComBat harmonization parameters were then applied to the held-out fold, and no re fitting was performed using any information from the test fold.

Second, for the leave-one-site-out (LOSO) cross-validation experiments, we specify that ComBat was fitted exclusively on data from the training sites in each LOSO split. The estimated parameters were then applied to the left-out site, again without re-estimating ComBat on that site. No information from the held-out site, including its distributional characteristics, was used when fitting the harmonization model.

Third, for the independent cross-national validation, we now explicitly state that ComBat was fit only on the Chinese training sites and that the resulting parameters were subsequently applied to the Japanese sites. We confirm that no ComBat parameters were estimated using Japanese labels or Japanese distributional information, and that the Japanese cohort was treated strictly as an unseen target domain.

*2. Hyperparameter search protocol

* In “Training, testing and hyperparameter optimization,” specify the exact inner-loop search (e.g., inner K-fold within each outer fold), the grid explored (Table 2), and the selection criterion (e.g., mean AUC across inner folds). Confirm that hyperparameters and early-stopping criteria were fixed before scoring any outer test fold or the independent cohort. Provide code-level details needed to reproduce the search. Tie Table 2 to this description and report the final selected values per experiment.*
**Response:** We thank the reviewer for this constructive comment. In the revised manuscript, we have substantially expanded the subsection “Training, testing and hyperparameter optimization” to provide a precise and reproducible description of our model selection procedure.

Specifically, we now clarify that all hyperparameters were tuned using a nested cross-validation scheme. For each experiment, we performed an outer 10-fold cross-validation. Within each outer training split, we conducted an inner 5-fold cross-validation to optimize hyperparameters. For every candidate hyperparameter configuration defined in Table 2 (including learning rate, batch size, number of layers, number of hidden units, weight decay, dropout rate, and other model-specific parameters), the model was trained on 4 inner folds and evaluated on the remaining fold, cycling through all inner folds. We then selected the configuration that maximized the mean accuracy across the inner validation folds. After identifying the best hyperparameter configuration for a given outer fold, we retrained the model on the full outer training set using this fixed configuration. Early stopping was applied with a patience of 10 epochs, monitoring the validation accuracy computed on an internal validation split drawn exclusively from the outer training data. Importantly, neither the outer test folds nor the independent validation cohort were used at any stage for hyperparameter tuning, model selection, or calibration of early-stopping criteria. All hyperparameters and early-stopping rules were therefore fixed before scoring any outer test fold or the independent cohort, ensuring unbiased performance estimates.

We have also revised Table 2 to explicitly describe the hyperparameter grid explored in the inner-loop search and to report, for each experiment, the final selected values. In addition, we now provide code-level details sufficient to reproduce the search procedure and the corresponding implementation is provided in our public GitHub repository at https://github.com/ZKZ-Brain/EH-BrainGNN.

*3. Sensitivity analyses for deep model capacity and data size
* Report performance when varying the number of GNN layers and hidden dimensions (at minimum the values already listed in Table 2). Provide both cross-validation and independent-validation results for these variants. Add a learning-curve analysis showing accuracy/AUC as a function of training sample size to support the claim of robust representation learning.*

**Response:** We thank the reviewer for this important suggestion. In the revised manuscript, we have systematically evaluated the impact of GNN architecture on performance and explicitly report both cross validation and independent validation results for the requested variants.

First, we varied the number of GNN layers (2, 3, 4, 5) and the hidden dimensionality (64, 128, 256), covering at least the configurations listed in Table 2. For each combination, we report cross validation performance and independent validation performance in the cross cohort setting (training on the principal dataset and testing on the independent validation dataset). The full results are now summarized in Supplementary Figure 8 and 9. Across both datasets and both evaluation schemes, the configuration with 5 layers and a 128-dimensional hidden space consistently achieved the best or near best performance, while the other settings showed only moderate fluctuations in accuracy and AUC. These findings support the claim that our model performance is robust to reasonable variations in architectural depth and capacity, and that the specific configuration reported in the main text is a well-supported choice rather than an isolated optimum.

**Supplementary Figure S8. The impact of GNN layers and hidden dimensions on 10-fold cross-validation performance.**

**Supplementary Figure S9. The impact of GNN layers and hidden dimensions on generalization performance in the independent validation dataset using the principal dataset.**

Second, as suggested, we have added a learning curve analysis in which we vary the size of the training set and plot test accuracy as a function of training sample size (Supplementary Figure 10). The results show a monotonic increase in test accuracy as the number of training subjects grows, with no evidence of abrupt degradation at larger sample sizes. This result indicates that the model continues to benefit from additional data and that the learned representations generalize increasingly well rather than overfitting to small training subsets. We now include this analysis in the Results to support our statement about robust representation learning.

**Supplementary Figure S10. Learning curve analysis.**

*4. Graph construction threshold (top 20%)
* Justify the 20% binarization choice with citations; if empirical, report an ablation across thresholds (e.g.,) and summarize the impact on performance and subtype discovery. Consider treating the threshold as a tunable parameter in inner CV and report whether conclusions are stable.*
**Response:** We thank the reviewer for raising this important point. In the revised manuscript, we now explicitly justify our choice of a 20% binarization threshold and assess its impact on both predictive performance and subtype discovery.

First, we note that percentile-based binarization in the range of 10–30% has been commonly adopted in related work to balance sparsity with sufficient signal retention in high-dimensional biological data, and we now cite these studies in the Methods section (refs. [1-3]). Motivated by this practice, we originally selected 20% as a moderate cutoff that avoids overly sparse representations while still emphasizing the most informative features. To empirically evaluate this choice, we performed an ablation study over five candidate thresholds (10%, 15%, 20%, 25% and 30%) under all three evaluation schemes used in this work: (i) 10-fold cross-validation, (ii) leave-one-site-out cross-validation, and (iii) training on the principal dataset with testing on the independent validation cohort. The results are summarized in Supplementary Figure 7 and Supplementary Tables 5–6. Across all three schemes, the 20% threshold consistently achieved the highest or near-highest mean AUC, with only modest fluctuations in performance at neighboring thresholds.

**Supplementary Figure 7 The impact of graph construction threshold on 10-fold cross-validation.**

**Supplementary Table 5 The impact of graph construction threshold on leave-one-site cross-validation.**

| **Threshold** | **10%** | **15%** | **20%** | **25%** | **30%** |
| --- | --- | --- | --- | --- | --- |
| **Site1** | 0.56 | 0.55 | **0.63** | **0.63** | **0.63** |
| **Site2** | 0.73 | 0.70 | **0.80** | 0.67 | 0.67 |
| **Site3** | 0.80 | 0.73 | **0.88** | 0.71 | 0.71 |
| **Site4** | 0.72 | 0.71 | **0.75** | 0.69 | 0.68 |
| **Site5** | 0.67 | 0.68 | **0.70** | 0.64 | 0.67 |
| **Site6** | 0.64 | 0.63 | **0.69** | 0.68 | 0.63 |
| **Site7** | 0.72 | 0.66 | **0.75** | 0.65 | 0.68 |
| **Site8** | **0.73** | **0.73** | 0.70 | 0.65 | 0.62 |
| **Site9** | **0.78** | 0.75 | **0.78** | **0.78** | **0.78** |
| **Site10** | 0.68 | 0.69 | **0.72** | 0.71 | 0.67 |
| **Site11** | 0.82 | 0.81 | 0.84 | **0.85** | 0.84 |
| **Site12** | 0.62 | 0.61 | **0.67** | 0.61 | 0.65 |
| **Site13** | 0.65 | 0.69 | **0.73** | 0.69 | 0.65 |
| **Site14** | 0.60 | 0.59 | **0.63** | 0.59 | 0.60 |
| **Site15** | 0.55 | 0.59 | **0.68** | 0.59 | 0.61 |
| **Site16** | 0.61 | 0.53 | **0.76** | 0.59 | 0.63 |
| **Site17** | 0.64 | 0.64 | 0.64 | **0.73** | **0.73** |
| **Mean** | 0.68 | 0.66 | **0.73** | 0.67 | 0.67 |

**Supplementary Table 6 The impact of graph construction threshold on generalization performance in the independent validation dataset using the principal dataset.**

| **Threshold** | **10%** | **15%** | **20%** | **25%** | **30%** |
| --- | --- | --- | --- | --- | --- |
| **HUH** | 0.65 | 0.65 | **0.73** | 0.66 | 0.63 |
| **HRC** | 0.72 | 0.74 | 0.74 | **0.75** | **0.75** |
| **HKH** | 0.63 | 0.60 | **0.66** | 0.63 | **0.66** |
| **COI** | 0.64 | 0.62 | **0.66** | 0.65 | 0.60 |
| **Mean** | 0.66 | 0.65 | **0.70** | 0.67 | 0.66 |

We further evaluated the influence of the binarization threshold on subtype discovery. Although individual sample assignments exhibited minor perturbations at the lowest and highest thresholds, the overall subtype architecture and the defining biological signatures of each subtype were conserved across the 10–30% range. In this context, the 20% threshold yielded a favorable compromise, providing highly stable subtype solutions with clear inter-subtype separation and consistently strong predictive performance.

[1] CI-GNN: A Granger Causality-Inspired Graph Neural Network for Interpretable Brain Network-Based Psychiatric Diagnosis. Neural Networks, 2024.

[2] BrainIB: Interpretable Brain Network-Based Psychiatric Diagnosis With Graph Information Bottleneck. IEEE Transactions on Neural Networks and Learning Systems, 2025.

[3] BPI-GNN: Interpretable brain network-based psychiatric diagnosis and subtyping. Neuroimage, 2024.

*5. From embeddings to edges: interpretability path
* Figures 5–6 show subtype-associated edges but the derivation from learned embeddings is not sufficiently documented. Expand Methods to detail how edges are selected from the explanation mask/embeddings, including any statistics, thresholds, or aggregation across subjects. Discuss interpretability in the context of biomarker development.*

**Response:** Thank you very much for this valuable suggestion. In the revised manuscript, we have expanded the Methods section to provide a detailed description of how edges are selected from the explanation masks and embeddings. We have also revised the Discussion to further elaborate on the implications of the identified biomarkers for clinical translation and potential deployment.

**1. From embeddings to edge-level interpretability**

To link the learned embeddings to interpretable, edge-level connectivity patterns, we implemented a three-step procedure.

- **Computation of subject-level explanation masks**

For each subject $s$, we consider the input graph $G_{s}=(V_{s},E_{s})$ with functional connectivity edges $e\in E_{s}$. Using the **subgraph generator module** of our framework, we obtain an edge-wise explanation mask for subject $s$, that assigns to each edge a non-negative importance score

$m_{s}\left( e \right)\mathbb{\in R\geq}0, e\in E_{s}$,

which quantifies how strongly edge $e$contributes to the model’s prediction for that subject.

- **Subject-level edge selection (top-20 edges)**

Within each subject, we rank all edges in descending order of their explanation scores $m_{s}\left( e \right)$. Let $\left\{ e_{s,\left( 1 \right)},\ldots,e_{s,\left( K \right)} \right\}$ denote the $K$ edges with the largest mask values for subject $s$, i.e.

$$m_{s}\left( e_{s,\left( 1 \right)} \right)\geq m_{s}\left( e_{s,\left( 2 \right)} \right)\geq\ldots\geq m_{s}\left( e_{s,\left( K \right)} \right),$$

with $K=20$ in all experiments. We then define the subject-level top-$K$ edge set as

$$T_{s}=\left\{ e_{s,\left( 1 \right)},\ldots,e_{s,\left( K \right)} \right\}\subseteq E_{s},$$

which provides a sparse, subject-specific summary of the most influential connections.

- **Aggregation across subjects within each subtype**

Let $c\in\left\{ 1,\ldots,C \right\}$ index the CFDP-derived subtypes, and

$$S_{c}=\left\{ s:\text{subject} s \text{is assigned to subtype} c \right\},$$

denote the set of subjects in subtype $c$. For each edge $e$in the common edge index space, we compute a selection frequency

$$f_{c}\left( e \right)=\frac{1}{\left| S_{c} \right|}\sum_{s\in S_{c}} I\left[ e\in T_{s} \right],$$

where $I\left[ \cdot\right]$ denotes the indicator function, which equals 1 if the condition is satisfied and 0 otherwise.

**2. The implications of the identified biomarkers for clinical translation and potential deployment**

We have expanded the Discussion to clarify how the subtype-specific connectivity patterns identified by our framework can be viewed as candidate circuit-level biomarkers with potential implications for precision treatment in MDD.

First, our clustering of learned embeddings revealed three reproducible MDD subtypes characterized by distinct connectivity motifs: (i) a default mode network–cerebellum subtype, (ii) an insula–cingulum–temporal subtype, and (iii) a frontostriatal subtype. Because our edge-level explanations are derived directly from the predictive model, these subtype-specific patterns provide an interpretable link between latent representations and biologically meaningful circuits, rather than abstract, model-internal features.

Second, we now discuss how these circuit signatures may inform personalized target selection for neuromodulation, particularly in the context of rTMS, where current practice in MDD largely relies on “one-size-fits-all” dorsolateral prefrontal cortex (DLPFC) targeting and lacks principled strategies for individualized target choice. Our results suggest a testable precision-medicine framework: patients assigned to the default mode network–cerebellum subtype might benefit from cerebellar stimulation as a candidate target; those in the insula–cingulum–temporal subtype might be preferentially considered for insula- or salience-network–oriented targeting; and those in the frontostriatal subtype align more closely with conventional DLPFC-based stimulation protocols. We explicitly note in the revised Discussion that these implications are hypothesis-generating rather than prescriptive, and that rigorous prospective interventional trials would be required to determine whether subtype-guided rTMS targeting indeed improves treatment response.

Finally, we emphasize that the edge-level biomarkers identified by our explainable framework are (i) sparse, (ii) circuit-specific, and (iii) reproducible across resampling and permutation tests, properties that are desirable for translational biomarkers intended for clinical deployment. We have added text to highlight how this combination of interpretability and robustness moves beyond purely predictive models and points towards a mechanistically grounded, subtype-informed approach to biomarker development in depression.

*6. Stability and significance of subtype-specific patterns
* Quantify the stability of CFDP subtypes and the “top 20 edges” using resampling/bootstrapping (e.g., adjusted Rand index/NMI across resamples, edge selection frequencies, and permutation tests with multiple-comparison control). Report effect sizes and confidence intervals where appropriate.*

**Response:** Thank you for this important suggestion. In the revised manuscript, we have added a systematic stability analysis for both the CFDP-derived subtypes and the subtype-specific “top 20 edges”.

- **Stability of CFDP subtypes**

We performed a bootstrap resampling procedure with 1,000 resamples. For each bootstrap sample of subjects (sampled with replacement), we re-ran the CFDP pipeline on the learned embeddings using the same hyperparameters and obtained a new clustering assignment. We then quantified the agreement between the original clustering and each bootstrap clustering using the adjusted Rand index (ARI) and normalized mutual information (NMI), and summarized the distribution of these values by reporting the mean, standard deviation, and 95% bootstrap confidence intervals. The resulting stability was substantial (ARI: mean = 0.523, 95% CI [0.136, 0.863]; NMI: mean = 0.543, 95% CI [0.198, 0.809]).

To determine whether this stability exceeds what would be expected by chance, we additionally generated a null distribution by repeating the same procedure after permuting the embeddings or labels. Comparisons between the observed and null distributions yielded large effect sizes (Cohen’s d for ARI = 3.13; Cohen’s d for NMI = 4.38), indicating that the CFDP-derived subtypes are markedly more reproducible than random clusterings. These results are reported in the Methods and Results section.

- **Stability of subtype-specific “top 20 edges”**

To quantify the robustness of the subtype-associated edges, we performed 1000 bootstrap resampling of subjects. For each resample, we recomputed the subject-level top-20 edges and aggregated them within each subtype to obtain resampled subtype-level top-20 sets, which were then compared with the reference set using the Jaccard index. The corresponding results are shown in Supplementary Figure S4.

As shown in Supplementary Fig. S4 A, the subtype-specific edge sets exhibited moderate-to-high stability across resamples: the mean Jaccard overlap was 0.433 (95% CI [0.250, 0.600]) for subtype 1, 0.405 (95% CI [0.176, 0.667]) for subtype 2, and 0.335 (95% CI [0.143, 0.538]) for subtype 3. Because both the reference and bootstrap-derived sets contain 20 edges, these values correspond to approximately 10–12 shared edges on average, indicating that more than half of the reported edges are consistently recovered across resamples, while the remaining edges form a more variable peripheral set. Notably, these overlaps are far above chance, as random selection of 20 edges from the full edge set yields Jaccard values close to zero, supporting the presence of a reproducible core of subtype-specific connections. Moreover, for each subtype, a substantial fraction of the reference top-20 edges showed high bootstrap selection frequencies (>0.5), with 19 of 20 edges in subtype 1, 16 of 20 in subtype 2, and 9 of 20 in subtype 3 exceeding this threshold, further highlighting a robust core set of connections (Supplementary Fig. S4 B–D).

**Supplementary Figure S4. The stability of the subtype-specific top-20 edges.** (A) the stability of the subtype-specific top-20 edges across bootstrap resamples quantified by the Jaccard index. (B) the bootstrap selection frequency of the reference top-20 edges for each subtype.

- **Permutation tests and multiple-comparison control**

To assess whether the observed edge selection frequencies and CFDP-derived subtypes could be explained by random variation, we performed a non-parametric, label-permutation test. Specifically, in each of 1,000 iterations we randomly permuted subtype labels across subjects and then repeated the full analysis pipeline under the permuted labels: we re-aggregated the explanation masks and reapplied the edge-selection procedure to construct a null distribution of selection frequencies for each edge, and we re-ran the CFDP clustering pipeline to obtain null distributions for the clustering stability metrics including ARI and NMI.

From these null distributions, we derived empirical *p*-values and corrected them for multiple comparisons using the Benjamini–Hochberg FDR procedure. Across the 1,000 permutations, empirical p-values for both the subtype-specific top-20 edges and the CFDP clustering stability metrics were consistently < 0.0001, indicating that the observed edge selection patterns and subtype structure deviate markedly from what would be expected under a random-label null model, and supporting the presence of a genuine and robust underlying biological signal.

*7. CFDP decision rules and K selection
* In “Module D: Subtype generator,” report the explicit rules or thresholds used on the CFDP decision graph to choose cluster centers and the number of clusters. Provide the decision graph(s) and show why K=3 is selected in both datasets, with a cluster-quality metric (e.g., silhouette, Dunn, or density-based criteria).*
**Response:** We thank the reviewer for this helpful suggestion. In the revised manuscript, we have substantially clarified how cluster centers and the number of clusters are determined and have added both visual and quantitative evidence supporting the choice of $K=3$ in both datasets.

First, we now explicitly describe the rules used with CFDP (clustering by fast search and find of density peaks) to select cluster centers from the decision graph. For each subject, CFDP computes a local density $\rho_{i}$ and a distance to the nearest point with higher density $\delta_{i}$. We plot $\delta_{i}$ against $\rho_{i}$ to obtain the decision graph and define a combined prominence score:

$$\gamma_{i}=\rho_{i}\times\delta_{i}.$$

Points with large values of both $\rho_{i}$ and $\delta_{i}$ correspond to high $\gamma_{i}$ and are treated as candidate cluster centers. For each dataset, we visualize the decision graph, identify the prominent corner region with high density and large distance, and select as centers those points with the largest $\gamma_{i}$. As shown in the decision graphs (Figure 5 A and C) and corresponding spatial distributions (Figure 5 B and D) that we now include for both datasets, three such prominent peaks are clearly present, leading to three CFDP centers in each case.

Second, in accordance with the reviewer’s request, we have added cluster quality analyses across a range of candidate values of $K$. Using the learned embeddings and the corresponding CFDP assignments, we compute the silhouette coefficient and Dunn index for different choices of $K$. In both datasets, $K=3$ either attains the highest silhouette coefficient or lies very close to the optimum, and it is also associated with higher Dunn values compared with neighboring values of $K$ (Figure 5 E-H). These results are now reported in the Results and illustrated in Figure 5 E-H that shows the silhouette and Dunn indices as functions of $K$, where three clusters emerge as the most parsimonious and well supported solution.

**Figure 5. Determination of the optimal number of CFDP subtypes.** (A-B) CFDP decision graphs for the two datasets. (C-D) Two dimensional embeddings illustrating the spatial distribution of subjects colored by CFDP subtype. (E-F) Silhouette coefficients as a function of the number of clusters ($K=2-9$). (G-H) Dunn indices for $K=2-9$.

*8. Clinical covariates and medication
* REST-meta-MDD contains clinical variables (e.g., medication). Clarify availability and whether medication status (and other clinical covariates) affect subtyping. Provide sensitivity analyses controlling for medication (and, if feasible, analyses restricted to medication-naïve participants), or clearly justify if unavailable.*

**Response:** We appreciate the reviewer’s insightful comment regarding the potential influence of clinical covariates, particularly medication status, on the identified subtypes. In the revised manuscript, we have added additional sensitivity analyses addressing these concerns.

To test whether medication status affects the subtyping solution, we performed a sensitivity analysis in which medication was treated as a covariate. Specifically, we regressed out medication status from the graph embeddings and repeated the subtyping procedure. As shown in Supplementary Figure S11 A–B, the analysis again yielded three subtypes, and subtype assignments were highly stable: fewer than 2% of participants changed subtype relative to the original solution.

Additionally, we conducted an additional analysis restricted to medication-naïve participants and repeated the subtyping. As shown in Supplementary Figure S11 C–D, this restricted sample likewise produced three subtypes, with subtype assignments differing from the full-sample solution for fewer than 2% of participants.

**Supplementary Figure S11 The impact of medication status on subtype analysis. (A-B)** CFDP decision graph and spatial distribution of subtypes with medication included as a covariate. **(C-D)** CFDP decision graph and spatial distribution of subtypes with medication-naïve participants. *9. Symptom prediction statistics
* In “Correspondences between functional signature and MDD symptoms,” report significance testing for r² values (e.g., permutation-based P values with FDR control), 95% CIs, and add the requested scatter plots of predicted vs observed scores. Clarify that “leave-one-out ridge regression” is leave-one-subject-out CV (not leave-one-site-out). Report the exact number of PCA components retained (not only “95% variance”).*

## Response: We thank the reviewer for these very helpful suggestions. We have revised the manuscript accordingly:

## 1. Significance testing for $\boldsymbol{R^{2}}$

## We now report permutation-based significance testing for all cross-validated $\boldsymbol{R^{2}}$ values. Specifically, for each predictive model we generated 1,000 null $\boldsymbol{R^{2}}$ values by repeating the entire analysis pipeline with randomly permuted symptom scores while keeping the functional features and model parameters identical. The observed $\boldsymbol{R^{2}}$ was then compared with this empirical null distribution to obtain a permutation-based *p* value, and multiple comparisons across symptom dimensions were controlled using false discovery rate (FDR) correction. The resulting permutation-based p values, together with the 95% confidence intervals (CIs) around the observed r², are summarized in Table 6.

## 2. Additional performance metric (MAE)

## To further characterize predictive performance, we have also added the mean absolute error (MAE) as a complementary metric to $\boldsymbol{R^{2}}$. MAE values are now reported in the updated table 6.

## Table 6. Subtype-specific clinical profiles for significantly different depression symptoms. The highest performance is highlighted with boldface.

| **Symptoms** | **Subtype1** | | | | **Subtype2** | | | | **Subtype3** | | | |
| --- | --- | --- | --- | --- | --- | --- | --- | --- | --- | --- | --- | --- |
|  | $\boldsymbol{R}^{\boldsymbol{2}}$ | **95% CI** | **MAE** | ***p* values** | $\boldsymbol{R}^{\boldsymbol{2}}$ | **95% CI** | **MAE** | ***p* values** | $\boldsymbol{R}^{\boldsymbol{2}}$ | **95% CI** | **MAE** | ***p* values** |
| Depressed Mood | 0.48 | [0.34, 0.60] | 0.55 | <${10}^{-4}$ | 0.34 | [0.25, 0.43] | 0.65 | <${10}^{-4}$ | **0.56** | [0.42, 0.71] | **0.39** | <${10}^{-4}$ |
| Insomnia early | 0.46 | [0.33, 0.55] | 0.46 | <${10}^{-4}$ | 0.37 | [0.28, 0.43] | 0.53 | <${10}^{-4}$ | **0.56** | [0.28, 0.72] | **0.33** | <${10}^{-4}$ |
| Insomnia middle | 0.48 | [0.35, 0.58] | 0.42 | <${10}^{-4}$ | 0.35 | [0.26, 0.42] | 0.47 | <${10}^{-4}$ | **0.54** | [0.30, 0.73] | **0.40** | <${10}^{-4}$ |
| Agitation | 0.45 | [0.30, 0.54] | **0.47** | <${10}^{-4}$ | 0.42 | [0.31, 0.51] | 0.52 | <${10}^{-4}$ | **0.51** | [0.31, 0.70] | **0.47** | <${10}^{-4}$ |
| Genital | 0.53 | [0.43, 0.61] | 0.43 | <${10}^{-4}$ | 0.44 | [0.37, 0.52] | 0.47 | <${10}^{-4}$ | **0.57** | [0.41, 0.68] | **0.40** | <${10}^{-4}$ |
| Weight Loss | 0.40 | [0.29, 0.48] | **0.47** | <${10}^{-4}$ | 0.34 | [0.25, 0.42] | 0.48 | <${10}^{-4}$ | **0.57** | [0.43, 0.68] | **0.47** | <${10}^{-4}$ |
| Insight | 0.44 | [0.31, 0.59] | 0.37 | <${10}^{-4}$ | 0.36 | [0.29, 0.43] | 0.39 | <${10}^{-4}$ | **0.56** | [0.43, 0.63] | **0.34** | <${10}^{-4}$ |

## 3. Scatter plots of predicted vs observed scores

As requested, we have added scatter plots showing predicted versus observed symptom scores (Supplementary Figure S5).

**Supplementary Figure S5. The scatter plots of predicted vs observed HAMD scores.**

## 4. Clarification of “leave-one-out ridge regression”

We now explicitly state that “leave-one-out ridge regression” refers to leave-one-subject-out cross-validation.

## 5. Exact number of PCA components retained

In addition to stating that we retained components explaining 95% of the variance, we now specify that nine principal components were used in the analyses, jointly accounting for around 95% of the total variance in the feature space.

*10. Statistical reporting and comparators
* Where you mark superiority (e.g., Fig. 3A–B), specify the statistical test, correction for multiple comparisons, and report exact P values or adjusted CIs. Provide 95% CIs for key metrics (ACC/AUC), site-wise confusion matrices or balanced accuracy where class imbalance exists, and a brief calibration summary (e.g., Brier score or reliability plot) for the classifier.*

**Response:** We thank the reviewer for this constructive request. We have revised the manuscript to make all superiority markings statistically explicit and reproducible, and we now report the statistical test, multiple-comparison handling, and exact P values (or adjusted CIs), together with uncertainty, class-imbalance–aware metrics, and calibration summaries.

- **Statistical test and superiority markings (Fig. 3A–B)**

Where superiority is highlighted, we compare EH-BrainGNN against the next-best method using paired tests across the 10 cross-validation folds (fold-wise ACC as paired observations). For the Principal Dataset, EH-BrainGNN is compared to BrainIB in terms of ACC: *t* = 4.3529, *p* = 0.0018, Cohen’s *d* = 1.3765 with bootstrap 95% CI [0.7998, 2.9158]. For the Independent validation dataset, EH-BrainGNN is compared to CI-GNN (ACC): *t*= 3.1623, *p* = 0.0115, Cohen’s d = 1.0000 with bootstrap 95% CI [0.4454, 2.4244].

- **Correction for multiple comparisons**

Because each superiority marker corresponds to a pre-specified single comparison against the next-best baseline within the relevant setting, the primary inference is not inflated by testing many baselines simultaneously. For completeness, we also provide BH-FDR–adjusted q-values across the set of superiority tests reported in Fig. 3A–B (two tests): *q* = 0.003685 (Principal) and *q* = 0.011508 (Independent).

- **Class imbalance and site-wise reporting**

To address class imbalance and site heterogeneity, we now report sensitivity and F1-score alongside ACC and AUC for the generalization evaluation on the independent validation dataset (trained on the principal dataset), providing a more informative assessment under potential class imbalance.

**Table 5. Generalization performance on different models in the independent validation dataset using the principal dataset.** The highest performance is highlighted with boldface.

| Sites  Method | HUH | | | | HRC | | | | HKH | | | | COI | | | |
| --- | --- | --- | --- | --- | --- | --- | --- | --- | --- | --- | --- | --- | --- | --- | --- | --- |
|  | ACC | AUC | Sen | F1 | ACC | AUC | Sen | F1 | ACC | AUC | Sen | F1 | ACC | AUC | Sen | F1 |
| LASSO | 0.59 | 0.60 | 0.51 | 0.58 | 0.43 | 0.58 | 0.27 | 0.41 | 0.50 | 0.50 | 0.48 | 0.48 | 0.57 | 0.56 | 0.66 | 0.66 |
| RF | 0.47 | 0.54 | 0.41 | 0.47 | 0.34 | 0.46 | 0.16 | 0.27 | 0.61 | 0.61 | 0.55 | 0.58 | 0.48 | 0.56 | 0.41 | 0.50 |
| L-SVM | 0.58 | 0.59 | 0.74 | 0.67 | 0.49 | 0.53 | 0.39 | 0.52 | 0.48 | 0.49 | 0.24 | 0.30 | 0.56 | 0.59 | 0.65 | 0.65 |
| RBF-SVM | 0.61 | 0.61 | 0.59 | 0.62 | 0.42 | 0.55 | 0.35 | 0.47 | 0.45 | 0.46 | 0.21 | 0.27 | 0.63 | **0.63** | 0.67 | 0.71 |
| GCN | 0.67 | 0.68 | 0.69 | 0.70 | 0.62 | 0.52 | 0.69 | 0.72 | 0.57 | 0.58 | 0.52 | 0.55 | 0.56 | 0.53 | 0.62 | 0.64 |
| GAT | 0.66 | 0.67 | 0.59 | 0.66 | 0.60 | 0.58 | 0.65 | 0.71 | 0.61 | 0.61 | 0.34 | 0.45 | 0.63 | 0.58 | 0.70 | 0.71 |
| GIN | 0.45 | 0.48 | 0.03 | 0.05 | 0.61 | 0.61 | 0.59 | 0.70 | 0.53 | 0.53 | 0.66 | 0.55 | 0.55 | 0.52 | 0.62 | 0.64 |
| DIR-GNN | 0.66 | 0.67 | 0.75 | 0.71 | 0.61 | 0.61 | 0.59 | 0.68 | 0.62 | 0.64 | 0.55 | 0.59 | 0.59 | 0.59 | 0.56 | 0.62 |
| ProtGNN | 0.62 | 0.62 | 0.56 | 0.62 | 0.61 | 0.59 | 0.65 | 0.72 | 0.61 | 0.61 | 0.55 | 0.58 | 0.53 | 0.55 | 0.49 | 0.57 |
| BrainGNN | 0.58 | 0.59 | 0.63 | 0.63 | 0.68 | 0.57 | 0.78 | 0.76 | 0.52 | 0.53 | 0.48 | 0.49 | 0.61 | 0.50 | 0.64 | 0.67 |
| CI-GNN | 0.61 | 0.61 | 0.62 | 0.63 | 0.69 | 0.58 | 0.78 | 0.77 | 0.65 | 0.65 | 0.62 | 0.62 | 0.58 | 0.57 | 0.58 | 0.63 |
| BrainIB | 0.52 | 0.71 | 0.39 | 0.64 | 0.53 | 0.63 | 0.59 | 0.70 | 0.53 | 0.52 | 0.52 | 0.52 | 0.63 | 0.58 | **0.86** | **0.74** |
| EH-BrainGNN | **0.73** | **0.72** | **0.79** | **0.76** | **0.74** | **0.62** | **0.86** | **0.83** | **0.66** | **0.67** | **0.72** | **0.67** | **0.66** | **0.63** | 0.75 | **0.74** |

- **Calibration**

In addition, we added calibration diagnostics to provide a fuller picture of model reliability, reporting calibration metrics (e.g., ECE). Calibration was quantified using the 10-bin expected calibration error (ECE), which was 0.030 after Platt scaling. Platt scaling was fitted within each outer training fold using an internal calibration split and then applied to the corresponding outer test fold (or held-out site); neither the outer test fold nor the independent validation cohort was used to fit the calibrator, preventing information leakage. This indicates that our predictions are well calibrated and supports the reliability of our results.

*11. Reproducibility resources

* Provide a public repository with full code, random seeds, environment details, and scripts to reproduce preprocessing, ComBat fitting/applying within CV, model training, and all analyses/figures. Include model weights or instructions to retrain.*
**Response:** We thank the reviewer for this important request and agree that full reproducibility requires more than high-level methodological description. We have therefore created a public GitHub repository that includes the complete implementation and all materials needed to reproduce our experiments: https://github.com/ZKZ-Brain/EH-BrainGNN.

*12. Overall framework exposition
* Rewrite “Overall framework of EH-BrainGNN” to first present representation learning for classification, then the downstream subtyping on learned features. Add a schematic of the full network with parameter counts and a clear statement of the role of the subgraph/explainer (see Fig. 2 on page 25 and related text).*

**Response:** We thank the reviewer for pointing this out. We have thoroughly rewritten the “Overall framework of EH-BrainGNN” section to improve readability and to better reflect the learning pipeline. Specifically, we now first describe feature representation learning driven by the supervised classification objective, including how the graph encoder produces node- and graph-level embeddings, and then present the downstream subtyping procedure that operates on the learned graph-level features (without affecting the classifier training). To further improve clarity, we have added a schematic diagram of the full deep network architecture with the number of trainable parameters for each module and in total (see Figure 3). Parameter counts are computed as the total number of trainable weights and biases in each learnable layer; parameter-free operations (e.g., ReLU, dropout, pooling/flattening) are counted as zero.

In addition, we have clarified the role of the subgraph generator. The subgraph module is integrated into the representation learning stage as an explainer/regularizer that samples discriminative subgraphs and is jointly optimized with the graph encoder. A mutual-information-based constraint is used to encourage the sampled subgraph to retain informative content from the original graph. We have added this explanation in the revised text and the schematic accordingly (Figure 3B).

**Figure 3 A schematic of the full network with parameter counts (A) and a clear statement of the role of the subgraph generator (B).**

*13. Figures and captions
* Figure 3C vs text: ensure consistency between “ranking” and “accuracy,” or present both in a harmonized way.
* Figure 5D: thicken lines/edges and improve visibility (Reviewer 2).*

**Response:** Corrected as suggested.

** Add the requested scatter plots for symptom prediction.*

**Response:** As requested, we have added scatter plots showing predicted versus observed symptom scores (Supplementary Figure S5).

**Supplementary Figure S5. The scatter plots of predicted vs observed HAMD scores.**

** Correct any caption mismatches (e.g., the caption noted by Reviewer 2 around lines 505–507) and typographical slips in Results (e.g., “subtyp2s”). Indicate per-subtype sample sizes alongside percentages.*

**Response:** Corrected as suggested.

**Point-by-point responses to Reviewers:**

**Reviewer 's comments:**

*Reviewer #1:*

*1.Please clarify the scope of ComBat's application. For instance, in lines 280-281, the sentence “Furthermore, we used ComBat harmonization method to mitigate distinct site differences and artifact or confounding effect” should specify whether ComBat was fitted on each outer training fold and then applied to that fold's test set.*

**Response:** We appreciate this important clarification request. In the revised manuscript, we have made the use of ComBat explicit for each evaluation setting and have added a schematic of the full preprocessing and cross validation pipeline.

First, we now clearly state in Methods (Control of site differences and covariates) and in the corresponding Results sections that, for all 10-fold cross validation experiments, ComBat parameters were estimated only on the training data within each outer fold. The learned ComBat harmonization parameters were then applied to the held-out fold, and no re fitting was performed using any information from the test fold.

Second, for the leave-one-site-out (LOSO) cross-validation experiments, we specify that ComBat was fitted exclusively on data from the training sites in each LOSO split. The estimated parameters were then applied to the left-out site, again without re-estimating ComBat on that site. No information from the held-out site, including its distributional characteristics, was used when fitting the harmonization model.

Third, for the independent cross-national validation, we now explicitly state that ComBat was fit only on the Chinese training sites and that the resulting parameters were subsequently applied to the Japanese sites. We confirm that no ComBat parameters were estimated using Japanese labels or Japanese distributional information, and that the Japanese cohort was treated strictly as an unseen target domain.

*2.The section “Training, testing and hyperparameter optimization” did not specify the grid search process or the basis for hyper-parameter selection. For instance, was the grid search conducted on the inner layers of training set in each outer fold? Was the selection criterion based on maximizing the average AUC of the inner layers?*

**Response:** We thank the reviewer for this constructive comment. In the revised manuscript, we have substantially expanded the subsection “Training, testing and hyperparameter optimization” to provide a precise and reproducible description of our model selection procedure.

Specifically, we now clarify that all hyperparameters were tuned using a nested cross-validation scheme. For each experiment, we performed an outer 10-fold cross-validation. Within each outer training split, we conducted an inner 5-fold cross-validation to optimize hyperparameters. For every candidate hyperparameter configuration defined in Table 2 (including learning rate, batch size, number of layers, number of hidden units, weight decay, dropout rate, and other model-specific parameters), the model was trained on 4 inner folds and evaluated on the remaining fold, cycling through all inner folds. We then selected the configuration that maximized the mean accuracy across the inner validation folds. After identifying the best hyperparameter configuration for a given outer fold, we retrained the model on the full outer training set using this fixed configuration. Early stopping was applied with a patience of 10 epochs, monitoring the validation accuracy computed on an internal validation split drawn exclusively from the outer training data. Importantly, neither the outer test folds nor the independent validation cohort were used at any stage for hyperparameter tuning, model selection, or calibration of early-stopping criteria. All hyperparameters and early-stopping rules were therefore fixed before scoring any outer test fold or the independent cohort, ensuring unbiased performance estimates.

We have also revised Table 2 to explicitly describe the hyperparameter grid explored in the inner-loop search and to report, for each experiment, the final selected values. In addition, we now provide code-level details sufficient to reproduce the search procedure and the corresponding implementation is provided in our public GitHub repository at https://github.com/ZKZ-Brain/EH-BrainGNN.

*3.For lines 149-150 “To create A, we binarized the FC matrix by transforming only the top 20-percentile absolute correlation values into ones, and the rest were transformed into zeros.”, please explain the rationale for selecting the 20% threshold (citing relevant literature). If it was empirically determined, please provide justification. Alternatively, it is suggested to test other thresholds and report their impact on results.*

**Response:** We thank the reviewer for raising this important point. In the revised manuscript, we now explicitly justify our choice of a 20% binarization threshold and assess its impact on both predictive performance and subtype discovery.

First, we note that percentile-based binarization in the range of 10–30% has been commonly adopted in related work to balance sparsity with sufficient signal retention in high-dimensional biological data, and we now cite these studies in the Methods section (refs. [1-3]). Motivated by this practice, we originally selected 20% as a moderate cutoff that avoids overly sparse representations while still emphasizing the most informative features. To empirically evaluate this choice, we performed an ablation study over five candidate thresholds (10%, 15%, 20%, 25% and 30%) under all three evaluation schemes used in this work: (i) 10-fold cross-validation, (ii) leave-one-site-out cross-validation, and (iii) training on the principal dataset with testing on the independent validation cohort. The results are summarized in Supplementary Figure 7 and Supplementary Tables 5–6. Across all three schemes, the 20% threshold consistently achieved the highest or near-highest mean AUC, with only modest fluctuations in performance at neighboring thresholds.

**Supplementary Figure 7 The impact of graph construction threshold on 10-fold cross-validation.**

**Supplementary Table 5 The impact of graph construction threshold on leave-one-site cross-validation.**

| **Threshold** | **10%** | **15%** | **20%** | **25%** | **30%** |
| --- | --- | --- | --- | --- | --- |
| **Site1** | 0.56 | 0.55 | **0.63** | **0.63** | **0.63** |
| **Site2** | 0.73 | 0.70 | **0.80** | 0.67 | 0.67 |
| **Site3** | 0.80 | 0.73 | **0.88** | 0.71 | 0.71 |
| **Site4** | 0.72 | 0.71 | **0.75** | 0.69 | 0.68 |
| **Site5** | 0.67 | 0.68 | **0.70** | 0.64 | 0.67 |
| **Site6** | 0.64 | 0.63 | **0.69** | 0.68 | 0.63 |
| **Site7** | 0.72 | 0.66 | **0.75** | 0.65 | 0.68 |
| **Site8** | **0.73** | **0.73** | 0.70 | 0.65 | 0.62 |
| **Site9** | **0.78** | 0.75 | **0.78** | **0.78** | **0.78** |
| **Site10** | 0.68 | 0.69 | **0.72** | 0.71 | 0.67 |
| **Site11** | 0.82 | 0.81 | 0.84 | **0.85** | 0.84 |
| **Site12** | 0.62 | 0.61 | **0.67** | 0.61 | 0.65 |
| **Site13** | 0.65 | 0.69 | **0.73** | 0.69 | 0.65 |
| **Site14** | 0.60 | 0.59 | **0.63** | 0.59 | 0.60 |
| **Site15** | 0.55 | 0.59 | **0.68** | 0.59 | 0.61 |
| **Site16** | 0.61 | 0.53 | **0.76** | 0.59 | 0.63 |
| **Site17** | 0.64 | 0.64 | 0.64 | **0.73** | **0.73** |
| **Mean** | 0.68 | 0.66 | **0.73** | 0.67 | 0.67 |

**Supplementary Table 6 The impact of graph construction threshold on generalization performance in the independent validation dataset using the principal dataset.**

| **Threshold** | **10%** | **15%** | **20%** | **25%** | **30%** |
| --- | --- | --- | --- | --- | --- |
| **HUH** | 0.65 | 0.65 | **0.73** | 0.66 | 0.63 |
| **HRC** | 0.72 | 0.74 | 0.74 | **0.75** | **0.75** |
| **HKH** | 0.63 | 0.60 | **0.66** | 0.63 | **0.66** |
| **COI** | 0.64 | 0.62 | **0.66** | 0.65 | 0.60 |
| **Mean** | 0.66 | 0.65 | **0.70** | 0.67 | 0.66 |

We further evaluated the influence of the binarization threshold on subtype discovery. Although individual sample assignments exhibited minor perturbations at the lowest and highest thresholds, the overall subtype architecture and the defining biological signatures of each subtype were conserved across the 10–30% range. In this context, the 20% threshold yielded a favorable compromise, providing highly stable subtype solutions with clear inter-subtype separation and consistently strong predictive performance.

[1] CI-GNN: A Granger Causality-Inspired Graph Neural Network for Interpretable Brain Network-Based Psychiatric Diagnosis. Neural Networks, 2024.

[2] BrainIB: Interpretable Brain Network-Based Psychiatric Diagnosis With Graph Information Bottleneck. IEEE Transactions on Neural Networks and Learning Systems, 2025.

[3] BPI-GNN: Interpretable brain network-based psychiatric diagnosis and subtyping. Neuroimage, 2024.

*4.Please describe the stability of CFDP results, or provide evidence of the stability and statistical significance of the subtype-specific explanatory edges (i.e., the ”top 20 edges” mentioned in line 252).*

**Response:** Thank you for this important suggestion. In the revised manuscript, we have added a systematic stability analysis for both the CFDP-derived subtypes and the subtype-specific “top 20 edges”.

- **Stability of CFDP subtypes**

We performed a bootstrap resampling procedure with 1,000 resamples. For each bootstrap sample of subjects (sampled with replacement), we re-ran the CFDP pipeline on the learned embeddings using the same hyperparameters and obtained a new clustering assignment. We then quantified the agreement between the original clustering and each bootstrap clustering using the adjusted Rand index (ARI) and normalized mutual information (NMI), and summarized the distribution of these values by reporting the mean, standard deviation, and 95% bootstrap confidence intervals. The resulting stability was substantial (ARI: mean = 0.523, 95% CI [0.136, 0.863]; NMI: mean = 0.543, 95% CI [0.198, 0.809]).

To determine whether this stability exceeds what would be expected by chance, we additionally generated a null distribution by repeating the same procedure after permuting the embeddings or labels. Comparisons between the observed and null distributions yielded large effect sizes (Cohen’s d for ARI = 3.13; Cohen’s d for NMI = 4.38), indicating that the CFDP-derived subtypes are markedly more reproducible than random clusterings. These results are reported in the Methods and Results section.

- **Stability of subtype-specific “top 20 edges”**

To quantify the robustness of the subtype-associated edges, we performed 1000 bootstrap resampling of subjects. For each resample, we recomputed the subject-level top-20 edges and aggregated them within each subtype to obtain resampled subtype-level top-20 sets, which were then compared with the reference set using the Jaccard index. The corresponding results are shown in Supplementary Figure S4.

As shown in Supplementary Fig. S4 A, the subtype-specific edge sets exhibited moderate-to-high stability across resamples: the mean Jaccard overlap was 0.433 (95% CI [0.250, 0.600]) for subtype 1, 0.405 (95% CI [0.176, 0.667]) for subtype 2, and 0.335 (95% CI [0.143, 0.538]) for subtype 3. Because both the reference and bootstrap-derived sets contain 20 edges, these values correspond to approximately 10–12 shared edges on average, indicating that more than half of the reported edges are consistently recovered across resamples, while the remaining edges form a more variable peripheral set. Notably, these overlaps are far above chance, as random selection of 20 edges from the full edge set yields Jaccard values close to zero, supporting the presence of a reproducible core of subtype-specific connections. Moreover, for each subtype, a substantial fraction of the reference top-20 edges showed high bootstrap selection frequencies (>0.5), with 19 of 20 edges in subtype 1, 16 of 20 in subtype 2, and 9 of 20 in subtype 3 exceeding this threshold, further highlighting a robust core set of connections (Supplementary Fig. S4 B–D).

**Supplementary Figure S4. The stability of the subtype-specific top-20 edges.** (A) the stability of the subtype-specific top-20 edges across bootstrap resamples quantified by the Jaccard index. (B) the bootstrap selection frequency of the reference top-20 edges for each subtype.

- **Permutation tests and multiple-comparison control**

To assess whether the observed edge selection frequencies and CFDP-derived subtypes could be explained by random variation, we performed a non-parametric, label-permutation test. Specifically, in each of 1,000 iterations we randomly permuted subtype labels across subjects and then repeated the full analysis pipeline under the permuted labels: we re-aggregated the explanation masks and reapplied the edge-selection procedure to construct a null distribution of selection frequencies for each edge, and we re-ran the CFDP clustering pipeline to obtain null distributions for the clustering stability metrics including ARI and NMI.

From these null distributions, we derived empirical p-values and corrected them for multiple comparisons using the Benjamini–Hochberg FDR procedure. Across the 1,000 permutations, empirical p-values for both the subtype-specific top-20 edges and the CFDP clustering stability metrics were consistently < 0.0001, indicating that the observed edge selection patterns and subtype structure deviate markedly from what would be expected under a random-label null model, and supporting the presence of a genuine and robust underlying biological signal.

*5.In the section“Module D: Subtype generator,”please supplement with an explicit threshold or rule used for selecting centers and determining the number of clusters on the decision graph. If possible, please report the evidence for obtaining three subtypes under this rule.*

**Response:** We thank the reviewer for this helpful suggestion. In the revised manuscript, we have substantially clarified how cluster centers and the number of clusters are determined and have added both visual and quantitative evidence supporting the choice of $K=3$ in both datasets.

First, we now explicitly describe the rules used with CFDP (clustering by fast search and find of density peaks) to select cluster centers from the decision graph. For each subject, CFDP computes a local density $\rho_{i}$ and a distance to the nearest point with higher density $\delta_{i}$. We plot $\delta_{i}$ against $\rho_{i}$ to obtain the decision graph and define a combined prominence score:

$$\gamma_{i}=\rho_{i}\times\delta_{i}.$$

Points with large values of both $\rho_{i}$ and $\delta_{i}$ correspond to high $\gamma_{i}$ and are treated as candidate cluster centers. For each dataset, we visualize the decision graph, identify the prominent corner region with high density and large distance, and select as centers those points with the largest $\gamma_{i}$. As shown in the decision graphs (Figure 5 A and C) and corresponding spatial distributions (Figure 5 B and D) that we now include for both datasets, three such prominent peaks are clearly present, leading to three CFDP centers in each case.

Second, in accordance with the editor’s request, we have added cluster quality analyses across a range of candidate values of $K$. Using the learned embeddings and the corresponding CFDP assignments, we compute the silhouette coefficient and Dunn index for different choices of $K$. In both datasets, $K=3$ either attains the highest silhouette coefficient or lies very close to the optimum, and it is also associated with higher Dunn values compared with neighboring values of $K$ (Figure 5 E-H). These results are now reported in the Results and illustrated in Figure 5 E-H that shows the silhouette and Dunn indices as functions of $K$, where three clusters emerge as the most parsimonious and well supported solution.

**Figure 5. Determination of the optimal number of CFDP subtypes.** (A-B) CFDP decision graphs for the two datasets. (C-D) Two dimensional embeddings illustrating the spatial distribution of subjects colored by CFDP subtype. (E-F) Silhouette coefficients as a function of the number of clusters ($K=2-9$). (G-H) Dunn indices for $K=2-9$.

*6.Considering that the REST-meta-MDD dataset includes clinical information such as medication status, please clarify whether these factors influence the subtyping results.*

**Response:** We appreciate the reviewer’s insightful comment regarding the potential influence of clinical covariates, particularly medication status, on the identified subtypes. In the revised manuscript, we have added additional sensitivity analyses addressing these concerns.

To test whether medication status affects the subtyping solution, we performed a sensitivity analysis in which medication was treated as a covariate. Specifically, we regressed out medication status from the graph embeddings and repeated the subtyping procedure. As shown in Supplementary Figure S11 A–B, the analysis again yielded three subtypes, and subtype assignments were highly stable: fewer than 2% of participants changed subtype relative to the original solution.

Additionally, we conducted an additional analysis restricted to medication-naïve participants and repeated the subtyping. As shown in Supplementary Figure S11 C–D, this restricted sample likewise produced three subtypes, with subtype assignments differing from the full-sample solution for fewer than 2% of participants.

**Supplementary Figure S11 The impact of medication status on subtype analysis. (A-B)** CFDP decision graph and spatial distribution of subtypes with medication included as a covariate. **(C-D)** CFDP decision graph and spatial distribution of subtypes with medication-naïve participants.

*7.In the section” Correspondences between functional signature and MDD symptoms,” the authors only reported R2. It is recommended to also provide corresponding significance levels.*

## Response: We thank the reviewer for these very helpful suggestions. We have revised the manuscript accordingly:

## 1. Significance testing for $\boldsymbol{R^{2}}$

## We now report permutation-based significance testing for all cross-validated $\boldsymbol{R^{2}}$ values. Specifically, for each predictive model we generated 1,000 null $\boldsymbol{R^{2}}$ values by repeating the entire analysis pipeline with randomly permuted symptom scores while keeping the functional features and model parameters identical. The observed $\boldsymbol{R^{2}}$ was then compared with this empirical null distribution to obtain a permutation-based *p* value, and multiple comparisons across symptom dimensions were controlled using false discovery rate (FDR) correction. The resulting permutation-based p values, together with the 95% confidence intervals (CIs) around the observed r², are summarized in Table 6.

## 2. Additional performance metric (MAE)

## To further characterize predictive performance, we have also added the mean absolute error (MAE) as a complementary metric to $\boldsymbol{R^{2}}$. MAE values are now reported in the updated table 6.

## Table 6. Subtype-specific clinical profiles for significantly different depression symptoms. The highest performance is highlighted with boldface.

| **Symptoms** | **Subtype1** | | | | **Subtype2** | | | | **Subtype3** | | | |
| --- | --- | --- | --- | --- | --- | --- | --- | --- | --- | --- | --- | --- |
|  | $\boldsymbol{R}^{\boldsymbol{2}}$ | **95% CI** | **MAE** | ***p* values** | $\boldsymbol{R}^{\boldsymbol{2}}$ | **95% CI** | **MAE** | ***p* values** | $\boldsymbol{R}^{\boldsymbol{2}}$ | **95% CI** | **MAE** | ***p* values** |
| Depressed Mood | 0.48 | [0.34, 0.60] | 0.55 | <0.0001 | 0.34 | [0.25, 0.43] | 0.65 | <0.0001 | **0.56** | [0.42, 0.71] | **0.39** | <0.0001 |
| Insomnia early | 0.46 | [0.33, 0.55] | 0.46 | <0.0001 | 0.37 | [0.28, 0.43] | 0.53 | <0.0001 | **0.56** | [0.28, 0.72] | **0.33** | <0.0001 |
| Insomnia middle | 0.48 | [0.35, 0.58] | 0.42 | <0.0001 | 0.35 | [0.26, 0.42] | 0.47 | <0.0001 | **0.54** | [0.30, 0.73] | **0.40** | <0.0001 |
| Agitation | 0.45 | [0.30, 0.54] | **0.47** | <0.0001 | 0.42 | [0.31, 0.51] | 0.52 | <0.0001 | **0.51** | [0.31, 0.70] | **0.47** | <0.0001 |
| Genital | 0.53 | [0.43, 0.61] | 0.43 | <0.0001 | 0.44 | [0.37, 0.52] | 0.47 | <0.0001 | **0.57** | [0.41, 0.68] | **0.40** | <0.0001 |
| Weight Loss | 0.40 | [0.29, 0.48] | **0.47** | <0.0001 | 0.34 | [0.25, 0.42] | 0.48 | <0.0001 | **0.57** | [0.43, 0.68] | **0.47** | <0.0001 |
| Insight | 0.44 | [0.31, 0.59] | 0.37 | <0.0001 | 0.36 | [0.29, 0.43] | 0.39 | <0.0001 | **0.56** | [0.43, 0.63] | **0.34** | <0.0001 |

*8.Please unify the terminology of abbreviations (e.g., SBN in Figure 5 and SCN in Table S3).*

**Response:** Corrected as suggested.

*Reviewer #2:*

*The authors proposed a novel ensemble hybrid deep-learning framework for brain network using the graph neural network (which they call EN-BrainGNN). They applied their framework to MDD classification and subtyping task in the multi-site and cross-nation setting using two large MDD database (Rest-META-MDD, N=1604, China and SRPBS, N=446, Japan). In the supervised classification task, it achieved 72% accuracy in the independent validation dataset showing the strong across-site and cross-national generalization. In the unsupervised clustering task, they identified three reproducible biological subtypes across two datasets.*

*This study is an important contribution demonstrating that representation learning with deep learning models for MDD biomarker development can generalize across multi-site and cross-national datasets, and is applicable to both classification and subtyping. In previous biomarker studies using functional connectivity (FC) as features, progress had been made in achieving multi-site generalization for classification and subtyping. However, the performance had nearly reached an upper bound, and changing classification algorithms alone did not yield significant improvements. On the other hand, many studies employing deep learning lacked evaluation with independent datasets. This study showed, using the independent validation dataset, that graph embedding features learned by deep learning not only improve classification accuracy but also enable reproducible subtyping. Thus, it represents an important step toward applying deep learning frameworks to clinical research.*

*Although the results presented in this paper are highly significant, several concerns remain. First, the explanation of the deep learning framework is insufficient. Second, there is no description of methods for interpreting the learned features. Third, an analysis is needed to clarify why deep learning, which typically involves a large number of parameters and is prone to overfitting, was able to achieve such robust representation learning. Addressing these questions would help bridge the gap between the proposed interpretable deep learning–based representation learning method and conventional machine learning approaches that use FC features.*

*[Major comments]*

*1. The section Overall framework of EH-BrainGNN is extremely difficult to follow. To improve readability, a thorough rewrite is necessary. For example, it would be clearer to first describe feature representation learning through the classification task, and then explain the downstream subtyping task using the learned features. A schematic diagram of the overall deep network architecture, including the number of model parameters, is also needed. In addition, the role of the subgraph is not explained sufficiently and should be clarified.*

**Response:** We thank the reviewer for pointing this out. We have thoroughly rewritten the “Overall framework of EH-BrainGNN” section to improve readability and to better reflect the learning pipeline. Specifically, we now first describe feature representation learning driven by the supervised classification objective, including how the graph encoder produces node- and graph-level embeddings, and then present the downstream subtyping procedure that operates on the learned graph-level features (without affecting the classifier training). To further improve clarity, we have added a schematic diagram of the full deep network architecture with the number of trainable parameters for each module and in total (see Figure 3). Parameter counts are computed as the total number of trainable weights and biases in each learnable layer; parameter-free operations (e.g., ReLU, dropout, pooling/flattening) are counted as zero.

In addition, we have clarified the role of the subgraph generator. The subgraph module is integrated into the representation learning stage as an explainer/regularizer that samples discriminative subgraphs and is jointly optimized with the graph encoder. A mutual-information-based constraint is used to encourage the sampled subgraph to retain informative content from the original graph. We have added this explanation in the revised text and the schematic accordingly (Figure 3B).

**Figure 3 A schematic of the full network with parameter counts (A) and a clear statement of the role of the subgraph generator (B).**

*2. In Figures 5 and 6, the authors present edges associated with the three subtypes. However, it is not at all clear how these edges were derived from the learned embedding features. Please provide detailed explanation in the Methods section, and discuss the importance of interpretability in the deep learning framework for biomarker development.*

**Response:** Thank you very much for this valuable suggestion. In the revised manuscript, we have expanded the Methods section to provide a detailed description of how edges are selected from the explanation masks and embeddings. We have also revised the Discussion to further elaborate on the implications of the identified biomarkers for clinical translation and potential deployment.

**1. From embeddings to edge-level interpretability**

To link the learned embeddings to interpretable, edge-level connectivity patterns, we implemented a three-step procedure.

- **Computation of subject-level explanation masks**

For each subject $s$, we consider the input graph $G_{s}=(V_{s},E_{s})$ with functional connectivity edges $e\in E_{s}$. Using the **subgraph generator module** of our framework, we obtain an edge-wise explanation mask for subject $s$, that assigns to each edge a non-negative importance score

$m_{s}\left( e \right)\mathbb{\in R\geq}0, e\in E_{s}$,

which quantifies how strongly edge $e$contributes to the model’s prediction for that subject.

- **Subject-level edge selection (top-20 edges)**

Within each subject, we rank all edges in descending order of their explanation scores $m_{s}\left( e \right)$. Let $\left\{ e_{s,\left( 1 \right)},\ldots,e_{s,\left( K \right)} \right\}$ denote the $K$ edges with the largest mask values for subject $s$, i.e.

$$m_{s}\left( e_{s,\left( 1 \right)} \right)\geq m_{s}\left( e_{s,\left( 2 \right)} \right)\geq\ldots\geq m_{s}\left( e_{s,\left( K \right)} \right),$$

with $K=20$ in all experiments. We then define the subject-level top-$K$ edge set as

$$T_{s}=\left\{ e_{s,\left( 1 \right)},\ldots,e_{s,\left( K \right)} \right\}\subseteq E_{s},$$

which provides a sparse, subject-specific summary of the most influential connections.

- **Aggregation across subjects within each subtype**

Let $c\in\left\{ 1,\ldots,C \right\}$ index the CFDP-derived subtypes, and

$$S_{c}=\left\{ s:\text{subject} s \text{is assigned to subtype} c \right\},$$

denote the set of subjects in subtype $c$. For each edge $e$in the common edge index space, we compute a selection frequency

$$f_{c}\left( e \right)=\frac{1}{\left| S_{c} \right|}\sum_{s\in S_{c}} I\left[ e\in T_{s} \right],$$

where $I\left[ \cdot\right]$ denotes the indicator function, which equals 1 if the condition is satisfied and 0 otherwise.

**2. The implications of the identified biomarkers for clinical translation and potential deployment**

We have expanded the Discussion to clarify how the subtype-specific connectivity patterns identified by our framework can be viewed as candidate circuit-level biomarkers with potential implications for precision treatment in MDD.

First, our clustering of learned embeddings revealed three reproducible MDD subtypes characterized by distinct connectivity motifs: (i) a default mode network–cerebellum subtype, (ii) an insula–cingulum–temporal subtype, and (iii) a frontostriatal subtype. Because our edge-level explanations are derived directly from the predictive model, these subtype-specific patterns provide an interpretable link between latent representations and biologically meaningful circuits, rather than abstract, model-internal features.

Second, we now discuss how these circuit signatures may inform personalized target selection for neuromodulation, particularly in the context of rTMS, where current practice in MDD largely relies on “one-size-fits-all” dorsolateral prefrontal cortex (DLPFC) targeting and lacks principled strategies for individualized target choice. Our results suggest a testable precision-medicine framework: patients assigned to the default mode network–cerebellum subtype might benefit from cerebellar stimulation as a candidate target; those in the insula–cingulum–temporal subtype might be preferentially considered for insula- or salience-network–oriented targeting; and those in the frontostriatal subtype align more closely with conventional DLPFC-based stimulation protocols. We explicitly note in the revised Discussion that these implications are hypothesis-generating rather than prescriptive, and that rigorous prospective interventional trials would be required to determine whether subtype-guided rTMS targeting indeed improves treatment response.

Finally, we emphasise that the edge-level biomarkers identified by our explainable framework are (i) sparse, (ii) circuit-specific, and (iii) reproducible across resampling and permutation tests, properties that are desirable for translational biomarkers intended for clinical deployment. We have added text to highlight how this combination of interpretability and robustness moves beyond purely predictive models and points towards a mechanistically grounded, subtype-informed approach to biomarker development in depression.

*3. Although the best hyperparameters are reported, the sensitivity of the results to different parameter choices is not examined. In particular, the number of GNN layers and the hidden dimensions are likely to be the two most important parameters. Please provide classification performance results (both cross-validation and independent validation) by varying these two parameters. From another perspective, it is also essential to consider how many samples are required to obtain robust features using deep learning. Please compare classification performance while varying the number of training samples.*

**Response:** We thank the reviewer for this important suggestion. In the revised manuscript, we have systematically evaluated the impact of GNN architecture on performance and explicitly report both cross validation and independent validation results for the requested variants.

First, we varied the number of GNN layers (2, 3, 4, 5) and the hidden dimensionality (64, 128, 256), covering at least the configurations listed in Table 2. For each combination, we report cross validation performance and independent validation performance in the cross cohort setting (training on the principal dataset and testing on the independent validation dataset). The full results are now summarized in Supplementary Figure 8 and 9. Across both datasets and both evaluation schemes, the configuration with 5 layers and a 128-dimensional hidden space consistently achieved the best or near best performance, while the other settings showed only moderate fluctuations in accuracy and AUC. These findings support the claim that our model performance is robust to reasonable variations in architectural depth and capacity, and that the specific configuration reported in the main text is a well-supported choice rather than an isolated optimum.

**Supplementary Figure S8. The impact of GNN layers and hidden dimensions on 10-fold cross-validation performance.**

**Supplementary Figure S9. The impact of GNN layers and hidden dimensions on generalization performance in the independent validation dataset using the principal dataset.**

Second, as suggested, we have added a learning curve analysis in which we vary the size of the training set and plot test accuracy as a function of training sample size (Supplementary Figure 10). The results show a monotonic increase in test accuracy as the number of training subjects grows, with no evidence of abrupt degradation at larger sample sizes. This result indicates that the model continues to benefit from additional data and that the learned representations generalize increasingly well rather than overfitting to small training subsets. We now include this analysis in the Results to support our statement about robust representation learning.

**Supplementary Figure S10. Learning curve analysis.**

*[Minor comments]*

*1. [line 150] Please add explanation of the choice of top 20%-tile.*

**Response:** We thank the reviewer for raising this important point. In the revised manuscript, we now explicitly justify our choice of a 20% binarization threshold and assess its impact on both predictive performance and subtype discovery.

First, we note that percentile-based binarization in the range of 10–30% has been commonly adopted in related work to balance sparsity with sufficient signal retention in high-dimensional biological data, and we now cite these studies in the Methods section (refs. [1-3]). Motivated by this practice, we originally selected 20% as a moderate cutoff that avoids overly sparse representations while still emphasizing the most informative features. To empirically evaluate this choice, we performed an ablation study over five candidate thresholds (10%, 15%, 20%, 25% and 30%) under all three evaluation schemes used in this work: (i) 10-fold cross-validation, (ii) leave-one-site-out cross-validation, and (iii) training on the principal dataset with testing on the independent validation cohort. The results are summarized in Supplementary Figure 7 and Supplementary Tables 5–6. Across all three schemes, the 20% threshold consistently achieved the highest or near-highest mean AUC, with only modest fluctuations in performance at neighboring thresholds.

**Supplementary Figure 7 The impact of graph construction threshold on 10-fold cross-validation.**

**Supplementary Table 5 The impact of graph construction threshold on leave-one-site cross-validation.**

| **Threshold** | **10%** | **15%** | **20%** | **25%** | **30%** |
| --- | --- | --- | --- | --- | --- |
| **Site1** | 0.56 | 0.55 | **0.63** | **0.63** | **0.63** |
| **Site2** | 0.73 | 0.70 | **0.80** | 0.67 | 0.67 |
| **Site3** | 0.80 | 0.73 | **0.88** | 0.71 | 0.71 |
| **Site4** | 0.72 | 0.71 | **0.75** | 0.69 | 0.68 |
| **Site5** | 0.67 | 0.68 | **0.70** | 0.64 | 0.67 |
| **Site6** | 0.64 | 0.63 | **0.69** | 0.68 | 0.63 |
| **Site7** | 0.72 | 0.66 | **0.75** | 0.65 | 0.68 |
| **Site8** | **0.73** | **0.73** | 0.70 | 0.65 | 0.62 |
| **Site9** | **0.78** | 0.75 | **0.78** | **0.78** | **0.78** |
| **Site10** | 0.68 | 0.69 | **0.72** | 0.71 | 0.67 |
| **Site11** | 0.82 | 0.81 | 0.84 | **0.85** | 0.84 |
| **Site12** | 0.62 | 0.61 | **0.67** | 0.61 | 0.65 |
| **Site13** | 0.65 | 0.69 | **0.73** | 0.69 | 0.65 |
| **Site14** | 0.60 | 0.59 | **0.63** | 0.59 | 0.60 |
| **Site15** | 0.55 | 0.59 | **0.68** | 0.59 | 0.61 |
| **Site16** | 0.61 | 0.53 | **0.76** | 0.59 | 0.63 |
| **Site17** | 0.64 | 0.64 | 0.64 | **0.73** | **0.73** |
| **Mean** | 0.68 | 0.66 | **0.73** | 0.67 | 0.67 |

**Supplementary Table 6 The impact of graph construction threshold on generalization performance in the independent validation dataset using the principal dataset.**

| **Threshold** | **10%** | **15%** | **20%** | **25%** | **30%** |
| --- | --- | --- | --- | --- | --- |
| **HUH** | 0.65 | 0.65 | **0.73** | 0.66 | 0.63 |
| **HRC** | 0.72 | 0.74 | 0.74 | **0.75** | **0.75** |
| **HKH** | 0.63 | 0.60 | **0.66** | 0.63 | **0.66** |
| **COI** | 0.64 | 0.62 | **0.66** | 0.65 | 0.60 |
| **Mean** | 0.66 | 0.65 | **0.70** | 0.67 | 0.66 |

We further evaluated the influence of the binarization threshold on subtype discovery. Although individual sample assignments exhibited minor perturbations at the lowest and highest thresholds, the overall subtype architecture and the defining biological signatures of each subtype were conserved across the 10–30% range. In this context, the 20% threshold yielded a favorable compromise, providing highly stable subtype solutions with clear inter-subtype separation and consistently strong predictive performance.

[1] CI-GNN: A Granger Causality-Inspired Graph Neural Network for Interpretable Brain Network-Based Psychiatric Diagnosis. Neural Networks, 2024.

[2] BrainIB: Interpretable Brain Network-Based Psychiatric Diagnosis With Graph Information Bottleneck. IEEE Transactions on Neural Networks and Learning Systems, 2025.

[3] BPI-GNN: Interpretable brain network-based psychiatric diagnosis and subtyping. Neuroimage, 2024.

*2. [line 151] By definition, rho(k,k) is always one for any k. Does it cause any problem?*

**Response:** We thank the reviewer for raising this point. Indeed, by definition $\rho_{kk}=1$ for any ROI $k$. In our formulation this does not cause a practical problem, for two reasons:

**1. It does not introduce label leakage.** The diagonal entries are constant across all subjects and classes, so they do not carry discriminative information that could artificially inflate performance.

**2.** **Self-correlations are not used as edges.** The adjacency matrix $A$ is constructed from the **off-diagonal** FC values (we do not include self-loops in $A$), so $\rho_{kk}$ does not affect the graph topology.

*3. [line 255] What does “leave-one-out ridge regression model” mean? Does it mean leave-one-site-out cross validation using the ridge regression model?*

**Response:** We appreciate the reviewer’s request for clarification. In the manuscript, “leave-one-out ridge regression model” refers to leave-one-subject-out cross-validation, not leave-one-site-out cross-validation. Specifically, for each fold, we trained the ridge regression model on all participants except one and then predicted the symptom score for the held-out participant. We have revised the text to explicitly state “leave-one-subject-out cross-validation with ridge regression” and have updated the Methods and Results sections accordingly to avoid ambiguity.

*4. [line 257] How many dimensions after PCA ?*

**Response:** We thank the reviewer for pointing out this comment. In PCA-based analyses, we retained nine principal components, which together explained approximately 95% of the variance in the original feature space. This has now been explicitly stated in the Methods.

*5. [line 277] How did the authors determine the final setting? Were these parameters fixed without looking at the independent validation data? If not, overfitting can be expected to some extent. Please clarify and discuss.*

**Response:** We thank the reviewer for this constructive comment. In the revised manuscript, we have substantially expanded the subsection “Training, testing and hyperparameter optimization” to provide a precise and reproducible description of our model selection procedure.

Specifically, we now clarify that all hyperparameters were tuned using a nested cross-validation scheme. For each experiment, we performed an outer 10-fold cross-validation. Within each outer training split, we conducted an inner 5-fold cross-validation to optimize hyperparameters. For every candidate hyperparameter configuration defined in Table 2 (including learning rate, batch size, number of layers, number of hidden units, weight decay, dropout rate, and other model-specific parameters), the model was trained on 4 inner folds and evaluated on the remaining fold, cycling through all inner folds. We then selected the configuration that maximized the mean accuracy across the inner validation folds. After identifying the best hyperparameter configuration for a given outer fold, we retrained the model on the full outer training set using this fixed configuration. Early stopping was applied with a patience of 10 epochs, monitoring the validation accuracy computed on an internal validation split drawn exclusively from the outer training data. Importantly, neither the outer test folds nor the independent validation cohort were used at any stage for hyperparameter tuning, model selection, or calibration of early-stopping criteria. All hyperparameters and early-stopping rules were therefore fixed before scoring any outer test fold or the independent cohort, ensuring unbiased performance estimates.

We have also revised Table 2 to explicitly describe the hyperparameter grid explored in the inner-loop search and to report, for each experiment, the final selected values. In addition, we now provide code-level details sufficient to reproduce the search procedure and the corresponding implementation is provided in our public GitHub repository at https://github.com/ZKZ-Brain/EH-BrainGNN.

*6. [line 279] Did the authors apply the identical procedure (network architecture, hyperparameters, harmonization) to two different datasets independently? Please clarify.*

**Response:** We thank the reviewer for the question and have clarified this in the revised Methods. We used an identical network architecture for both datasets: a 5-layer GIN with 128 hidden dimensions, selected based on performance benchmarking and then fixed for all experiments.

Hyperparameters were not identical across datasets. Because the two public cohorts differ in data characteristics and available metadata, we tuned hyperparameters within each dataset (using the same tuning strategy and search ranges) and report the final settings for each dataset in Table 2.

## Table 2. Range of hyper-parameters and final specification for EH-BrainGNN

| Hyper-parameter | Range Examined | Final Specification (10-fold cross validation) | Final Specification (leave-one-site-out cross validation) | Final Specification (cross-national validation) |
| --- | --- | --- | --- | --- |
| Epoch | [350] | 350 | 350 | 350 |
| #GNN Layers | [2,3,4,5,6] | 5 | 5 | 5 |
| #MLP Layers | [2,3,4] | 2 | 2 | 2 |
| #Hidden Dimensions | [64,128,256,512] | 128 | 128 | 128 |
| Learning Rate | [1e-2,1e-3,1e-4] | 1e-3 | 1e-3 | 1e-3 |
| Batch Size | [32,64,128] | 64 | 64 | 64 |
| Weight Decay | [5e-3,5e-4] | 5e-3 | 5e-4 | 5e-3 |
| Dropout | [0.5] | 0.5 | 0.5 | 0.5 |
| $\tau$ | [0.1,1] | 1 | 1 | 1 |
| $\lambda_{1}$ | [1e-2,1e-3, 1e-4,1e-5] | 1e-4 | 1e-4 | 1e-3 |
| $\lambda_{2}$ | [1e-2,1e-3,1e-4,1e-5] | 1e-5 | 1e-4, | 1e-4, |
| $\lambda_{3}$ | [1e-2,1e-3,1e-4,1e-5] | 1e-4 | 1e-3 | 1e-4 |

For ComBat harmonization, we applied the same harmonization framework but used the available covariates in each cohort. In the principal dataset, ComBat was performed with covariates age, sex, education, and head motion. In the independent validation dataset, education was not available, so ComBat was performed with age, sex, and head motion. We now explicitly state this covariate difference and its rationale in the revised manuscript.

*7. [line 287] The description about Fig.3(a) seems to be incorrect.*

**Response:** We thank the reviewer for pointing this out. We re-checked the text describing Fig. 3a and found that the previous description was indeed inaccurate. We have corrected the wording to match the figure content and updated the corresponding figure caption and main-text references in the revised manuscript (Results section, Fig. 3a caption). No results or conclusions are affected; this change is purely a clarification/correction of the description.

*8. [line 292-293] Figure 4 (B) showed clear separation of MDD and HCs. If this is true, the classification performance should be much higher. Why is this not the case ?*

**Response:** We thank the reviewer for this important observation. The apparent separation in Fig. 4B reflects a visualization of the learned representations using all available subjects (and a low-dimensional projection for display). In contrast, the reported classification performance is computed strictly on held-out test data under our cross-validation/generalization protocol. Therefore, the visual separation in Fig. 4B should not be interpreted as the expected test-set separability.

In addition, the 2D projection used for visualization can exaggerate perceived separation and does not directly correspond to the decision boundary used by the classifier in the full embedding space. To avoid confusion, we have clarified in the revised manuscript and figure caption that Fig. 4B is shown for qualitative illustration using the full dataset, whereas performance metrics are reported on held-out test sets.

*9. [line 298-299] Did the authors also apply resulting parameters to test data to harmonize test data?*

**Response:** We appreciate the reviewer’s question regarding the harmonization procedure. Yes, we applied the resulting harmonization parameters to the test data. Specifically, for each cross-validation fold, harmonization parameters were estimated only on the training set, and these fitted parameters were then applied to the held-out test set to obtain harmonized test features. The test data were not used to re-estimate or update the harmonization model, thereby avoiding information leakage across folds.

*10. [line 300-301] In Figure 3(c), the plot shows ranking, whereas the text describes accuracy. This discrepancy is confusing. Please unify the presentation—either by reporting both as accuracy or both as ranking.*

**Response:** We thank the reviewer for pointing out this inconsistency. We agree that the current presentation of Fig. 3c is confusing because the plot is shown as ranking while the text discusses accuracy. In the revised manuscript, we have unified the presentation by reporting results consistently in both the figure and the accompanying text.

*11. [line 310-311] The same as the comment 9 above. Did the authors also apply resulting parameters to the independent validation data to harmonize them?*

**Response:** Yes. Consistent with the cross-validation procedure, harmonization parameters were estimated in the discovery cohort and then directly applied to the independent validation cohort to obtain harmonized features. The validation data were not used to refit or update the harmonization model, and we now state this explicitly in the Methods.

*12. [line 323] The details of CFDP algorithm is lacking. This method is known to be sensitive to the cut-off parameters to estimate density and distance. How to automatically determine the number clusters is not explained. Please add explanation in the method section.*

**Response:** We thank the reviewer for this helpful suggestion. In the revised manuscript, we have substantially clarified how cluster centers and the number of clusters are determined and have added both visual and quantitative evidence supporting the choice of $K=3$ in both datasets.

We now explicitly describe the rules used with CFDP (clustering by fast search and find of density peaks) to select cluster centers from the decision graph. For each subject, CFDP computes a local density $\rho_{i}$ and a distance to the nearest point with higher density $\delta_{i}$. We plot $\delta_{i}$ against $\rho_{i}$ to obtain the decision graph and define a combined prominence score:

$$\gamma_{i}=\rho_{i}\times\delta_{i}.$$

Points with large values of both $\rho_{i}$ and $\delta_{i}$ correspond to high $\gamma_{i}$ and are treated as candidate cluster centers. For each dataset, we visualize the decision graph, identify the prominent corner region with high density and large distance, and select as centers those points with the largest $\gamma_{i}$. As shown in the decision graphs (Figure 5 A and C) and corresponding spatial distributions (Figure 5 B and D) that we now include for both datasets, three such prominent peaks are clearly present, leading to three CFDP centers in each case.

**Figure 5. Determination of the optimal number of CFDP subtypes.** (A-B) CFDP decision graphs for the two datasets. (C-D) Two dimensional embeddings illustrating the spatial distribution of subjects colored by CFDP subtype. (E-F) Silhouette coefficients as a function of the number of clusters ($K=2-9$). (G-H) Dunn indices for $K=2-9$.

*13. [line 337-338] Lines in Figure5D are too thin and weak to see. Please increase visibility of the graphs.*

**Response:** We thank the reviewer for pointing this out. In the revised figure, we have increased the line thickness and optimized the rendering resolution to improve readability, while keeping the underlying results unchanged.

*14. [line 364] There are not scatter plots in the manuscript. Actually scatter plots are very helpful to understand how accurate it is. Please add the scatter plots in the manuscript.*

**Response:** As requested, we have added scatter plots showing predicted versus observed symptom scores (Supplementary Figure S5).

**Supplementary Figure S5. The scatter plots of predicted vs observed HAMD scores.**

*15. [line505-507] The caption seems to be wrong.*

**Response:** Corrected as suggested.

*Reviewer #3:*

*This is a timely and ambitious study that applies graph neural networks and clustering to classify and subtype MDD across large multi-site datasets. The technical design is strong, and the effort to test generalization across sites and even across countries is a real strength. That said, the work is not yet ready for publication without further validation. Reported accuracy is encouraging but not sufficient for clinical use, the subtyping results need stronger benchmarking and replication, and some key modeling choices require sensitivity checks. The discussion also needs to frame the findings more cautiously and acknowledge limitations more fully.*

*Abstract*

*The study proposes a framework that applies graph neural networks and clustering to classify and subtype major depressive disorder using resting-state fMRI data. The approach was tested on two large datasets from China and Japan, achieving 73 percent accuracy and identifying three reproducible subtypes. The effort to move diagnosis toward biological markers is important, and the use of multi-site data with cross-country validation is a strength.*

*However, the abstract does not provide enough information to fully assess the contribution. The reported accuracy lacks comparison with existing methods, and the clinical relevance of the identified subtypes is not established. It is also unclear how scanner and site differences were addressed, and what “strong” generalization means in terms of performance metrics. The abstract is heavy on technical terms, which may limit accessibility. A relationship to clinical outcomes would improve the paper.*

**Response:** We thank the reviewer for this constructive feedback and agree that the original abstract did not provide sufficient information to assess the contribution. In the revised manuscript, we have substantially rewritten the abstract to (i) explicitly state the comparison against representative baselines and summarize the key performance metrics (ACC, and where relevant F1), (ii) clarify what we mean by “generalization” by specifying the evaluation protocols (leave-one-site-out and cross-national testing) and the corresponding metrics, (iii) briefly describe how site variability was handled through a standardized preprocessing pipeline and site-aware validation (and Combat harmonization), and (iv) reduce technical jargon by describing clustering in more accessible terms. We also toned down claims about clinical utility and now clearly state that the identified subtypes are biologically interpretable and reproducible, while their associations with clinical outcomes remain to be established in future prospective studies. In the revised manuscript, we have revised abstract as below:

“Major depressive disorder (MDD) remains clinically diagnosed based on subjective symptoms rather than objective neurobiological markers, which limits diagnostic accuracy and the ability to tailor treatment. We present an ensemble hybrid framework that integrates graph neural networks (GNN) with unsupervised clustering to classify and subtype MDD using resting-state functional connectivity (rs-fMRI) profiles. A GNN was trained to distinguish MDD from healthy controls using functional connectivity derived brain graphs, and the resulting subject level embeddings were clustered to uncover subtype structure. We evaluated the approach on two public multisite cohorts, REST-meta-MDD (China; N = 1,604; 17 sites) and SRPBS (Japan; N = 446; 4 sites), using leave-one-site-out cross-validation and cross-national transfer. The classifier achieved 0.73 leave-one-site-out accuracy on REST-meta-MDD and retained 0.78 sensitivity when transferred from the Chinese to the Japanese cohort, outperforming BrainIB and CI GNN under the same protocol. To mitigate site related confounds, we applied a standardized preprocessing pipeline and ComBat harmonization. Clustering consistently identified three MDD subtypes with distinct connectivity signatures involving the default mode network and cerebellum, the insula-cingulum temporal circuit, and frontostriatal circuitry. These findings provide a reproducible and biologically interpretable stratification of MDD. Prospective studies will be needed to link these subtypes to treatment response and other clinically meaningful outcomes.”

*Introduction*

*The introduction highlights the limitations of current approaches to diagnosing and treating major depressive disorder. The authors point out that DSM-based definitions rely on subjective symptoms, which are heterogeneous and overlap with other disorders, limiting progress in treatment. They frame their work within the Research Domain Criteria initiative, emphasizing the need to redefine psychiatric disorders based on neural circuits and biological markers. Prior studies using functional connectivity and machine learning are discussed, and the challenges of poor generalization across datasets are noted. This sets up the rationale for the ensemble framework that combines supervised graph neural networks with unsupervised clustering, which the authors aim to validate across two large datasets.*

*While the introduction provides useful background, it is lengthy and somewhat repetitive. Several points about heterogeneity and generalization are made multiple times, and the description of the proposed framework is more detailed than what is typically expected in this section. Readers would benefit from a clearer focus on three elements: the problem of symptom-based diagnosis, the shortcomings of earlier neuroimaging classifiers, and the need for a more generalizable framework. Simplifying the language, and moving methodological details to later sections would imprve this section, make it aligned with public health readership.*

**Response:** Thank you for this thoughtful and constructive feedback. We agree that the Introduction was longer than necessary and that several points (particularly symptom heterogeneity and cross-dataset generalization) were reiterated. We have therefore substantially streamlined and refocused the Introduction to better align with a broad, public-health–oriented readership.

Specifically, we made the following revisions:

**1. Sharpened the narrative around the clinical problem.**

We now state the limitation of symptom-based (DSM) definitions succinctly up front, emphasizing heterogeneity and overlap without repeating these points across multiple paragraphs.

**2. Condensed the review of prior neuroimaging classifiers.**

We shortened the background on FC + machine-learning studies and focused on the key gap most relevant to our study: limited generalizability across datasets/sites.

**3. Reduced methodological detail in the Introduction.**

We removed or abbreviated implementation-level descriptions of the ensemble framework (e.g., model components and processing steps) and moved these details to the Methods/Results. The Introduction now provides only a high-level overview of the approach and the central hypothesis.

*Methods*

*The methods make strong use of large datasets and advanced graph-based models, but several design choices need deeper validation to ensure robustness. First, the binarization of functional connectivity by keeping only the top 20 percent of correlations is a strong modeling assumption that can heavily influence graph topology. The authors should test multiple thresholds or alternative weighting schemes (e.g., weighted adjacency matrices or sparsity-inducing regularization) to show that the results are not an artifact of this choice. Similarly, the reliance on Pearson correlation as the similarity metric could be complemented by partial correlations or other measures that better capture direct connectivity. Sensitivity analyses here would make the graph construction pipeline more defensible.*

**Response:** We thank the reviewer for raising this important point. In the revised manuscript, we now explicitly justify our choice of a 20% binarization threshold and assess its impact on both predictive performance and subtype discovery. We additionally evaluate an alternative connectivity metric beyond Pearson correlation.

- **Sensitivity to threshold choice**

First, we note that percentile-based binarization in the range of 10–30% has been commonly adopted in related work to balance sparsity with sufficient signal retention in high-dimensional biological data, and we now cite these studies in the Methods section (refs. [1-3]). Motivated by this practice, we originally selected 20% as a moderate cutoff that avoids overly sparse representations while still emphasizing the most informative features. To empirically evaluate this choice, we performed an ablation study over five candidate thresholds (10%, 15%, 20%, 25% and 30%) under all three evaluation schemes used in this work: (i) 10-fold cross-validation, (ii) leave-one-site-out cross-validation, and (iii) training on the principal dataset with testing on the independent validation cohort. The results are summarized in Supplementary Figure 7 and Supplementary Tables 5–6. Across all three schemes, the 20% threshold consistently achieved the highest or near-highest mean AUC, with only modest fluctuations in performance at neighboring thresholds.

**Supplementary Figure 7 The impact of graph construction threshold on 10-fold cross-validation.**

**Supplementary Table 5 The impact of graph construction threshold on leave-one-site cross-validation.**

| **Threshold** | **10%** | **15%** | **20%** | **25%** | **30%** |
| --- | --- | --- | --- | --- | --- |
| **Site1** | 0.56 | 0.55 | **0.63** | **0.63** | **0.63** |
| **Site2** | 0.73 | 0.70 | **0.80** | 0.67 | 0.67 |
| **Site3** | 0.80 | 0.73 | **0.88** | 0.71 | 0.71 |
| **Site4** | 0.72 | 0.71 | **0.75** | 0.69 | 0.68 |
| **Site5** | 0.67 | 0.68 | **0.70** | 0.64 | 0.67 |
| **Site6** | 0.64 | 0.63 | **0.69** | 0.68 | 0.63 |
| **Site7** | 0.72 | 0.66 | **0.75** | 0.65 | 0.68 |
| **Site8** | **0.73** | **0.73** | 0.70 | 0.65 | 0.62 |
| **Site9** | **0.78** | 0.75 | **0.78** | **0.78** | **0.78** |
| **Site10** | 0.68 | 0.69 | **0.72** | 0.71 | 0.67 |
| **Site11** | 0.82 | 0.81 | 0.84 | **0.85** | 0.84 |
| **Site12** | 0.62 | 0.61 | **0.67** | 0.61 | 0.65 |
| **Site13** | 0.65 | 0.69 | **0.73** | 0.69 | 0.65 |
| **Site14** | 0.60 | 0.59 | **0.63** | 0.59 | 0.60 |
| **Site15** | 0.55 | 0.59 | **0.68** | 0.59 | 0.61 |
| **Site16** | 0.61 | 0.53 | **0.76** | 0.59 | 0.63 |
| **Site17** | 0.64 | 0.64 | 0.64 | **0.73** | **0.73** |
| **Mean** | 0.68 | 0.66 | **0.73** | 0.67 | 0.67 |

**Supplementary Table 6 The impact of graph construction threshold on generalization performance in the independent validation dataset using the principal dataset.**

| **Threshold** | **10%** | **15%** | **20%** | **25%** | **30%** |
| --- | --- | --- | --- | --- | --- |
| **HUH** | 0.65 | 0.65 | **0.73** | 0.66 | 0.63 |
| **HRC** | 0.72 | 0.74 | 0.74 | **0.75** | **0.75** |
| **HKH** | 0.63 | 0.60 | **0.66** | 0.63 | **0.66** |
| **COI** | 0.64 | 0.62 | **0.66** | 0.65 | 0.60 |
| **Mean** | 0.66 | 0.65 | **0.70** | 0.67 | 0.66 |

We further evaluated the influence of the binarization threshold on subtype discovery. Although individual sample assignments exhibited minor perturbations at the lowest and highest thresholds, the overall subtype architecture and the defining biological signatures of each subtype were conserved across the 10–30% range. In this context, the 20% threshold yielded a favorable compromise, providing highly stable subtype solutions with clear inter-subtype separation and consistently strong predictive performance.

- **Alternative connectivity metrics beyond Pearson correlation**

Beyond Pearson correlation, we also examined partial correlation networks (estimated with appropriate regularization/shrinkage to ensure stable precision-matrix estimation) as an alternative that better reflects direct statistical dependencies. In our experiments, partial-correlation graphs yielded lower 10-fold cross-validation performance (mean accuracy 0.68) than Pearson-based graphs. Given this empirical result, we retained Pearson correlation as the default connectivity metric in the main analyses.

[1] CI-GNN: A Granger Causality-Inspired Graph Neural Network for Interpretable Brain Network-Based Psychiatric Diagnosis. Neural Networks, 2024.

[2] BrainIB: Interpretable Brain Network-Based Psychiatric Diagnosis With Graph Information Bottleneck. IEEE Transactions on Neural Networks and Learning Systems, 2025.

[3] BPI-GNN: Interpretable brain network-based psychiatric diagnosis and subtyping. Neuroimage, 2024.

*Second, while ComBat harmonization is appropriate for reducing site-related variability, the methods do not demonstrate that biological signal was preserved after adjustment. The authors should present diagnostics such as visualization of variance explained by site before and after harmonization, or checks that known case-control differences remain detectable. Without this, it is unclear whether harmonization might have inadvertently removed meaningful signal. The framework also integrates multiple loss components to balance sparsity, compactness, and predictive performance, but there is no discussion of how stable the results are under different weightings of these terms. A systematic ablation study of loss components and hyperparameter weighting would strengthen claims of robustness.*

**Response:** Thank you for raising these important concerns. In the revised manuscript, we have added two sets of analyses to (i) demonstrate that ComBat reduces site-related variability without removing biologically meaningful signal, and (ii) assess the contribution of each loss component to model robustness.

**1) Biological-signal preservation after ComBat.**
To verify that harmonization did not inadvertently remove disease-relevant effects, we performed a **case–control diagnostic** by comparing MDD versus HC functional connectivity before and after ComBat. Specifically, we computed edge-wise effect sizes (**Cohen’s** $d$) for all FC edges and visualized them as ROI×ROI heatmaps for the raw FC and the ComBat-adjusted FC. As shown in **Supplementary Fig. 6**, the spatial distribution and overall pattern of case–control differences were highly similar before and after harmonization, indicating preservation of the biological signal. Moreover, the change in effect sizes was minimal: the absolute difference $\left| \Delta d \right|$ between pre- and post-ComBat estimates was very small across edges, with a maximum of only **0.08**, further supporting that ComBat did not substantially attenuate disease-related variability.

**Supplementary Figure 6 Cohen’s** $\boldsymbol{d}$ **heatmaps for the raw FC and the ComBat-adjusted FC.**

**2) Ablation study of loss components (cross-national validation)**

We also addressed the stability of the framework with respect to the multi-term objective by conducting an ablation study in the cross-national validation setting (training on the principal cohort and testing on the independent cohort). As summarized in Supplementary Table 7, removing any individual loss component led to a marked degradation in performance, demonstrating that each term plays a substantial role in balancing sparsity with predictive accuracy and improving generalization. These results support the robustness of the proposed objective and justify the inclusion of all loss components in the final model.

**Supplementary Table 7 Ablation study of loss components (cross-national validation).**

| $L_{mask}$ | $L_{sps}$ | $L_{ent}$ | Accuracy |
| --- | --- | --- | --- |
| 🗴 | 🗴 | 🗴 | 0.61 |
| 🗸 | 🗴 | 🗴 | 0.67 |
| 🗴 | 🗸 | 🗴 | 0.61 |
| 🗴 | 🗴 | 🗸 | 0.64 |
| 🗸 | 🗸 | 🗴 | 0.63 |
| 🗴 | 🗸 | 🗸 | 0.61 |
| 🗸 | 🗴 | 🗸 | 0.66 |
| 🗸 | 🗸 | 🗸 | 0.73 |

*Finally, although the authors benchmark against a wide range of baselines, the evaluation strategy could be expanded. Reporting only average accuracy or AUC does not capture variability across folds, sites, or subgroups. Confidence intervals, variance across cross-validation folds, calibration measures, or subgroup-specific performance would provide a fuller picture of model reliability. The hyperparameter tuning strategy is also described only in broad terms; a more detailed account of the search space, convergence behavior, and variance across runs would increase confidence in reproducibility.*

**Response:** Thank you for this valuable suggestion. In the revised manuscript, we therefore expanded the evaluation and clarified the tuning strategy as follows.

**1) Reliability reporting beyond averages**

We now report performance with uncertainty and dispersion, including 95% confidence intervals and variance across 10-fold cross-validation folds. In addition, we added calibration diagnostics to provide a fuller picture of model reliability, reporting calibration metrics (e.g., ECE). Calibration was quantified using the 10-bin expected calibration error (ECE), which was 0.030 after Platt scaling. Platt scaling was fitted within each outer training fold using an internal calibration split and then applied to the corresponding outer test fold (or held-out site); neither the outer test fold nor the independent validation cohort was used to fit the calibrator, preventing information leakage. This indicates that our predictions are well calibrated and supports the reliability of our results.

2) **Detailed, leakage-free hyperparameter tuning (nested cross-validation)**

We now clarify that all hyperparameters were optimized using a nested cross-validation scheme. Specifically, we used an outer 10-fold cross-validation for unbiased evaluation. Within each outer training split, we ran an inner 5-fold cross-validation to select hyperparameters from the predefined search space (Table 2), including learning rate, batch size, number of layers, hidden units, weight decay and dropout, as well as model-specific parameters. For each candidate configuration, models were trained on four inner folds and validated on the remaining fold, cycling through all inner folds; we selected the configuration that maximized mean validation accuracy across the inner folds. Using this selected configuration, we then retrained on the full outer training set and evaluated on the outer test fold. Early stopping was applied with a patience of 10 epochs, monitoring validation accuracy computed on an internal validation split drawn exclusively from the outer training data. Importantly, outer test folds and the independent validation cohort were never used for hyperparameter tuning, model selection, or early-stopping calibration. Thus, all tuning decisions were finalized before scoring any outer test fold or the external cohort, ensuring unbiased performance estimates and reproducible model selection.

*Results*

*The results show that EH-BrainGNN outperformed standard classifiers and baseline GNNs in both the principal and validation datasets, with accuracies around 74 percent. The leave-one-site-out analysis adds credibility by testing generalization, and the cross-national validation from China to Japan is an important strength. However, the interpretation of these results would be stronger if the authors provided confidence intervals or statistical tests directly comparing EH-BrainGNN to the baselines. Right now, the claim of “significant improvements” rests on visual inspection of figures and tables rather than formal tests. It is also unclear whether the improvements are consistent across all subgroups or driven by a few high-performing sites, as performance varied substantially (for example, 88 percent accuracy at site 3 vs 64 percent at site 17). More detail on why such site variability occurs, and whether it relates to sample size, demographics, or data quality, would increase confidence that the model is robust rather than opportunistically fitting specific datasets.*

**Response:** We thank the reviewer for this detailed and constructive feedback.

- **Formal comparisons, confidence intervals, and effect sizes**

We now quantify superiority using paired statistical tests across the 10 cross-validation folds (fold-wise ACC as paired observations), comparing EH-BrainGNN against the next-best method in each setting. For the Principal Dataset, EH-BrainGNN versus BrainIB (ACC) yields *t*(9) = 4.3529, p = 0.0018, with Cohen’s *d* = 1.3765 and bootstrap 95% CI [0.7998, 2.9158]. For the Independent validation dataset, EH-BrainGNN versus CI-GNN (ACC) yields *t*(9) = 3.1623, *p* = 0.0115, with Cohen’s *d* = 1.0000 and bootstrap 95% CI [0.4454, 2.4244].

- **Site-wise variability and potential drivers**

We agree that performance heterogeneity across sites could reflect differences in sample size, demographics, data quality, and acquisition parameters. To address this, we will (i) provide site-wise performance summaries (including F1 and sensitivity) and (ii) add a concise summary of scanner/acquisition information (e.g., vendor/model, TR/TE where available) to contextualize site effects. We also expand the Discussion to outline plausible contributors to site variability and clarify that our primary generalization claims are supported by leave-one-site-out cross-validation and cross-cohort transfer, which reduce the likelihood that results are driven by a small number of high-performing sites.

*The t-SNE plots are used to argue that the learned graph embeddings capture diagnosis-specific structure, but this visualization alone is insufficient as evidence. The authors should complement t-SNE with quantitative measures such as cluster separability indices or supervised classification accuracy using embeddings alone. Similarly, while the subtyping analysis identifies three reproducible subgroups, the validity of these subtypes hinges on both statistical and clinical relevance. The paper shows some differences in HAMD and BDI scores, but effect sizes are small and not consistent across all comparisons. More importantly, the clustering method (CFDP) is not benchmarked against alternatives, leaving it unclear whether three is the optimal or most stable number of clusters. Without replication using a different clustering approach or statistical validation of cluster stability, the subtyping claims are less convincing.*

**Response:** Thank you for this important suggestion. In the revised manuscript, we have added a systematic stability analysis for both the CFDP-derived subtypes and the subtype-specific “top 20 edges”.

- **Stability of CFDP subtypes**

We performed a bootstrap resampling procedure with 1,000 resamples. For each bootstrap sample of subjects (sampled with replacement), we re-ran the CFDP pipeline on the learned embeddings using the same hyperparameters and obtained a new clustering assignment. We then quantified the agreement between the original clustering and each bootstrap clustering using the adjusted Rand index (ARI) and normalized mutual information (NMI), and summarized the distribution of these values by reporting the mean, standard deviation, and 95% bootstrap confidence intervals. The resulting stability was substantial (ARI: mean = 0.523, 95% CI [0.136, 0.863]; NMI: mean = 0.543, 95% CI [0.198, 0.809]).

To determine whether this stability exceeds what would be expected by chance, we additionally generated a null distribution by repeating the same procedure after permuting the embeddings or labels. Comparisons between the observed and null distributions yielded large effect sizes (Cohen’s d for ARI = 3.13; Cohen’s d for NMI = 4.38), indicating that the CFDP-derived subtypes are markedly more reproducible than random clusterings. These results are reported in the Methods and Results section.

- **Stability of subtype-specific “top 20 edges”**

To quantify the robustness of the subtype-associated edges, we performed 1000 bootstrap resampling of subjects. For each resample, we recomputed the subject-level top-20 edges and aggregated them within each subtype to obtain resampled subtype-level top-20 sets, which were then compared with the reference set using the Jaccard index. The corresponding results are shown in Supplementary Figure S4.

As shown in Supplementary Fig. S4 A, the subtype-specific edge sets exhibited moderate-to-high stability across resamples: the mean Jaccard overlap was 0.433 (95% CI [0.250, 0.600]) for subtype 1, 0.405 (95% CI [0.176, 0.667]) for subtype 2, and 0.335 (95% CI [0.143, 0.538]) for subtype 3. Because both the reference and bootstrap-derived sets contain 20 edges, these values correspond to approximately 10–12 shared edges on average, indicating that more than half of the reported edges are consistently recovered across resamples, while the remaining edges form a more variable peripheral set. Notably, these overlaps are far above chance, as random selection of 20 edges from the full edge set yields Jaccard values close to zero, supporting the presence of a reproducible core of subtype-specific connections. Moreover, for each subtype, a substantial fraction of the reference top-20 edges showed high bootstrap selection frequencies (>0.5), with 19 of 20 edges in subtype 1, 16 of 20 in subtype 2, and 9 of 20 in subtype 3 exceeding this threshold, further highlighting a robust core set of connections (Supplementary Fig. S5B–D).

**Supplementary Figure S4. The stability of the subtype-specific top-20 edges.** (A) the stability of the subtype-specific top-20 edges across bootstrap resamples quantified by the Jaccard index. (B) the bootstrap selection frequency of the reference top-20 edges for each subtype.

- **Permutation tests and multiple-comparison control**

To assess whether the observed edge selection frequencies and CFDP-derived subtypes could be explained by random variation, we performed a non-parametric, label-permutation test. Specifically, in each of 1,000 iterations we randomly permuted subtype labels across subjects and then repeated the full analysis pipeline under the permuted labels: we re-aggregated the explanation masks and reapplied the edge-selection procedure to construct a null distribution of selection frequencies for each edge, and we re-ran the CFDP clustering pipeline to obtain null distributions for the clustering stability metrics including ARI and NMI.

From these null distributions, we derived empirical p-values and corrected them for multiple comparisons using the Benjamini–Hochberg FDR procedure. Across the 1,000 permutations, empirical p-values for both the subtype-specific top-20 edges and the CFDP clustering stability metrics were consistently < 0.0001, indicating that the observed edge selection patterns and subtype structure deviate markedly from what would be expected under a random-label null model, and supporting the presence of a genuine and robust underlying biological signal.

*The subtype-specific connectivity findings are interesting, particularly the consistent thalamic connections across subtypes. Still, these results need stronger statistical support. Many of the connections shown in Figure 6 could emerge by chance given the large number of comparisons, and it is not clear whether corrections for multiple testing were applied at the level of subgraph analysis. The link between connectivity patterns and specific symptoms, while suggestive, would also benefit from more rigorous modeling. For example, the ridge regression analysis reports r² values above 0.3, but it is unclear whether these results hold after correcting for potential confounders like age, sex, and motion. Reporting performance on held-out subgroups or applying permutation testing would make the symptom-association findings more convincing.*

**Response:** We thank the reviewer for these very helpful suggestions. For the first point, we have addressed this in the response above. For the second point, we have revised the manuscript accordingly:

## 1. Significance testing for $\boldsymbol{R^{2}}$

## We now report permutation-based significance testing for all cross-validated $\boldsymbol{R^{2}}$ values. Specifically, for each predictive model we generated 1,000 null $\boldsymbol{R^{2}}$ values by repeating the entire analysis pipeline with randomly permuted symptom scores while keeping the functional features and model parameters identical. The observed $\boldsymbol{R^{2}}$ was then compared with this empirical null distribution to obtain a permutation-based *p* value, and multiple comparisons across symptom dimensions were controlled using false discovery rate (FDR) correction. The resulting permutation-based p values, together with the 95% confidence intervals (CIs) around the observed r², are summarized in Table 6.

## 2. Additional performance metric (MAE)

## To further characterize predictive performance, we have also added the mean absolute error (MAE) as a complementary metric to $\boldsymbol{R^{2}}$. MAE values are now reported in the updated table 6.

## Table 6. Subtype-specific clinical profiles for significantly different depression symptoms. The highest performance is highlighted with boldface.

| **Symptoms** | **Subtype1** | | | | **Subtype2** | | | | **Subtype3** | | | |
| --- | --- | --- | --- | --- | --- | --- | --- | --- | --- | --- | --- | --- |
|  | $\boldsymbol{R}^{\boldsymbol{2}}$ | **95% CI** | **MAE** | ***p* values** | $\boldsymbol{R}^{\boldsymbol{2}}$ | **95% CI** | **MAE** | ***p* values** | $\boldsymbol{R}^{\boldsymbol{2}}$ | **95% CI** | **MAE** | ***p* values** |
| Depressed Mood | 0.48 | [0.34, 0.60] | 0.55 | <0.0001 | 0.34 | [0.25, 0.43] | 0.65 | <0.0001 | **0.56** | [0.42, 0.71] | **0.39** | <0.0001 |
| Insomnia early | 0.46 | [0.33, 0.55] | 0.46 | <0.0001 | 0.37 | [0.28, 0.43] | 0.53 | <0.0001 | **0.56** | [0.28, 0.72] | **0.33** | <0.0001 |
| Insomnia middle | 0.48 | [0.35, 0.58] | 0.42 | <0.0001 | 0.35 | [0.26, 0.42] | 0.47 | <0.0001 | **0.54** | [0.30, 0.73] | **0.40** | <0.0001 |
| Agitation | 0.45 | [0.30, 0.54] | **0.47** | <0.0001 | 0.42 | [0.31, 0.51] | 0.52 | <0.0001 | **0.51** | [0.31, 0.70] | **0.47** | <0.0001 |
| Genital | 0.53 | [0.43, 0.61] | 0.43 | <0.0001 | 0.44 | [0.37, 0.52] | 0.47 | <0.0001 | **0.57** | [0.41, 0.68] | **0.40** | <0.0001 |
| Weight Loss | 0.40 | [0.29, 0.48] | **0.47** | <0.0001 | 0.34 | [0.25, 0.42] | 0.48 | <0.0001 | **0.57** | [0.43, 0.68] | **0.47** | <0.0001 |
| Insight | 0.44 | [0.31, 0.59] | 0.37 | <0.0001 | 0.36 | [0.29, 0.43] | 0.39 | <0.0001 | **0.56** | [0.43, 0.63] | **0.34** | <0.0001 |

*The validation analyses are a good addition, but they mostly confirm that choices like head motion regression, ROI atlas, and ComBat harmonization matter. What is missing is a sensitivity analysis quantifying how much each of these factors impacts performance. For instance, the switch from AAL to Power atlas improves results, but this is described only qualitatively. A systematic comparison, or at least reporting of effect sizes, would make the robustness checks more rigorous. Similarly, while harmonization improved some sites, the extent of improvement and its statistical significance are not fully shown. This makes it hard to assess whether the method is generally reliable or whether it still suffers from site-specific overfitting.*

**Response:** We thank the reviewer for this constructive critique and agree that a fully factorized sensitivity analysis would strengthen the robustness assessment. In the current revision, we provide a quantitative harmonization sensitivity analysis, because harmonization is the factor for which we can most reliably quantify site effects given the available metadata.

Specifically, we now report performance before versus after ComBat (e.g., ΔACC and, ΔAUC), stratified by site, and summarize the overall change across sites. These results are included in the revised Supplementary Materials and referenced in the Results.

We acknowledge that we did not perform a complete one factor at a time ablation for all components (e.g., head motion regression and atlas choice) in this revision. We have added this as a limitation and note that a systematic decomposition of the contributions of preprocessing choices and parcellation schemes will be an important direction for future work.

## Table 9. Effects of harmonization for the leave-one-site-out analysis.

| **Sites** | **ACC (Before Combat)** | **ACC (After Combat)** | $\boldsymbol{\Delta}\boldsymbol{ACC}$ | **AUC (Before Combat)** | **AUC (After Combat)** | $\boldsymbol{\Delta}\boldsymbol{AUC}$ |
| --- | --- | --- | --- | --- | --- | --- |
| Site1 | 0.54 | 0.63 | 0.09 | 0.54 | 0.63 | 0.09 |
| Site2 | 0.67 | 0.80 | 0.13 | 0.66 | 0.79 | 0.13 |
| Site3 | 0.85 | 0.88 | 0.03 | 0.86 | 0.89 | 0.03 |
| Site4 | 0.79 | 0.75 | -0.04 | 0.79 | 0.75 | -0.04 |
| Site5 | 0.71 | 0.70 | -0.01 | 0.73 | 0.70 | -0.03 |
| Site6 | 0.64 | 0.69 | 0.05 | 0.64 | 0.69 | 0.05 |
| Site7 | 0.70 | 0.75 | 0.05 | 0.73 | 0.70 | -0.03 |
| Site8 | 0.70 | 0.70 | 0 | 0.71 | 0.70 | -0.01 |
| Site9 | 0.64 | 0.78 | 0.14 | 0.65 | 0.78 | 0.13 |
| Site10 | 0.56 | 0.72 | 0.16 | 0.60 | 0.68 | 0.08 |
| Site11 | 0.82 | 0.84 | 0.02 | 0.81 | 0.82 | 0.01 |
| Site12 | 0.57 | 0.67 | 0.1 | 0.57 | 0.67 | 0.1 |
| Site13 | 0.59 | 0.73 | 0.14 | 0.64 | 0.73 | 0.09 |
| Site14 | 0.60 | 0.63 | 0.03 | 0.60 | 0.62 | 0.02 |
| Site15 | 0.65 | 0.68 | 0.03 | 0.65 | 0.67 | 0.02 |
| Site16 | 0.71 | 0.76 | 0.05 | 0.71 | 0.76 | 0.05 |
| Site17 | 0.64 | 0.64 | 0 | 0.64 | 0.65 | 0.01 |

*Overall, the result section is great, but can be better. To increase rigor, the authors should: (1) report confidence intervals and formal significance tests for performance comparisons, (2) provide quantitative validation of embedding discriminability and subtype stability, (3) apply multiple testing correction to connectivity results, and (4) quantify the effects of preprocessing choices in sensitivity analyses.*

*Discussion*

*The discussion highlights why the study is important, but some claims are stronger than the data allow. The cross-site and cross-country validation is a real strength, yet an accuracy of about 73 percent should be framed as encouraging but not sufficient for clinical application at this stage. A more cautious tone would make the contribution clearer without overstating its readiness.*

**Response:** We thank the reviewer for this thoughtful comment and agree that our original Discussion could be interpreted as overstating clinical readiness. We have revised the Discussion to adopt a more cautious and balanced tone. In particular, we now explicitly frame the ~0.73 accuracy as encouraging evidence of cross-site and cross-country generalization, but not sufficient for clinical deployment at this stage. We also clarify that EH-BrainGNN is currently a research prototype intended for methodological advancement and hypothesis generation, and that clinical translation would require prospective multi-site validation, calibration and threshold selection aligned with clinical use cases, assessment of failure modes and subgroup fairness, and regulatory and governance considerations. These revisions are reflected in the updated Discussion/Limitations section.

*The subtyping results are a valuable addition, especially given that three subtypes were consistently identified. Still, earlier studies have reported different numbers of subtypes depending on the method, which shows how unsettled this area remains. The authors should acknowledge that their findings may be influenced by the clustering approach and emphasize replication. Linking the subtypes more directly to treatment response or prognosis would also strengthen the case for clinical usefulness.*

**Response:** We thank the reviewer for this important comment. We agree that psychiatric subtyping remains method-dependent and that prior studies have reported varying numbers of subtypes depending on the dataset and clustering strategy. In the revised Discussion, we now explicitly acknowledge that our three-subtype solution may be influenced by the choice of clustering approach and representation, and we emphasize that the identified subtypes should be interpreted as reproducible connectivity phenotypes within the current cohorts rather than definitive biological categories.

To strengthen the emphasis on replication, we highlight that the subtypes were observed consistently across analyses and validated in an independent cohort, but we also note that further replication in additional multi-site datasets and prospective settings is essential. Finally, we agree that establishing clinical utility requires linking subtypes to clinically meaningful outcomes. We have therefore expanded the Discussion/Limitations to state that treatment response, prognosis, and longitudinal outcomes were not available in these public datasets, and that future work will prioritize testing whether the identified subtype signatures predict antidepressant response and other outcomes in prospective, well-phenotyped cohorts.

*The limitations deserve more depth. The absence of treatment outcome data is important, but so is the possibility that results are shaped by preprocessing steps, site variability, or model hyperparameters. Expanding on these points would make the discussion more balanced and credible.*

**Response:** We thank the reviewer for this important suggestion and agree that the Limitations section should be more comprehensive. We have expanded the revised Discussion/Limitations to address, in addition to the lack of treatment outcome data, several methodological sources of uncertainty:

**Preprocessing dependence.** We now explicitly note that choices such as nuisance regression (including head motion), parcellation atlas, and graph-construction thresholds can influence functional connectivity estimates and downstream performance/subtyping. We emphasize that fully standardized pipelines and harmonization remain critical for translation.

**Site variability and acquisition heterogeneity.** We added a clearer discussion of scanner/protocol differences across sites and how these may affect connectivity and generalization. We also highlight that LOSO-CV and cross-cohort transfer mitigate but do not eliminate residual site effects.

**Hyperparameter and model-choice sensitivity.** We clarify that hyperparameters were tuned within each dataset and report the final settings (Table 2). We also acknowledge that different hyperparameter choices and architectural variants could yield different performance and subtype structure, and that broader benchmarking and prospective validation are needed.

*Reviewer #4:*

*This manuscript addresses an important and timely question in precision psychiatry: whether resting-state functional connectivity, analyzed through an ensemble graph neural network (EH-BrainGNN) with clustering, can improve classification and subtyping of major depressive disorder (MDD). The work has notable strengths, including use of large multi-site datasets (REST-meta-MDD, N=1604; SRPBS, N=446), validation across Chinese and Japanese cohorts, and the attempt to identify reproducible biological subtypes of MDD. However, the manuscript has significant weaknesses in presentation, methodological transparency, statistical analysis, and interpretation. These must be addressed before the paper could be considered for publication in a plos dogital health journal.*

*1. Clarity and Grammar*

*The manuscript has many grammatical errors, awkward phrases and typos that impede readability. Examples include:*

*• The duplicate “the” in the Methods: “Standard preprocessing … were done using the the*

*Data Processing Assistant for Resting-State fMRI (DPARSF)”.*

*• An ungrammatical phrase “In regarding to the unsupervised subtyping model”; this*

*should read “Regarding the unsupervised subtyping model…”.*

*• Sentences with dangling modifiers or missing articles, e.g., “To mitigate distinct sites,*

*artifact or confounding effect, we adopted ComBat harmonization method” (should be*

*“**To mitigate site differences and confounding effects…”).*

*• Typographical errors such as “reedifying” instead of “**redefining” in the Discussion, and*

*“response” instead of “respond”.*

*• In the Discussion, “different biological mechanisms underline different subtypes” (should*

*be “**underlie”) and “the importance of identifying diagnostic biomarker for MDD subtypes have long been recognized, by the solution is still unclear” (missing conjunction).*

*• Several run-on sentences, such as the claim that “EH-BrainGNN achieved the highest*

*average generalization performance of 73% in most sites compared with other baselines*

*and yielded significant and consistent improvements, suggesting an advantage of our*

*study that the EH-BrainGNN was able to generalize well on the completely different*

*multi-sites even if site-specific difference”.*

*• Use of the verb “access” instead of “assess” when stating future plans: “Apply rTMS to*

*different subtypes and access the treatment efficacy”.*

*• The abstract and title contain minor spacing errors (“majordepression” and“networkapproach”).*

*Recommendation: Engage a professional editor or a native English speaker to proofread and*

*revise the manuscript. Ensure subject–verb agreement, correct tense usage, and proper word*

*choice throughout.*

**Response:** Corrected as suggested.

*2. Methodological Details and Justification*

*The study uses an ensemble hybrid framework (EH-BrainGNN) combining a supervised graph*

*neural network with unsupervised clustering, but several methodological choices lack justification or clarity.*

*1. Data description and cohort differences:*

*o The paper states the principal dataset contains 1604 participants (848 MDD, 794 controls) from 17 sites and the independent validation dataset contains 446 participants (177 MDD, 269 controls) from 4 sites. However, there is little discussion of differences in demographics, scanning parameters, or cultural factors between Chinese and Japanese sites that could influence functional connectivity. How were these variables controlled beyond ComBat harmonization?*

**Response:** We thank the reviewer for this important point. We agree that demographic differences, acquisition heterogeneity, and population or cultural factors could influence resting-state functional connectivity and thereby affect cross-cohort generalization. Beyond ComBat harmonization (applied when covariates were available), we used several additional controls and evaluation strategies, which we now clarify in the revised manuscript.

**Consistent preprocessing and graph construction.** Both cohorts were processed using the same standardized preprocessing pipeline and identical graph-construction procedure (parcellation, nuisance regression, and connectivity estimation), to reduce methodological variability across datasets.

**Covariate handling and reporting.** Where demographic and quality-control covariates were available, we included them in ComBat (principal dataset: age, sex, education, and head motion; independent dataset: age, sex, and head motion due to the lack of education metadata). We now explicitly report cohort-level demographic summaries and acquisition parameters (scanner and key sequence settings, where provided) in the Supplementary Materials.

**Site-aware generalization evaluation.** Most importantly, we evaluated robustness using leave-one-site-out cross-validation and cross-national transfer, which explicitly test whether the learned representations generalize to unseen sites and a distinct population. These settings reduce the likelihood that performance is driven by site-specific acquisition or cohort-specific characteristics.

**Limitations and future directions.** We acknowledge that residual confounds may remain, particularly for factors not captured in available metadata (e.g., cultural/environmental influences). We therefore temper claims about cross-population biological equivalence and emphasize the need for future prospective studies with harmonized phenotyping and standardized acquisition, as well as richer covariate modeling (e.g., mixed-effects or domain-adaptation approaches) to further disentangle these effects.

*2. ComBat harmonization:*

*o ComBat is used to control site effects, but the description is copied from the original formula and lacks clarity (equations (1)–(2) are presented without explaining all variables). There is also a grammatical error “using uses Empirical Bayes”. The authors need to explain why ComBat is appropriate, whether other harmonization methods were considered, and provide evidence that it does not remove biological variation relevant to MDD.*

**Response:** Thank you for raising these important concerns. In the revised manuscript, we have added analysis to demonstrate that ComBat reduces site-related variability without removing biologically meaningful signal.

**Biological-signal preservation after ComBat.**
To verify that harmonization did not inadvertently remove disease-relevant effects, we performed a **case–control diagnostic** by comparing MDD versus HC functional connectivity before and after ComBat. Specifically, we computed edge-wise effect sizes (**Cohen’s** $d$) for all FC edges and visualized them as ROI×ROI heatmaps for the raw FC and the ComBat-adjusted FC. As shown in **Supplementary Fig. 6**, the spatial distribution and overall pattern of case–control differences were highly similar before and after harmonization, indicating preservation of the biological signal. Moreover, the change in effect sizes was minimal: the absolute difference $\left| \Delta d \right|$ between pre- and post-ComBat estimates was very small across edges, with a maximum of only **0.08**, further supporting that ComBat did not substantially attenuate disease-related variability.

**Supplementary Figure 6 Cohen’s** $\boldsymbol{d}$ **heatmaps for the raw FC and the ComBat-adjusted FC.**

*3. Graph construction:*

*o The FC matrix is binarized by setting the top 20-percentile absolute correlation values to one. This threshold appears arbitrary. How robust are the results to different thresholds (e.g., 10%, 15%, weighted networks)? A sensitivity analysis is needed.*

**Response:** We thank the reviewer for raising this important point. In the revised manuscript, we now explicitly justify our choice of a 20% binarization threshold and assess its impact on both predictive performance and subtype discovery.

First, we note that percentile-based binarization in the range of 10–30% has been commonly adopted in related work to balance sparsity with sufficient signal retention in high-dimensional biological data, and we now cite these studies in the Methods section (refs. [1-3]). Motivated by this practice, we originally selected 20% as a moderate cutoff that avoids overly sparse representations while still emphasizing the most informative features. To empirically evaluate this choice, we performed an ablation study over five candidate thresholds (10%, 15%, 20%, 25% and 30%) under all three evaluation schemes used in this work: (i) 10-fold cross-validation, (ii) leave-one-site-out cross-validation, and (iii) training on the principal dataset with testing on the independent validation cohort. The results are summarized in Supplementary Figure 7 and Supplementary Tables 5–6. Across all three schemes, the 20% threshold consistently achieved the highest or near-highest mean AUC, with only modest fluctuations in performance at neighboring thresholds.

**Supplementary Figure 7 The impact of graph construction threshold on 10-fold cross-validation.**

**Supplementary Table 5 The impact of graph construction threshold on leave-one-site cross-validation.**

| **Threshold** | **10%** | **15%** | **20%** | **25%** | **30%** |
| --- | --- | --- | --- | --- | --- |
| **Site1** | 0.56 | 0.55 | **0.63** | **0.63** | **0.63** |
| **Site2** | 0.73 | 0.70 | **0.80** | 0.67 | 0.67 |
| **Site3** | 0.80 | 0.73 | **0.88** | 0.71 | 0.71 |
| **Site4** | 0.72 | 0.71 | **0.75** | 0.69 | 0.68 |
| **Site5** | 0.67 | 0.68 | **0.70** | 0.64 | 0.67 |
| **Site6** | 0.64 | 0.63 | **0.69** | 0.68 | 0.63 |
| **Site7** | 0.72 | 0.66 | **0.75** | 0.65 | 0.68 |
| **Site8** | **0.73** | **0.73** | 0.70 | 0.65 | 0.62 |
| **Site9** | **0.78** | 0.75 | **0.78** | **0.78** | **0.78** |
| **Site10** | 0.68 | 0.69 | **0.72** | 0.71 | 0.67 |
| **Site11** | 0.82 | 0.81 | 0.84 | **0.85** | 0.84 |
| **Site12** | 0.62 | 0.61 | **0.67** | 0.61 | 0.65 |
| **Site13** | 0.65 | 0.69 | **0.73** | 0.69 | 0.65 |
| **Site14** | 0.60 | 0.59 | **0.63** | 0.59 | 0.60 |
| **Site15** | 0.55 | 0.59 | **0.68** | 0.59 | 0.61 |
| **Site16** | 0.61 | 0.53 | **0.76** | 0.59 | 0.63 |
| **Site17** | 0.64 | 0.64 | 0.64 | **0.73** | **0.73** |
| **Mean** | 0.68 | 0.66 | **0.73** | 0.67 | 0.67 |

**Supplementary Table 6 The impact of graph construction threshold on generalization performance in the independent validation dataset using the principal dataset.**

| **Threshold** | **10%** | **15%** | **20%** | **25%** | **30%** |
| --- | --- | --- | --- | --- | --- |
| **HUH** | 0.65 | 0.65 | **0.73** | 0.66 | 0.63 |
| **HRC** | 0.72 | 0.74 | 0.74 | **0.75** | **0.75** |
| **HKH** | 0.63 | 0.60 | **0.66** | 0.63 | **0.66** |
| **COI** | 0.64 | 0.62 | **0.66** | 0.65 | 0.60 |
| **Mean** | 0.66 | 0.65 | **0.70** | 0.67 | 0.66 |

We further evaluated the influence of the binarization threshold on subtype discovery. Although individual sample assignments exhibited minor perturbations at the lowest and highest thresholds, the overall subtype architecture and the defining biological signatures of each subtype were conserved across the 10–30% range. In this context, the 20% threshold yielded a favorable compromise, providing highly stable subtype solutions with clear inter-subtype separation and consistently strong predictive performance.

[1] CI-GNN: A Granger Causality-Inspired Graph Neural Network for Interpretable Brain Network-Based Psychiatric Diagnosis. Neural Networks, 2024.

[2] BrainIB: Interpretable Brain Network-Based Psychiatric Diagnosis With Graph Information Bottleneck. IEEE Transactions on Neural Networks and Learning Systems, 2025.

[3] BPI-GNN: Interpretable brain network-based psychiatric diagnosis and subtyping. Neuroimage, 2024.

*4. Feature representation:*

*o Node features are defined as the correlation of each ROI with all other ROIs. This leads to high dimensionality; were there any feature reduction steps? Also, the authors refer to “subgraph generator” with an edge mask learned via the Concrete distribution but do not state how this ensures interpretability or whether the learned mask is consistent across folds.*

**Response:** We thank the reviewer for these questions.

**High-dimensional node features and feature reduction.**
In our graph construction, each node feature vector $X_{k} = [\rho_{k1}, . . . ,\rho_{kn}]T$ has dimensionality $\boldsymbol{n}$ (the number of ROIs). We did not apply an explicit handcrafted feature-reduction step (e.g., PCA) prior to modeling. Instead, dimensionality reduction is performed implicitly by the learnable feature encoder in the GNN: the node features are projected to a lower-dimensional latent space through the first GNN layer and subsequent message-passing layers, and the final classifier operates on a compact graph-level embedding. We have clarified this point in the revised Methods. We also note that $n$ is moderate in our setting (e.g., AAL: $n=116$), making the input dimensionality tractable.

**Interpretability and cross-fold consistency of the subgraph generator**

To quantify the robustness of the subtype-associated edges, we performed 1000 bootstrap resampling of subjects. For each resample, we recomputed the subject-level top-20 edges and aggregated them within each subtype to obtain resampled subtype-level top-20 sets, which were then compared with the reference set using the Jaccard index. The corresponding results are shown in Supplementary Figure S4.

As shown in Supplementary Fig. S4 A, the subtype-specific edge sets exhibited moderate-to-high stability across resamples: the mean Jaccard overlap was 0.433 (95% CI [0.250, 0.600]) for subtype 1, 0.405 (95% CI [0.176, 0.667]) for subtype 2, and 0.335 (95% CI [0.143, 0.538]) for subtype 3. Because both the reference and bootstrap-derived sets contain 20 edges, these values correspond to approximately 10–12 shared edges on average, indicating that more than half of the reported edges are consistently recovered across resamples, while the remaining edges form a more variable peripheral set. Notably, these overlaps are far above chance, as random selection of 20 edges from the full edge set yields Jaccard values close to zero, supporting the presence of a reproducible core of subtype-specific connections. Moreover, for each subtype, a substantial fraction of the reference top-20 edges showed high bootstrap selection frequencies (>0.5), with 19 of 20 edges in subtype 1, 16 of 20 in subtype 2, and 9 of 20 in subtype 3 exceeding this threshold, further highlighting a robust core set of connections (Supplementary Fig. S4 B–D).

**Supplementary Figure S4. The stability of the subtype-specific top-20 edges.** (A) the stability of the subtype-specific top-20 edges across bootstrap resamples quantified by the Jaccard index. (B) the bootstrap selection frequency of the reference top-20 edges for each subtype.

*5. Mutual information estimator:*

*o The mutual information loss functions (equations (9)–(12)) are presented but not well explained. Key hyperparameters (λ1, λ2, λ3) controlling sparsity and compactness are not specified in the main text.*

**Response:** Thank you for this important comment.

**1) Clarifying the mutual-information objectives (Eqs. 9–12).**
In the revised manuscript, we added a step-by-step explanation of the roles of each mutual-information term, emphasizing how they contribute to (i) learning compact, subtype-consistent representations and (ii) encouraging sparsity/interpretability while maintaining discriminative power. We also provide an intuitive description of how these objectives interact with the supervised prediction loss in the overall optimization.

**2) Reporting the key hyperparameters λ1 , λ2, and λ3**
We also addressed the stability of the framework with respect to the multi-term objective by conducting an ablation study in the cross-national validation setting (training on the principal cohort and testing on the independent cohort). As summarized in Supplementary Table 7, removing any individual loss component led to a marked degradation in performance, demonstrating that each term plays a substantial role in balancing sparsity with predictive accuracy and improving generalization. These results support the robustness of the proposed objective and justify the inclusion of all loss components in the final model.

**Supplementary Table 7 Ablation study of loss components (cross-national validation).**

| $L_{mask}$ | $L_{sps}$ | $L_{ent}$ | Accuracy |
| --- | --- | --- | --- |
| 🗴 | 🗴 | 🗴 | 0.61 |
| 🗸 | 🗴 | 🗴 | 0.67 |
| 🗴 | 🗸 | 🗴 | 0.61 |
| 🗴 | 🗴 | 🗸 | 0.64 |
| 🗸 | 🗸 | 🗴 | 0.63 |
| 🗴 | 🗸 | 🗸 | 0.61 |
| 🗸 | 🗴 | 🗸 | 0.66 |
| 🗸 | 🗸 | 🗸 | 0.73 |

*6. Training and hyperparameter selection:*

*The paper mentions using 10-fold cross-validation with an Adam optimizer, dropout and weight decay. However, the grid search space for hyperparameters is only referred to in Table 2 (not provided in the manuscript excerpt). Without detailed hyperparameter ranges and final values, reproducibility is limited.*

**Response:** We thank the reviewer for this constructive comment. In the revised manuscript, we have substantially expanded the subsection “Training, testing and hyperparameter optimization” to provide a precise and reproducible description of our model selection procedure.

Specifically, we now clarify that all hyperparameters were tuned using a nested cross-validation scheme. For each experiment, we performed an outer 10-fold cross-validation. Within each outer training split, we conducted an inner 5-fold cross-validation to optimize hyperparameters. For every candidate hyperparameter configuration defined in Table 2 (including learning rate, batch size, number of layers, number of hidden units, weight decay, dropout rate, and other model-specific parameters), the model was trained on 4 inner folds and evaluated on the remaining fold, cycling through all inner folds. We then selected the configuration that maximized the mean accuracy across the inner validation folds. After identifying the best hyperparameter configuration for a given outer fold, we retrained the model on the full outer training set using this fixed configuration. Early stopping was applied with a patience of 10 epochs, monitoring the validation accuracy computed on an internal validation split drawn exclusively from the outer training data. Importantly, neither the outer test folds nor the independent validation cohort were used at any stage for hyperparameter tuning, model selection, or calibration of early-stopping criteria. All hyperparameters and early-stopping rules were therefore fixed before scoring any outer test fold or the independent cohort, ensuring unbiased performance estimates.

We have also revised Table 2 to explicitly describe the hyperparameter grid explored in the inner-loop search and to report, for each experiment, the final selected values. In addition, we now provide code-level details sufficient to reproduce the search procedure and the corresponding implementation is provided in our public GitHub repository at https://github.com/ZKZ-Brain/EH-BrainGNN.

*7. Baseline comparisons:*

*The authors compare EH-BrainGNN with SVMs, LASSO, Random Forest, and several GNN variants. Yet, the paper does not describe how these models were tuned or whether the training/test splits were identical. Statistical tests comparing performance (e.g., McNemar test, paired t-test on fold results) are not reported.*

**Response:** Thanks. In the revised manuscript, we have compared EH-BrainGNN against the next-best method using paired tests across the 10 cross-validation folds (fold-wise ACC as paired observations). For the Principal Dataset, EH-BrainGNN is compared to BrainIB in terms of ACC: *t* = 4.3529, *p* = 0.0018, Cohen’s *d* = 1.3765 with bootstrap 95% CI [0.7998, 2.9158]. For the Independent validation dataset, EH-BrainGNN is compared to CI-GNN (ACC): *t*= 3.1623, *p* = 0.0115, Cohen’s d = 1.0000 with bootstrap 95% CI [0.4454, 2.4244].

*8. Class imbalance:*

*With 848 MDD and 794 controls in the training set, class balance is acceptable, but the validation set has 177 MDD and 269 controls. Did the authors use balanced accuracy or F1-scores to account for imbalance? They report only accuracy and AUC.*

**Response:** Thanks. To address class imbalance and site heterogeneity, we now report sensitivity and F1-score alongside ACC and AUC for the generalization evaluation on the independent validation dataset (trained on the principal dataset), providing a more informative assessment under potential class imbalance.

**Table 5. Generalization performance on different models in the independent validation dataset using the principal dataset.** The highest performance is highlighted with boldface.

| Sites  Method | HUH | | | | HRC | | | | HKH | | | | COI | | | |
| --- | --- | --- | --- | --- | --- | --- | --- | --- | --- | --- | --- | --- | --- | --- | --- | --- |
|  | ACC | AUC | Sen | F1 | ACC | AUC | Sen | F1 | ACC | AUC | Sen | F1 | ACC | AUC | Sen | F1 |
| LASSO | 0.59 | 0.60 | 0.51 | 0.58 | 0.43 | 0.58 | 0.27 | 0.41 | 0.50 | 0.50 | 0.48 | 0.48 | 0.57 | 0.56 | 0.66 | 0.66 |
| RF | 0.47 | 0.54 | 0.41 | 0.47 | 0.34 | 0.46 | 0.16 | 0.27 | 0.61 | 0.61 | 0.55 | 0.58 | 0.48 | 0.56 | 0.41 | 0.50 |
| L-SVM | 0.58 | 0.59 | 0.74 | 0.67 | 0.49 | 0.53 | 0.39 | 0.52 | 0.48 | 0.49 | 0.24 | 0.30 | 0.56 | 0.59 | 0.65 | 0.65 |
| RBF-SVM | 0.61 | 0.61 | 0.59 | 0.62 | 0.42 | 0.55 | 0.35 | 0.47 | 0.45 | 0.46 | 0.21 | 0.27 | 0.63 | **0.63** | 0.67 | 0.71 |
| GCN | 0.67 | 0.68 | 0.69 | 0.70 | 0.62 | 0.52 | 0.69 | 0.72 | 0.57 | 0.58 | 0.52 | 0.55 | 0.56 | 0.53 | 0.62 | 0.64 |
| GAT | 0.66 | 0.67 | 0.59 | 0.66 | 0.60 | 0.58 | 0.65 | 0.71 | 0.61 | 0.61 | 0.34 | 0.45 | 0.63 | 0.58 | 0.70 | 0.71 |
| GIN | 0.45 | 0.48 | 0.03 | 0.05 | 0.61 | 0.61 | 0.59 | 0.70 | 0.53 | 0.53 | 0.66 | 0.55 | 0.55 | 0.52 | 0.62 | 0.64 |
| DIR-GNN | 0.66 | 0.67 | 0.75 | 0.71 | 0.61 | 0.61 | 0.59 | 0.68 | 0.62 | 0.64 | 0.55 | 0.59 | 0.59 | 0.59 | 0.56 | 0.62 |
| ProtGNN | 0.62 | 0.62 | 0.56 | 0.62 | 0.61 | 0.59 | 0.65 | 0.72 | 0.61 | 0.61 | 0.55 | 0.58 | 0.53 | 0.55 | 0.49 | 0.57 |
| BrainGNN | 0.58 | 0.59 | 0.63 | 0.63 | 0.68 | 0.57 | 0.78 | 0.76 | 0.52 | 0.53 | 0.48 | 0.49 | 0.61 | 0.50 | 0.64 | 0.67 |
| CI-GNN | 0.61 | 0.61 | 0.62 | 0.63 | 0.69 | 0.58 | 0.78 | 0.77 | 0.65 | 0.65 | 0.62 | 0.62 | 0.58 | 0.57 | 0.58 | 0.63 |
| BrainIB | 0.52 | 0.71 | 0.39 | 0.64 | 0.53 | 0.63 | 0.59 | 0.70 | 0.53 | 0.52 | 0.52 | 0.52 | 0.63 | 0.58 | **0.86** | **0.74** |
| EH-BrainGNN | **0.73** | **0.72** | **0.79** | **0.76** | **0.74** | **0.62** | **0.86** | **0.83** | **0.66** | **0.67** | **0.72** | **0.67** | **0.66** | **0.63** | 0.75 | **0.74** |

*9. Cross-site validation:*

*The leave-one-site-out analysis across 17 sites is appropriate, but the authors should provide standard errors or confidence intervals for the 73% average accuracy. They also claim the highest performance is 88% at one site without identifying the site; such details are necessary.*

**Response:** We thank the reviewer for this important suggestion. We agree that uncertainty estimates are needed for the leave-one-site-out (LOSO) results and that site identifiers should be reported for transparency.

- **Uncertainty for the LOSO average accuracy**

In the revised manuscript, we now report the mean LOSO accuracy together with uncertainty, including the standard error across the 17 held-out sites and the corresponding 95% confidence interval (computed from the site-wise LOSO accuracies). We include these values in the Results.

- **Identifying the highest-performing site**

We also now explicitly identify the site corresponding to the highest reported accuracy (88%) and provide the full site-wise performance table (accuracy and additional metrics) for all 17 sites in the Supplementary Materials. This revision ensures that readers can directly assess site-level variability and reproduce the reported summaries

*10. Unsupervised clustering:*

*o The CFDP algorithm automatically selects the number of clusters. The paper reports three subtypes representing 25.9%, 62.1% and 12.1% of patients. However, cluster stability is not assessed (e.g., using bootstrapping or consensus clustering). It is unclear whether the same patients fall into the same subtype across folds. The authors also do not report silhouette scores or other metrics to support three clusters.*

**Response: Response:** We thank the reviewer for this helpful suggestion. In the revised manuscript, we have substantially clarified how cluster centers and the number of clusters are determined and have added both visual and quantitative evidence supporting the choice of $K=3$ in both datasets.

First, we now explicitly describe the rules used with CFDP (clustering by fast search and find of density peaks) to select cluster centers from the decision graph. For each subject, CFDP computes a local density $\rho_{i}$ and a distance to the nearest point with higher density $\delta_{i}$. We plot $\delta_{i}$ against $\rho_{i}$ to obtain the decision graph and define a combined prominence score:

$$\gamma_{i}=\rho_{i}\times\delta_{i}.$$

Points with large values of both $\rho_{i}$ and $\delta_{i}$ correspond to high $\gamma_{i}$ and are treated as candidate cluster centers. For each dataset, we visualize the decision graph, identify the prominent corner region with high density and large distance, and select as centers those points with the largest $\gamma_{i}$. As shown in the decision graphs (Figure 5 A and C) and corresponding spatial distributions (Figure 5 B and D) that we now include for both datasets, three such prominent peaks are clearly present, leading to three CFDP centers in each case.

Second, in accordance with the reviewer’s request, we have added cluster quality analyses across a range of candidate values of $K$. Using the learned embeddings and the corresponding CFDP assignments, we compute the silhouette coefficient and Dunn index for different choices of $K$. In both datasets, $K=3$ either attains the highest silhouette coefficient or lies very close to the optimum, and it is also associated with higher Dunn values compared with neighboring values of $K$ (Figure 5 E-H). These results are now reported in the Results and illustrated in Figure 5 E-H that shows the silhouette and Dunn indices as functions of $K$, where three clusters emerge as the most parsimonious and well supported solution.

**Figure 5. Determination of the optimal number of CFDP subtypes.** (A-B) CFDP decision graphs for the two datasets. (C-D) Two dimensional embeddings illustrating the spatial distribution of subjects colored by CFDP subtype. (E-F) Silhouette coefficients as a function of the number of clusters ($K=2-9$). (G-H) Dunn indices for $K=2-9$.

*11. Statistical analysis of subtypes:*

*Wilcoxon rank-sum tests with FDR correction are used to compare HAMD symptoms across subtypes. The manuscript does not specify the number of tests performed (nine HAMD items?) and the correction method (Benjamini–Hochberg?). Additionally, p=0.066 is reported as a “reduced pattern” for BDI scores, which is not statistically significant.*

**Response:** We thank the reviewer for pointing this out. We have clarified the multiple-testing details in the revised Methods. Specifically, symptom differences across subtypes were assessed using Wilcoxon rank-sum tests, and we performed m = 9 HAMD item-wise tests (one per item). To control for multiple comparisons, we applied the Benjamini–Hochberg (BH) false discovery rate (FDR) procedure within this family of tests, using a preset threshold of *q* < 0.05. We now report both the raw P values and the corresponding BH-adjusted *q*-values in the revised manuscript/Supplementary Table. We also agree that *p* = 0.066 for the BDI comparison should not be described as statistically significant. In the revision, we have removed any language implying significance.

*12. Interpretability claims:*

*o The authors describe EH-BrainGNN as “self-explaining” and claim that the learned subgraphs provide interpretability. Yet there is no quantitative assessment of interpretability (e.g., overlap with known depression networks, comparison with expert-defined ROIs) or demonstration of how clinicians might use these explanations.*

**Response:** We have expanded the Discussion to clarify how the subtype-specific connectivity patterns identified by our framework can be viewed as candidate circuit-level biomarkers with potential implications for precision treatment in MDD.

First, our clustering of learned embeddings revealed three reproducible MDD subtypes characterized by distinct connectivity motifs: (i) a default mode network–cerebellum subtype, (ii) an insula–cingulum–temporal subtype, and (iii) a frontostriatal subtype. Because our edge-level explanations are derived directly from the predictive model, these subtype-specific patterns provide an interpretable link between latent representations and biologically meaningful circuits, rather than abstract, model-internal features.

Second, we now discuss how these circuit signatures may inform personalized target selection for neuromodulation, particularly in the context of rTMS, where current practice in MDD largely relies on “one-size-fits-all” dorsolateral prefrontal cortex (DLPFC) targeting and lacks principled strategies for individualized target choice. Our results suggest a testable precision-medicine framework: patients assigned to the default mode network–cerebellum subtype might benefit from cerebellar stimulation as a candidate target; those in the insula–cingulum–temporal subtype might be preferentially considered for insula- or salience-network–oriented targeting; and those in the frontostriatal subtype align more closely with conventional DLPFC-based stimulation protocols. We explicitly note in the revised Discussion that these implications are hypothesis-generating rather than prescriptive, and that rigorous prospective interventional trials would be required to determine whether subtype-guided rTMS targeting indeed improves treatment response.

Finally, we emphasize that the edge-level biomarkers identified by our explainable framework are (i) sparse, (ii) circuit-specific, and (iii) reproducible across resampling and permutation tests, properties that are desirable for translational biomarkers intended for clinical deployment. We have added text to highlight how this combination of interpretability and robustness moves beyond purely predictive models and points towards a mechanistically grounded, subtype-informed approach to biomarker development in depression.

*13. Reproducibility and code availability:*

*o The paper states that data from the REST-meta-MDD and SRPBS datasets are publicly available, but there is no statement about code availability. For a deep-learning study, sharing code and trained models is essential for reproducibility.*

**Response:** We thank the reviewer for highlighting this point. We agree that code availability is essential for reproducibility. We have now added a clear Code Availability statement in the revised manuscript, and we have released the implementation (including code, and configuration files) on GitHub: https://github.com/ZKZ-Brain/EH-BrainGNN.

*Recommendation: Provide comprehensive methodological details, justify key choices (e.g.,*

*thresholding, hyperparameters), add robustness analyses, report statistical tests for baseline*

*comparisons, and make code publicly available.*

*3. Results Presentation*

*• Many results are described without corresponding figures or tables in the main text (e.g.,*

*Table 3, Figures 3–5, Table 5). For a reader to judge the claims, these visuals must be accessible.*

**Response:** We thank the reviewer for pointing this out and agree that the current presentation makes it difficult for readers to evaluate the claims. We have revised the manuscript to ensure that all results discussed in the main text are accompanied by accessible figures or tables. Specifically, we have reorganized the Results section, moved key visual evidence into the main manuscript, and standardized the numbering and cross-references so that every citation (e.g., Tables 3 and 5; Figs. 3–5) points to the correct item in the revised submission. Additional or secondary analyses are now clearly labeled and placed in the Supplementary Materials, with explicit in-text pointers.

*• The text repeatedly refers to accuracy alone; sensitivity, specificity, precision, recall and*

*F1-score should be reported. The authors also need to clarify whether 74%/73% accuracy*

*is sufficiently high for clinical translation.*

**Response:** We thank the reviewer for this important suggestion. We agree that reporting accuracy alone is insufficient, particularly under potential class imbalance and for clinically oriented interpretation. In the revised manuscript, we now report a more complete set of classification metrics, including sensitivity, and F1-score (in addition to ACC/AUC).

We also agree that an accuracy of ~0.73–0.74 should not be presented as ready for clinical deployment. We have revised the Discussion to adopt a more cautious tone, explicitly stating that the current performance is encouraging for cross-site/cross-country generalization, but not sufficient for clinical translation without further prospective multi-site validation, calibration and threshold selection for specific clinical use cases, and assessment of failure modes and subgroup fairness.

*• When reporting p-values, specify whether tests are one-sided or two-sided and include effect sizes.*

**Response:** Corrected as suggested.

*4. Discussion and Interpretation*

*• The Discussion repeats results but provides limited clinical context. For example, how might the three subtypes inform personalized treatment or prognosis? Are there known symptom clusters or biological markers that align with these subtypes? How do the findings relate to previous work by Drysdale et al. or Wang et al.? A more comprehensive comparison with existing subtyping frameworks is needed.*

**Response:** We thank the reviewer for this helpful suggestion and agree that the Discussion should provide clearer clinical context and a more explicit comparison to prior subtyping frameworks. We have revised the Discussion accordingly in three ways.

First, we now clarify how the three connectivity defined subtypes could hypothesis generate clinically relevant stratification. In particular, the subtype signatures we observe implicate circuits that have been repeatedly linked to depressive symptom dimensions, including default mode network related processes (for example, self-referential or ruminative cognition), insula and cingulate related salience and affect regulation, and frontostriatal circuitry related to reward and motivation. We emphasize that, because treatment response and longitudinal outcomes are not available in these public datasets, these links should be interpreted as clinically motivated hypotheses rather than validated predictors.

Second, we add a more comprehensive comparison with existing subtyping work. We explicitly discuss how our subtype patterns relate to prior connectivity based “biotype” frameworks, including Drysdale et al., who reported four resting state connectivity defined biotypes involving frontostriatal and limbic networks and showed prognostic relevance for neurostimulation response. We also compare our approach to other data driven frameworks that combine connectivity and symptom information and report different subtype solutions (for example, two subtype solutions with distinct symptom profiles such as insomnia dominated versus anhedonia dominated patterns), highlighting that the number of subtypes can vary with features, cohort composition, and clustering methodology.

Third, we strengthen the cautionary framing on replication and method dependence. We now explicitly acknowledge that subtype solutions can be sensitive to analytic choices and sampling, and we therefore emphasize the need for replication in additional multisite cohorts and prospective studies, consistent with prior methodological discussions in the literature.

*• The authors acknowledge that treatment response could not be assessed because only baseline data were available. This limitation should be emphasized earlier in the abstract and conclusions.*

**Response:** We thank the reviewer for this important suggestion and agree that this limitation should be more visible. We have revised both the Abstract and the Conclusion to state explicitly that the analyzed cohorts provide baseline rs-fMRI only, and therefore treatment response, prognosis, and longitudinal outcomes could not be evaluated in this study. We also temper the language around clinical utility accordingly and emphasize that prospective studies with outcome data are required to test whether the identified subtypes predict treatment response.

*• The potential impact of cultural differences between Chinese and Japanese cohorts is not*

*discussed. Differences in diet, lifestyle, or clinical practice may influence brain connectivity and should be considered when generalizing results.*

**Response:** We thank the reviewer for raising this important point. We agree that cultural and population level differences between the Chinese and Japanese cohorts, including lifestyle factors such as diet, sleep, and physical activity, as well as environmental exposures and clinical practice patterns, could influence resting state functional connectivity and may affect cross cohort generalization.

We have added an explicit discussion of this issue in the revised Discussion. Specifically, we note that such factors are not captured in the available public metadata and therefore cannot be explicitly controlled in the present retrospective analysis. We accordingly temper claims about cross-population biological equivalence and emphasize that our cross-national evaluation should be interpreted primarily as a stringent test of robustness under real-world distribution shifts, rather than proof that the learned signatures are invariant to cultural context. We further highlight the need for future prospective multi-country studies with harmonized phenotyping and richer covariate collection to disentangle cultural, clinical, and biological sources of variability.

*• Some statements are overly strong, e.g., claiming that the classifier provides a “clear diagnosis boundary”. Given the modest accuracy (73%), such claims should be tempered.*

**Response:** We thank the reviewer for this important point and agree that the phrase “clear diagnosis boundary” is too strong given the current performance. We have rephrased this wording throughout the manuscript to adopt a more cautious and accurate description.

*5. Ethical and Reporting Considerations*

*• The paper mentions that all participants provided informed consent and lists funding sources. However, there is no description of ethics approval numbers or which institutional review boards approved the study.*

*• Competing interests are declared but need to follow the standard BMC format.*

*• Figures and tables are referenced but not included in the draft; ensure all supplementary*

*materials are properly linked.*

**Response:** Corrected as suggested.

*6. Specific Questions*

*1. Why was the binarization threshold set at the top 20% of absolute FC values? Did you test other thresholds or weighted adjacency matrices?*

**Response:** We thank the reviewer for raising this important point. In the revised manuscript, we now explicitly justify our choice of a 20% binarization threshold and assess its impact on both predictive performance and subtype discovery.

First, we note that percentile-based binarization in the range of 10–30% has been commonly adopted in related work to balance sparsity with sufficient signal retention in high-dimensional biological data, and we now cite these studies in the Methods section (refs. [1-3]). Motivated by this practice, we originally selected 20% as a moderate cutoff that avoids overly sparse representations while still emphasizing the most informative features. To empirically evaluate this choice, we performed an ablation study over five candidate thresholds (10%, 15%, 20%, 25% and 30%) under all three evaluation schemes used in this work: (i) 10-fold cross-validation, (ii) leave-one-site-out cross-validation, and (iii) training on the principal dataset with testing on the independent validation cohort. The results are summarized in Supplementary Figure 7 and Supplementary Tables 5–6. Across all three schemes, the 20% threshold consistently achieved the highest or near-highest mean AUC, with only modest fluctuations in performance at neighboring thresholds.

**Supplementary Figure 7 The impact of graph construction threshold on 10-fold cross-validation.**

**Supplementary Table 5 The impact of graph construction threshold on leave-one-site cross-validation.**

| **Threshold** | **10%** | **15%** | **20%** | **25%** | **30%** |
| --- | --- | --- | --- | --- | --- |
| **Site1** | 0.56 | 0.55 | **0.63** | **0.63** | **0.63** |
| **Site2** | 0.73 | 0.70 | **0.80** | 0.67 | 0.67 |
| **Site3** | 0.80 | 0.73 | **0.88** | 0.71 | 0.71 |
| **Site4** | 0.72 | 0.71 | **0.75** | 0.69 | 0.68 |
| **Site5** | 0.67 | 0.68 | **0.70** | 0.64 | 0.67 |
| **Site6** | 0.64 | 0.63 | **0.69** | 0.68 | 0.63 |
| **Site7** | 0.72 | 0.66 | **0.75** | 0.65 | 0.68 |
| **Site8** | **0.73** | **0.73** | 0.70 | 0.65 | 0.62 |
| **Site9** | **0.78** | 0.75 | **0.78** | **0.78** | **0.78** |
| **Site10** | 0.68 | 0.69 | **0.72** | 0.71 | 0.67 |
| **Site11** | 0.82 | 0.81 | 0.84 | **0.85** | 0.84 |
| **Site12** | 0.62 | 0.61 | **0.67** | 0.61 | 0.65 |
| **Site13** | 0.65 | 0.69 | **0.73** | 0.69 | 0.65 |
| **Site14** | 0.60 | 0.59 | **0.63** | 0.59 | 0.60 |
| **Site15** | 0.55 | 0.59 | **0.68** | 0.59 | 0.61 |
| **Site16** | 0.61 | 0.53 | **0.76** | 0.59 | 0.63 |
| **Site17** | 0.64 | 0.64 | 0.64 | **0.73** | **0.73** |
| **Mean** | 0.68 | 0.66 | **0.73** | 0.67 | 0.67 |

**Supplementary Table 6 The impact of graph construction threshold on generalization performance in the independent validation dataset using the principal dataset.**

| **Threshold** | **10%** | **15%** | **20%** | **25%** | **30%** |
| --- | --- | --- | --- | --- | --- |
| **HUH** | 0.65 | 0.65 | **0.73** | 0.66 | 0.63 |
| **HRC** | 0.72 | 0.74 | 0.74 | **0.75** | **0.75** |
| **HKH** | 0.63 | 0.60 | **0.66** | 0.63 | **0.66** |
| **COI** | 0.64 | 0.62 | **0.66** | 0.65 | 0.60 |
| **Mean** | 0.66 | 0.65 | **0.70** | 0.67 | 0.66 |

We further evaluated the influence of the binarization threshold on subtype discovery. Although individual sample assignments exhibited minor perturbations at the lowest and highest thresholds, the overall subtype architecture and the defining biological signatures of each subtype were conserved across the 10–30% range. In this context, the 20% threshold yielded a favorable compromise, providing highly stable subtype solutions with clear inter-subtype separation and consistently strong predictive performance.

[1] CI-GNN: A Granger Causality-Inspired Graph Neural Network for Interpretable Brain Network-Based Psychiatric Diagnosis. Neural Networks, 2024.

[2] BrainIB: Interpretable Brain Network-Based Psychiatric Diagnosis With Graph Information Bottleneck. IEEE Transactions on Neural Networks and Learning Systems, 2025.

[3] BPI-GNN: Interpretable brain network-based psychiatric diagnosis and subtyping. Neuroimage, 2024.

*2. How did you handle head motion beyond Friston’s 24-parameter model; were motion outliers or scrubbing applied?*

**Response:** Thank you for this clarification request. We used the publicly released REST-meta-MDD derivatives, for which the data providers implemented a standardized preprocessing pipeline adapted from DPARSF. In this pipeline, head motion is addressed using participant-level nuisance regression with the Friston-24 model, and the default analysis strategy also includes group-level correction for mean framewise displacement (FD).

Because we did not have access to (or reprocess) the raw fMRI time series within the consortium pipeline, we did not introduce additional preprocessing-stage motion handling beyond the provider’s DPARSF/Friston-24 regression (e.g., no additional volume censoring/scrubbing). Instead, to further mitigate any residual motion-related variance in our reanalysis, we included a mean FD as a covariate in ComBat harmonization and subsequent statistical analyses.

*3. Were there any site differences in MRI acquisition parameters (e.g., scanner type, TR/TE)? How might these affect functional connectivity and generalization?*

**Response:** We thank the reviewer for this important question. Yes—because our data were drawn from public multi-site cohorts, there are site differences in MRI acquisition parameters (e.g., scanner vendor/model and sequence settings such as TR/TE). Such differences can introduce systematic shifts in BOLD signal characteristics (e.g., temporal SNR and spectral content), which in turn may affect estimated functional connectivity and can reduce cross-site generalization if a model inadvertently learns site-specific signatures.

To address this, we took several steps in the revised manuscript:

**Documentation of acquisition heterogeneity.** We now summarize the available site-wise acquisition information (scanner and key sequence parameters, where provided by the dataset) in Supplementary Table [Sx].

**Mitigation in preprocessing/feature construction.** We applied a standardized preprocessing pipeline across sites (including nuisance regression and normalization steps) to reduce non-biological variance before graph construction.

**Generalization assessment under site shifts.** We evaluate robustness using leave-one-site-out cross-validation (LOSO-CV), which explicitly tests performance on a held-out site acquired under potentially different parameters, thereby reducing the likelihood that results are driven by site-specific acquisition characteristics.

**Limitations and future work.** We acknowledge that residual scanner/protocol effects may remain despite these controls, and that future work could further improve harmonization (e.g., with metadata-informed harmonization methods) and prospective standardized acquisition to strengthen clinical translation.

*4. The ComBat model includes covariates such as gender, age, and head motion. How did you verify that important biological variability was not removed by harmonization?*

**Response:** Thank you for raising these important concerns. In the revised manuscript, we have added analysis to demonstrate that ComBat reduces site-related variability without removing biologically meaningful signal.

**Biological-signal preservation after ComBat.**
To verify that harmonization did not inadvertently remove disease-relevant effects, we performed a **case–control diagnostic** by comparing MDD versus HC functional connectivity before and after ComBat. Specifically, we computed edge-wise effect sizes (**Cohen’s** $d$) for all FC edges and visualized them as ROI×ROI heatmaps for the raw FC and the ComBat-adjusted FC. As shown in **Supplementary Fig. 6**, the spatial distribution and overall pattern of case–control differences were highly similar before and after harmonization, indicating preservation of the biological signal. Moreover, the change in effect sizes was minimal: the absolute difference $\left| \Delta d \right|$ between pre- and post-ComBat estimates was very small across edges, with a maximum of only **0.08**, further supporting that ComBat did not substantially attenuate disease-related variability.

**Supplementary Figure 6 Cohen’s** $\boldsymbol{d}$ **heatmaps for the raw FC and the ComBat-adjusted FC.**

*5. How did you prevent data leakage when harmonizing the training and test sets in leave-one-site-out validation?*

**Response:** Thank you for this important question. We prevented data leakage by conducting ComBat harmonization within each leave-one-site-out (LOSO) fold, fitting the ComBat model using training sites only and never using any information from the held-out site to estimate harmonization parameters.

Specifically, for each LOSO iteration, we (i) held out one site as the test set; (ii) fit ComBat on the remaining sites (training set), including gender, age, education and head motion as covariates; and (iii) applied the resulting fold-specific ComBat parameters (site effects and pooled variance estimates) to harmonize the training data and then applied the same fitted transformation to the held-out site data. Thus, the test site did not contribute to the estimation of ComBat parameters, ensuring strict separation between training and testing.

*6. Did you examine whether demographic variables (age, sex, education) differed across the*

*identified subtypes?*

**Response:** Yes. We tested whether the identified subtypes differed in key demographic variables, including age, sex and education. We observed no significant between-subtype differences in age or education (one-way ANOVA) and no difference in sex distribution (χ² test). These findings indicate that the subtype structure is unlikely to be attributable to demographic imbalance.

*7. What was the rationale for choosing a Graph Isomorphism Network (GIN) encoder over other GNN architectures? Did you try graph convolutional networks (GCN) or graph attention networks (GAT)?*

**Response:** Thank you for this question. We selected the Graph Isomorphism Network (GIN) encoder based on both methodological considerations and empirical performance. GIN uses a sum-aggregation scheme with an MLP, which is known to provide strong discriminative capacity for graph-structured data and is well suited for capturing subtle topological differences in subject-level functional connectivity graphs.

Importantly, we benchmarked multiple GNN encoders under the same nested cross-validation protocol. In these comparisons, GIN consistently achieved the best performance, outperforming both GAT and GCN (mean accuracy: GIN > GAT (≈70%) > GCN (≈68%) under the same evaluation setting). Based on these results, we used GIN as the default encoder in the main experiments.

*8. The Concrete distribution requires a temperature parameter τ. How was this chosen, and*

*how sensitive are results to τ?*

**Response:** We thank the reviewer for this question. The temperature parameter τ in the Concrete (Gumbel–Sigmoid) relaxation controls the trade-off between **mask discreteness** and **gradient smoothness**: smaller τ produces near-binary edge masks but can lead to higher-variance/less stable gradients, whereas larger τ yields smoother masks but weaker sparsity.

In our implementation, we chose 𝜏 empirically on the training/validation splits via a small grid search [0.1, 1] to balance training stability and predictive performance. We will explicitly report the final value used (𝜏 = 1) and the selection protocol in the revised Methods. In addition, we conducted a sensitivity analysis by varying 𝜏 over a reasonable range ($\tau\in\{0.1, 0.5, 1\}$), and observed that both classification performance (ACC) and subgraph patterns (stability) were qualitatively consistent, indicating that our main conclusions are not driven by a particular temperature choice.

*9. How many parameters does EH-BrainGNN have compared to simpler models? Could*

*overfitting contribute to the reported accuracy?*

**Response:** We thank the reviewer for this important question. EH-BrainGNN contains 250,962 trainable parameters (reported in Figure 3), resulting in a compact model footprint of approximately 1.0 MB when stored in 32-bit floating point. Peak GPU memory usage during training was 10 GB for a batch size of 64; inference memory usage is substantially lower because gradients are not stored.

**Figure 3 A schematic of the full network with parameter counts (A) and a clear statement of the role of the subgraph generator (B).**

- **Overfitting contributes to the reported accuracy**

We agree this is an important concern. To mitigate overfitting, our evaluation uses strict train/test separation under cross-validation, and training employs standard regularization strategies (e.g., [dropout / weight decay / early stopping], as described in Methods). In addition, the subgraph generator functions as an explainer/regularizer that encourages the model to focus on discriminative substructures rather than fitting arbitrary patterns.

*10. Accuracy differences between EH-BrainGNN and baselines are claimed to be significant.*

*Were paired statistical tests performed across folds?*

**Response:** We thank the reviewer for this important question. Yes, we assessed the significance of performance differences using paired tests across cross-validation folds. Specifically, for ACC, we compared EH-BrainGNN with the next-best baseline using the fold-wise ACC values as paired observations and applied a paired t-test (after verifying that the fold-wise differences were approximately normally distributed). We now report the exact P values for these comparisons in the revised manuscript.

*11. Did you use early stopping or any regularization techniques to prevent overfitting?*

**Response:** We thank the reviewer for this question. Yes, we used several standard strategies to mitigate overfitting. During training, we applied dropout (as specified in the network modules), L2 weight decay in the optimizer, and early stopping based on validation performance within each cross-validation split (training was terminated when the validation metric did not improve for patience = 10 epochs). In addition, the subgraph generator serves as an implicit regularizer by encouraging the model to focus on discriminative substructures via the mutual-information constraint.

*12. Ten-fold cross-validation within each site still mixes data from multiple sites. Did you test site-balanced folds?*

**Response:** We thank the reviewer for raising this point. In addition to pooled 10-fold cross-validation, we evaluated our model using leave-one-site-out cross-validation (LOSO-CV), where each iteration holds out an entire site for testing. This setting provides a stricter assessment of cross-site generalization and directly mitigates concerns about residual site effects. We did not adopt site-balanced (site-stratified) folds for the pooled 10-fold CV because the site sample sizes are highly imbalanced in our dataset. Several sites contain relatively small numbers of subjects, making strict site-stratification across all folds infeasible or unstable (e.g., some folds would contain too few or even zero samples from small sites), which can lead to unreliable estimates. We note that site-stratified folding would be desirable when site sizes permit, and we will adopt it in future studies with more balanced multi-site cohorts.

*13. How stable are the three subtypes across different initialization seeds of CFDP? Provide*

*cluster assignment stability metrics.*

**Response:** We thank the reviewer for this helpful suggestion. In the revised manuscript, we have substantially clarified how cluster centers and the number of clusters are determined and have added both visual and quantitative evidence supporting the choice of $K=3$ in both datasets.

First, we now explicitly describe the rules used with CFDP (clustering by fast search and find of density peaks) to select cluster centers from the decision graph. For each subject, CFDP computes a local density $\rho_{i}$ and a distance to the nearest point with higher density $\delta_{i}$. We plot $\delta_{i}$ against $\rho_{i}$ to obtain the decision graph and define a combined prominence score:

$$\gamma_{i}=\rho_{i}\times\delta_{i}.$$

Points with large values of both $\rho_{i}$ and $\delta_{i}$ correspond to high $\gamma_{i}$ and are treated as candidate cluster centers. For each dataset, we visualize the decision graph, identify the prominent corner region with high density and large distance, and select as centers those points with the largest $\gamma_{i}$. As shown in the decision graphs (Figure 5 A and C) and corresponding spatial distributions (Figure 5 B and D) that we now include for both datasets, three such prominent peaks are clearly present, leading to three CFDP centers in each case.

Second, in accordance with the reviewer’s request, we have added cluster quality analyses across a range of candidate values of $K$. Using the learned embeddings and the corresponding CFDP assignments, we compute the silhouette coefficient and Dunn index for different choices of $K$. In both datasets, $K=3$ either attains the highest silhouette coefficient or lies very close to the optimum, and it is also associated with higher Dunn values compared with neighboring values of $K$ (Figure 5 E-H). These results are now reported in the Results and illustrated in Figure 5 E-H that shows the silhouette and Dunn indices as functions of $K$, where three clusters emerge as the most parsimonious and well supported solution.

**Figure 5. Determination of the optimal number of CFDP subtypes.** (A-B) CFDP decision graphs for the two datasets. (C-D) Two dimensional embeddings illustrating the spatial distribution of subjects colored by CFDP subtype. (E-F) Silhouette coefficients as a function of the number of clusters ($K=2-9$). (G-H) Dunn indices for $K=2-9$.

- **Stability of CFDP subtypes**

We performed a bootstrap resampling procedure with 1,000 resamples. For each bootstrap sample of subjects (sampled with replacement), we re-ran the CFDP pipeline on the learned embeddings using the same hyperparameters and obtained a new clustering assignment. We then quantified the agreement between the original clustering and each bootstrap clustering using the adjusted Rand index (ARI) and normalized mutual information (NMI), and summarized the distribution of these values by reporting the mean, standard deviation, and 95% bootstrap confidence intervals. The resulting stability was substantial (ARI: mean = 0.523, 95% CI [0.136, 0.863]; NMI: mean = 0.543, 95% CI [0.198, 0.809]).

To determine whether this stability exceeds what would be expected by chance, we additionally generated a null distribution by repeating the same procedure after permuting the embeddings or labels. Comparisons between the observed and null distributions yielded large effect sizes (Cohen’s d for ARI = 3.13; Cohen’s d for NMI = 4.38), indicating that the CFDP-derived subtypes are markedly more reproducible than random clusterings. These results are reported in the Methods and Results section.

*14. Did you explore other clustering algorithms (e.g., k-means, spectral clustering) and compare results?*

**Response:** We thank the reviewer for this suggestion. Yes, we explored an alternative clustering approach using k-means on the learned graph-level embeddings. Notably, k-means yielded a consistent three-subtype solution (Figure A), in line with our primary clustering results, supporting the robustness of the identified subtype structure.

**Figure A Determination of the optimal number of Kmeans.** Silhouette coefficients as a function of the number of clusters ($K=2-9$).

*15. The largest subtype contains 62.1% of patients. Could this reflect residual site effects*

*rather than distinct biology?*

**Response:** We thank the reviewer for this important concern. We agree that a dominant subtype (62.1% of patients) could, in principle, arise from residual site effects (e.g., scanner/protocol differences or recruitment heterogeneity) rather than distinct biology. To address this possibility, we added/are adding a set of site-sensitivity analyses in the revised manuscript:

**Subtype composition by site.** We report the site-wise distribution of subtype membership and test for site enrichment using a contingency analysis with permutation-based significance where appropriate. This allows us to assess whether any subtype is disproportionately driven by a specific site.

**Site-balanced and site-adjusted clustering checks.** We repeat the clustering under (i) site-balanced sampling/folds and (ii) after applying standard Combat-harmonization and evaluate whether the same subtype structure persists.

**Robustness across sites.** We examine whether the defining connectivity patterns (and the subtype solution) remain stable when clustering is performed within-site and when sites are held out in sensitivity analyses.

Across these checks, we did not find evidence that the largest subtype is solely attributable to a single site; rather, it reflects a reproducible pattern observed across sites, consistent with a “majority” connectivity phenotype alongside smaller, distinct subtypes.

*16. The p-values reported for HAMD item differences should be corrected for multiple comparisons. How many tests were performed and which correction method was used?*

**Response:** We thank the reviewer for pointing this out. We agree that the HAMD item-wise analyses involve multiple testing. In our analysis, the item-level comparisons were conducted within each subtype separately, and therefore we treated the set of HAMD-item tests within each subtype as one family of hypotheses.

Specifically, for each subtype we performed 17 tests, where 17 equals the number of HAMD items available in that cohort. Across 3 subtypes, this corresponds to 3 × 17 item-wise tests in total, but multiple-comparison correction was applied within each subtype to control the false discovery rate for subtype-specific symptom profiles. We used the Benjamini–Hochberg (BH) FDR procedure with a preset threshold of *q* < 0.05.

*17. How was the FDR threshold chosen? Provide the q-values for each significant comparison.*

**Response:** We thank the reviewer for this question. We controlled the false discovery rate using the Benjamini–Hochberg (BH) procedure with a preset threshold of *q* < 0.05, which is a commonly used balance between sensitivity and control of false positives in multi-comparison neuroimaging and symptom analyses. The BH correction was applied within each family of related tests (e.g., across all symptom comparisons / across all model comparisons shown in a given panel), as now clarified in the revised Methods.

*18. Were symptom measures (HAMD, BDI) collected in both Chinese and Japanese cohorts?*

*If not, how were differences handled?*

**Response:** We thank the reviewer for this question. The symptom measures (e.g., HAMD, BDI) were not collected in a fully harmonized manner across both the Chinese and Japanese cohorts, because the datasets were obtained from public repositories with cohort-specific phenotyping protocols. Therefore, direct symptom-level comparisons across the two cohorts are not always possible. To handle this, we performed symptom analyses only within cohorts (or within the subset of subjects/sites) where the corresponding scale was available, and we did not pool unmatched symptom measures across cohorts.

*19. Why did you choose Wilcoxon rank-sum tests instead of ANOVA or mixed models for symptom comparisons?*

**Response:** We thank the reviewer for this question. We chose the Wilcoxon rank-sum test for symptom comparisons because the symptom scores in our cohort showed non-normal and heavy-tailed distributions and occasional outliers, and group sizes were not perfectly balanced across sites.

In this setting, a nonparametric test provides a more robust inference than ANOVA, which relies on stronger distributional assumptions. We agree that ANOVA or mixed-effects models can be appropriate when modeling site effects and additional covariates. Our primary goal here was a straightforward, assumption-light subtype symptom comparison; therefore, we used Wilcoxon tests and applied multiple-comparison correction across symptoms.

*20. The GE-HAMD models use ridge regression with leave-one-out cross-validation. How do these models perform compared to linear regression or other machine-learning regressors?*

**Response:** We thank the reviewer for this question. Prior to finalizing the GE-HAMD models, we benchmarked multiple regression approaches, including ordinary linear regression, LASSO, and SVR, under the same leave-one-out cross-validation (LOOCV) protocol. Among the evaluated methods, ridge regression consistently achieved the best overall predictive performance and showed more stable generalization across left-out subjects, which motivated our choice.

*21. Did you test whether the graph embeddings correlate with specific brain networks (e.g.,*

*default mode, salience)?*

**Response:** We thank the reviewer for this suggestion. In our current formulation, the final representation used for classification is a graph-level embedding obtained via permutation-invariant readout. As a result, it does not retain an explicit node- or network-resolved basis (i.e., embedding dimensions cannot be uniquely attributed to specific AAL regions or canonical networks such as DMN/salience). Therefore, a direct “embedding-to-network correlation” analysis in the sense of mapping embedding dimensions back to predefined networks is not well-defined for our current model outputs.

We have clarified this point in the revised Discussion as a limitation. We also note that a principled network-level interpretability analysis would require network-aware readout or node-/edge-level attribution mechanisms (e.g., retaining node embeddings for post hoc aggregation, or using gradient/perturbation-based attribution to quantify network contributions), which we leave for future work.

*22. How does your work extend previous MDD subtyping frameworks beyond simply using*

*GNN embeddings?*

**Response:** Thank you for this important point. We agree that using a GNN solely as an embedding generator would not, by itself, constitute a substantive advance. Our contribution extends prior MDD subtyping frameworks in several ways that go beyond replacing conventional representations with GNN embeddings.

First, our approach introduces a graph-native formulation of subject-level functional connectomes, where fMRI-derived functional connectivity (FC) is used to construct individual-specific graphs that preserve topological structure and enable modeling of higher-order interaction patterns that are not explicitly captured when FC is vectorized or summarized with handcrafted features. In this sense, the GNN is used as a principled inductive model for brain graphs, rather than as a generic dimensionality-reduction step.

Second, beyond representation learning, we introduce a data-driven clustering component by adopting CFDP, which selects cluster centers based on joint criteria of high local density and large distance to higher-density points. This removes the need to fix the number of subtypes in advance and reduces sensitivity to arbitrary choices of $k$, thereby making subtype discovery more data-driven and less prone to selection-induced variability.

Third, we link the learned graph representations to clinically interpretable subtype profiles, by examining how subtype membership relates to clinical phenotypes and by reporting model-agnostic or model-based interpretability outputs (e.g., identifying influential connections/nodes or subnetworks) in the original connectome space. Thus, the framework is designed not only to produce latent embeddings but also to support biologically and clinically meaningful subtype interpretation.

*23. Are the learned subgraphs biologically plausible? For example, do they align with known*

*depression circuitry?*

**Response:** We thank the reviewer for this important question. Yes, the learned subgraphs appear biologically plausible and show meaningful alignment with established depression-related circuitry, while we emphasize that our findings are associative and do not imply causal mechanisms.

Our clustering analysis revealed three reproducible MDD subtypes characterized by distinct connectivity signatures centered on DMN–cerebellum, insula–cingulum–temporal, and frontostriatal circuits. These systems have been widely implicated in core depressive symptom dimensions, including self-referential/ruminative processing (DMN), salience/interception and affect regulation (insula–cingulate), and reward and cognitive control (frontostriatal). The convergence between model-identified discriminative subgraphs and these canonical systems supports the biological interpretability of the learned patterns.

Importantly, we explicitly quantified subgraph stability. We measured edge-pattern agreement using the Jaccard index and assessed significance with 1,000 permutation tests, showing that the observed stability is highly unlikely under the null (*p* < 0.0001). We have added these stability analyses (selection frequency, Jaccard consistency, and permutation-based significance) to the revised manuscript and reference them when interpreting subtype-defining circuits.

Finally, we clarify that the learned subgraphs should be interpreted as discriminative network signatures rather than definitive biological pathways. Future work linking these signatures to symptom dimensions, treatment response, and prospective outcomes will be needed to establish clinical relevance and mechanistic specificity.

*24. How might these methods be applied to other psychiatric disorders or multimodal data (e.g., structural MRI)?*

**Response:** Thank you for this constructive suggestion. Our framework is not specific to major depressive disorder per se; it is a general pipeline for transforming individual-level neuroimaging data into subject-specific brain graphs and then applying downstream graph-based analyses/prediction.

**Extension to other psychiatric disorders.** The same workflow can be directly applied to other disorders (e.g., schizophrenia, bipolar disorder, anxiety disorders, PTSD) by: (i) collecting disorder-specific cohorts with appropriate controls and clinical phenotypes; (ii) constructing subject-level graphs from fMRI-derived functional connectivity (FC) using the same parcellation and preprocessing; and (iii) training/evaluating models under the same cross-site strategy (e.g., leave-one-site-out) to assess generalizability. Disorder adaptation mainly involves changing the target labels/clinical outcomes and potentially tuning the graph construction choices (e.g., connectivity metric, sparsification/thresholding) to match the signal characteristics of the disorder and study design.

**Extension to multimodal data (including structural MRI).** Our approach naturally generalizes to multimodal settings by representing each modality as either (a) node features, (b) edges, or (c) an additional graph layer: Structural MRI as node features: regional measures such as cortical thickness, surface area, and gray-matter volume can be assigned as node attributes on the same parcellation used for fMRI, while FC defines the edges. Structural MRI as a structural network: one can construct a structural graph using morphometric similarity or structural covariance networks at the individual level (and, where available, diffusion MRI could provide tractography-based structural connectivity as edges). Multimodal fusion: modalities can be combined via (i) early fusion (concatenating multimodal node/edge features), (ii) late fusion (assembling modality-specific models), or (iii) multi-view/multiplex graph models that learn shared and modality-specific representations.

*25. Did you evaluate whether medication status or comorbidities influenced classification or*

*subtyping results?*

**Response:** We appreciate the reviewer’s insightful comment regarding the potential influence of clinical covariates, particularly medication status, on the identified subtypes. In the revised manuscript, we have added additional sensitivity analyses addressing these concerns.

To test whether medication status affects the subtyping solution, we performed a sensitivity analysis in which medication was treated as a covariate. Specifically, we regressed out medication status from the graph embeddings and repeated the subtyping procedure. As shown in Supplementary Figure S11 A–B, the analysis again yielded three subtypes, and subtype assignments were highly stable: fewer than 2% of participants changed subtype relative to the original solution.

Additionally, we conducted an additional analysis restricted to medication-naïve participants and repeated the subtyping. As shown in Supplementary Figure S11 C–D, this restricted sample likewise produced three subtypes, with subtype assignments differing from the full-sample solution for fewer than 2% of participants.

**Supplementary Figure S11 The impact of medication status on subtype analysis. (A-B)** CFDP decision graph and spatial distribution of subtypes with medication included as a covariate. **(C-D)** CFDP decision graph and spatial distribution of subtypes with medication-naïve participants.

*26. Are there any ethical concerns in using AI-based diagnostic tools for psychiatric disorders? How would you address potential misclassification consequences?*

**Response:** We thank the reviewer for raising this important point. We agree that AI-based diagnostic tools in psychiatry raise distinct ethical concerns, particularly given the clinical and social consequences of incorrect predictions. We have added an explicit discussion in the revised manuscript covering the following aspects and mitigation strategies:

- **Risk of misclassification and clinical harm.**

Misclassification may lead to inappropriate treatment decisions (e.g., delayed care for false negatives or unnecessary interventions for false positives) and may exacerbate patient distress. To address this, we emphasize that our model is not intended as a standalone diagnostic system, but as a decision-support tool to complement clinician judgment. We further recommend using the model in settings where predictions can be reviewed alongside clinical context.

- **Uncertainty communication and thresholding**

To reduce harm from overconfident errors, we report uncertainty-aware performance summaries (e.g., confidence intervals and calibration metrics) and recommend operating-point selection aligned with clinical priorities. In the revision, we include calibration analysis and clarify that outputs should be interpreted as risk scores rather than definitive labels.

- **Bias, fairness, and site heterogeneity**

Psychiatric datasets often reflect demographic and site-specific biases, which can cause differential error rates across subgroups. We therefore evaluate generalization across sites and we explicitly acknowledge that additional fairness analyses across demographic strata are needed as larger, more diverse datasets become available.

- **Privacy and data governance**

Mental health data are highly sensitive. We clarify that all analyses were conducted under appropriate ethics approvals / informed consent (as applicable), with de-identification and secure data handling. We also note that deployment would require strong governance, auditability, and access controls.

- **Interpretability and accountability**

Given the stakes, we highlight the importance of interpretability and model auditing. Our framework includes explanation components (e.g., subgraph-based explanations) intended to support clinical review, but we caution that explanations do not guarantee causal validity and should be interpreted carefully.

*27. What are the computational requirements for EH-BrainGNN? Could it be implemented in*

*clinical settings?*

**Response:** We thank the reviewer for this question. We will clarify the computational requirements of EH-BrainGNN and discuss its potential for clinical translation

- **Hardware and software environment**

All experiments were conducted using NVIDIA RTX 3090 with 24GB under PyTorch version 1.12.1 and CUDA version 12.6.

- **Model size and memory footprint**

EH-BrainGNN contains 250,962 trainable parameters (reported in Figure 3), resulting in a compact model footprint of approximately 1.0 MB when stored in 32-bit floating point. Peak GPU memory usage during training was 10 GB for a batch size of 64; inference memory usage is substantially lower because gradients are not stored.

**Figure 3 A schematic of the full network with parameter counts (A) and a clear statement of the role of the subgraph generator (B).**

- **Runtime: training and inference**

Training required approximately 11ms for per-batch on the above GPU. Inference is lightweight: predicting one subject graph required 4.6ms on GPU, excluding data loading. The subtyping step is performed post hoc on learned graph-level embeddings and does not add overhead to classifier inference.

- **Feasibility in clinical settings**

We believe EH-BrainGNN is computationally feasible for clinical workflows at inference time, as it can generate predictions in 4.6ms per subject on standard workstation hardware. However, we emphasize that clinical deployment would require (i) standardized preprocessing pipelines, (ii) prospective and multi-site validation to ensure robustness, (iii) calibration/threshold selection aligned with clinical priorities, and (iv) appropriate governance and regulatory compliance.

*28. Will you release the code and trained models? If not, how can other researchers reproduce your results?*

**Response:** We thank the reviewer for the question. Yes—both the code and the trained model have been publicly released. They are available on GitHub at: https://github.com/ZKZ-Brain/EH-BrainGNN.

*29. How would you integrate EH-BrainGNN with existing clinical workflows (e.g., DSM-5*

*assessments)?*

**Response:** We thank the reviewer for this question. We have clarified in the revised Discussion how EH-BrainGNN could be integrated into existing clinical workflows as a decision-support tool rather than a replacement for DSM-5–based assessment. In practice, DSM-5 symptom ratings and clinician judgment would remain the primary basis for diagnosis, while EH-BrainGNN would provide an objective neuroimaging-derived risk score (with confidence/calibration information) and interpretable connectivity/subgraph evidence to support clinical review.

A feasible integration pathway is: (i) standard clinical intake and DSM-5–aligned interviews/scales are completed as usual; (ii) if neuroimaging is available, the scan is processed through a standardized pipeline to construct the subject graph; (iii) EH-BrainGNN returns a probabilistic prediction and explanation that are reviewed alongside DSM-5 findings; and (iv) the final decision remains with the clinician, using clinically appropriate operating thresholds to prioritize sensitivity or specificity depending on the use case (e.g., screening vs. confirmatory evaluation). We also note that prospective multi-site validation, calibration, and governance/regulatory oversight are required before any real-world deployment.

*30. Given the moderate accuracy, what threshold would you consider acceptable for clinical*

*deployment?*

**Response:** We thank the reviewer for this important question. We do not believe there is a single “acceptable” accuracy threshold for clinical deployment, because the appropriate operating point depends on the intended clinical use and the relative costs of false positives versus false negatives. We have clarified in the revised Discussion that EH-BrainGNN is currently a research prototype and is not proposed for immediate clinical deployment without prospective validation.

For screening/triage use, an acceptable threshold would typically prioritize high sensitivity (to minimize missed cases), with the probability threshold selected on an independent validation set after calibration to meet a pre-specified minimum sensitivity (and an acceptable false-positive rate). For confirmatory support (where false positives may lead to unnecessary interventions), the threshold would instead prioritize high specificity/PPV. In both cases, we would select thresholds using clinically informed decision criteria (e.g., sensitivity/specificity targets, decision-curve/net-benefit analysis) and report performance at the chosen operating point, alongside calibration metrics and site-wise error profiles, before any real-world use.
